# The diagnosis of ASD with MRI: a systematic review and meta-analysis

**DOI:** 10.1038/s41398-024-03024-5

**Published:** 2024-08-02

**Authors:** Sjir J. C. Schielen, Jesper Pilmeyer, Albert P. Aldenkamp, Svitlana Zinger

**Affiliations:** 1 Department of Electrical Engineering, Eindhoven University of Technology, Eindhoven, the Netherlands; 2 Department of Behavioral Sciences, Epilepsy Center Kempenhaeghe, Heeze, the Netherlands

**Keywords:** Diagnostic markers, Molecular neuroscience

## Abstract

While diagnosing autism spectrum disorder (ASD) based on an objective test is desired, the current diagnostic practice involves observation-based criteria. This study is a systematic review and meta-analysis of studies that aim to diagnose ASD using magnetic resonance imaging (MRI). The main objective is to describe the state of the art of diagnosing ASD using MRI in terms of performance metrics and interpretation. Furthermore, subgroups, including different MRI modalities and statistical heterogeneity, are analyzed. Studies that dichotomously diagnose individuals with ASD and healthy controls by analyses progressing from magnetic resonance imaging obtained in a resting state were systematically selected by two independent reviewers. Studies were sought on Web of Science and PubMed, which were last accessed on February 24, 2023. The included studies were assessed on quality and risk of bias using the revised Quality Assessment of Diagnostic Accuracy Studies tool. A bivariate random-effects model was used for syntheses. One hundred and thirty-four studies were included comprising 159 eligible experiments. Despite the overlap in the studied samples, an estimated 4982 unique participants consisting of 2439 individuals with ASD and 2543 healthy controls were included. The pooled summary estimates of diagnostic performance are 76.0% sensitivity (95% CI 74.1–77.8), 75.7% specificity (95% CI 74.0–77.4), and an area under curve of 0.823, but uncertainty in the study assessments limits confidence. The main limitations are heterogeneity and uncertainty about the generalization of diagnostic performance. Therefore, comparisons between subgroups were considered inappropriate. Despite the current limitations, methods progressing from MRI approach the diagnostic performance needed for clinical practice. The state of the art has obstacles but shows potential for future clinical application.

## Introduction

Autism spectrum disorder (ASD) is a spectrum of neuropsychiatric disorders that typically comes paired with challenges in social interaction. Children with ASD tend to show repetitive behavior in activities and interests and deficits in communication and reciprocal ability. Though most visible during childhood, the effects of ASD persist for a lifetime [1].

The prevalence of ASD is estimated to be one in a hundred people worldwide [2]. As individuals with ASD lag behind their healthy peers in development, they are at a higher risk of comorbidities such as depression, stress, and anxiety [1, 3]. This burden not only affects the individual with ASD but is known to spill over to the caregiver [3–6]. Carrying this burden comes with healthcare costs estimated to range from $2.4 million to $3.2 million (US$) over the lifetime of an individual [7].

After its first description in 1943, the definition of autism has changed many times over the years [8, 9]. Currently, the fifth edition of the Diagnostic and Statistical Manual of Mental Disorders (DSM-5) is the standard handbook for authoritative guidance in the diagnosis of mental disorders, among which ASD, in several countries, including the United States [1]. The diagnostic criteria in the DSM-5 have been debated, specifically during the revision period leading up to its publication. A significant part of the debate finds its root in the subjectivity surrounding the observation-based diagnosis [9, 10]. Observation-based diagnosis has been agreed to be imperfect, which is why the National Institute of Mental Health (NIMH) called for the paradigm to change toward a diagnosis based on analyses progressing from objective measurements [11, 12].

Magnetic resonance imaging (MRI) offers a noninvasive, high-level measurement of the brain from which analysis can progress toward clinically relevant variation used for diagnosis. Many studies have explored the dichotomous classification between individuals with ASD and healthy control subjects (HCs) using different modalities of MRI scans: functional MRI (fMRI), structural MRI (sMRI), and diffusion MRI (dMRI; i.e., diffusion tensor imaging) [13, 14]. A partly similar review that focused only on resting-state fMRI (rsfMRI) was conducted by Santana et al. (2022) [15]. In their review, they presented that the number of included studies exponentially increased up until the year 2019. If this trend continues, each subsequent year will contain more studies. Therefore, including the 3 succeeding years results in a significant increase in papers, which motivates an update. We also aim to expand by incorporating different MRI modalities. Furthermore, we wholly adhered to the Preferred Reporting Items for Systematic Reviews and Meta-Analyses (PRISMA) 2020 guidelines [16].

In this study, we mainly aim to describe the state of the art of diagnosing ASD using MRI in terms of aggregated diagnostic performance metrics. We also aim to compare imaging modalities and analyze statistical heterogeneity.

## Methods

### Registration

This review was registered at Open Science Framework Registries under registration 10.17605/OSF.IO/DRS3Q. The protocol, amendments, and explanations are provided in the registry. The scripts central to the conclusions drawn in this review are also provided in the registry.

### Eligibility criteria

Two reviewers (SJCS and JP) independently screened and selected peer-reviewed, cross-sectional studies that described dichotomous, individual-level classification between individuals with ASD and HCs through an analysis progressing from data obtained from a resting-state MRI scan. We made the decision to exclude task-based studies. While task-based fMRI merits research in the field of neuropsychiatric disorders, we deem the factor of analysis to be leading. It is an attempt to limit the heterogeneity between the tasks subjects are instructed to perform, which is more consistent for studies where participants perform the resting task. Furthermore, the studies had to be written in English and published between January 1, 2018, and December 31, 2022. Studies had to report information on their included participants (at least the number of individuals with ASD and HCs) and how their sample was used for training, validation, and testing, along with the resulting diagnostic accuracy, sensitivity, and specificity. Conflicts concerning a study’s eligibility were resolved by consensus. An overview of the eligibility criteria is available in the registration protocol and in Supplementary Materials A1.

The included studies were used for statistical syntheses in the following divisions. Besides synthesizing results on all studies combined, subgroups were made for syntheses in two ways: per modality (dMRI, rsfMRI, sMRI, multimodal) and whether the data was obtained from single or multiple imaging sites. In a post hoc analysis to assess the performance of different features, studies that used Pearson-correlation-based functional connectivity were compared to studies that applied the Fisher transformation to the correlation values, as Santana et al. found that the Fisher transformation leads to significantly better sensitivity and specificity [15].

### Source and search

Web of Science and PubMed were used to conduct searches, of which the last occurred on February 24, 2023, for both databases. The following search term was used: *(“Autism” OR “Asperger” OR “autism spectrum disorder” OR “ASD”) AND (“classification” OR “machine learning” OR “SVM” OR “NN” OR “prediction” OR “deep learning” OR “computer-aided diagnosis”) AND (“magnetic resonance imaging” OR “MRI”)*, where the search range was set between January 1, 2018, and December 31, 2022. We used Web of Science’s option to exclude reviews from the search results.

### Selection and collection

A template was made of the relevant information (variables) to extract from the studies. A consensus was reached on the template before data collection. Afterward, the information specified by the template was extracted from included reports by two reviewers (SJCS and JP) independently. Discrepancies in the extracted variables were resolved by discussion.

If a study reported multiple experiments, only the experiments conducted on different datasets were included to avoid a disproportionate weight in the analyses. If various experiments on the same dataset were reported, the results of the experiment outlined by the authors were used. If the authors did not outline a result, the experiment that yielded the best diagnostic accuracy was selected.

### Data items

The data items that were extracted from the studies span the following domains: the dataset (sample size, demographics, whether it is obtained at one or more sites), features (atlas, type of features, type of processing, and dimension of feature vector), the classifier (type and validation method), performance metrics (sensitivity, specificity, and accuracy). The year of publication, number of experiments, and imaging modality were also collected. The performance metrics are the most important to the review’s conclusions, as it is considered most in the statistical syntheses. A full overview of the data extraction form is present in the registry and in Supplementary Materials A2. The time frame was not considered, as per the selection criteria only cross-sectional experiments were eligible. If studies did not report the sensitivity, specificity, and accuracy, additional information that allows these to be calculated was sought in the paper. The information needed to calculate the required performance metrics is given in the registry and in Supplementary Materials A1.

### Study assessment

The included studies’ risks of bias and quality were assessed on four domains: patient selection, index test, reference standard, and flow and timing, as we used the revised Quality Assessment of Diagnostic Accuracy Studies (QUADAS-2) tool [17]. The QUADAS-2 tool consists of questions for each of the domains which help in an assessment of the respective domain’s risk of bias and overall quality. Concerns regarding applicability were also described for the domains of patient selection, index test, and reference standard. The assessments were made by two reviewers (SJCS and JP) independently and discrepancies were resolved by discussion. The QUADAS-2 tool was tailored to this review by adding and deleting some questions from the original template. The domain of the index test, for example, was tailored to assess the risk of overfitting, as too many included features tend to cause overfitting [18, 19]. The template and amendments with motivation can be found in the registry and in Supplementary Materials A3.

A judgment that summarized the studies’ risk of bias was made per domain. The overall judgment was mainly based on the majority of the answers: a majority of yeses, nos, or “unclears”, respectively resulted in a low, high, or unclear risk of bias assessment. If there was no majority, the overall assessment is unclear.

One exception was made for studies where only data from a single site were used to validate results based on a single train-test split. We gave this a high risk of bias for the index test, as there is a risk that the reported result was based on a lucky split which could have been avoided by using cross-validation. If a study trained its classifier on data obtained from one site and then tested it on data obtained from another site, we considered this risk lower for the following reasons. The split is transparent and testing on the data of a different site introduces variation through different scanning parameters, which has an adverse effect on the performance of overfitted classifiers.

### Meta-analysis

We used Reitsma’s bivariate random-effect model to analyze aggregated diagnostic performance metrics [20]. The reported performance metrics and group sizes were used to calculate confusion matrices required for the mada (Meta-Analysis of Diagnostic Accuracy) package in R [21, 22]. Using this model, we obtained the following statistical syntheses: the sensitivity, specificity, summary receiver operator characteristics curve (SROC), and the area under the SROC (AUC). These results were obtained for the specified groups: all experiments, subgroups per modality, and whether single or multiple imaging sites were used. All included studies were eligible for synthesis due to the eligibility criteria.

To assess the variation that cannot be attributed to sampling (i.e. heterogeneity), we used Higgins’ ${I}^{2}$-statistic computed on the diagnostic odds ratios (DORs) obtained with a univariate random-effect model [23]. As objective measures of heterogeneity have been argued to be conservative for accuracy studies or ’confounded’ by sample size, we supplemented the objective measure with a visual inspection of both the univariate random-effect forest plot and the prediction region of the bivariate random-effect SROC [24, 25]. The confidence regions for bivariate syntheses and confidence intervals of DORs were computed at 95% confidence. We also performed a sensitivity analysis by excluding studies that were judged high risk in any of the QUADAS-2 domains, as this bias limits certainty in the evidence of an outcome. Potential sample size effects or publication bias were assessed using Deek’s test [26].

## Results

### Study selection and characteristics

**Fig. 1:**
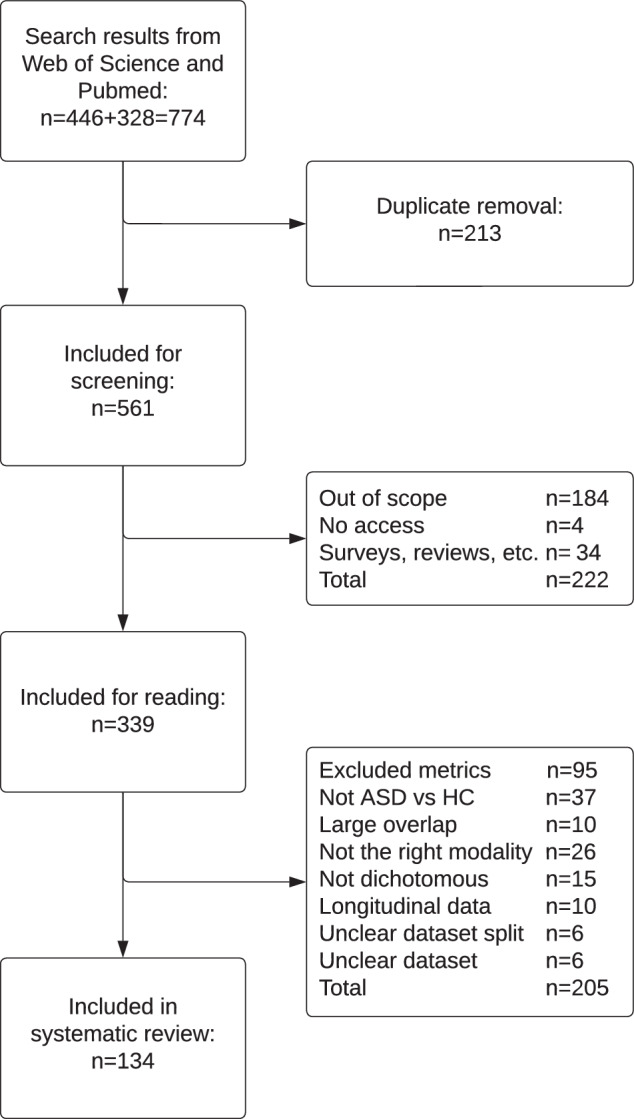
A flowchart of the selection process, where *n* indicates the number of studies. Made using https://www.lucidchart.com.

The manual selection process is shown in Fig. 1. Our search yielded 774 results, of which 561 were screened after duplicate removal. After the screening, 339 studies were included for reading, of which 134 were included in the review [27–160]. The most common reason for exclusion was the absence of the required metrics or sufficient information that allows these to be calculated. In this excluded study, for example, only the AUC and accuracy were reported [161]. Some studies did show the required metrics but in a figure rather than explicitly in a numeric expression, e.g., [162]. These studies were also excluded. The following studies reported more than one experiment eligible for inclusion [28, 29, 49, 52, 57, 60, 62, 67, 68, 74, 77, 79, 85, 93, 121, 129]. A total of 159 experiments were included.

A plot of the included studies per year and per modality is provided in Supplementary Materials A4. The number of included studies increased every year. The characteristics of each experiment are listed in Supplementary Materials B1. It was not possible to precisely determine the number of unique participants aggregated over the included studies because some studies did not mention which participants they selected from publicly available datasets. By summing the largest samples per dataset used in an included experiment, we conservatively estimated the number to be 4982 unique participants consisting of 2439 individuals with ASD and 2543 healthy controls. Of the included experiments, 88.1% used the Autism Brain Imaging Data Exchange (ABIDE), 5.7% used an in-house sample, 3.1% used the National Database for Autism Research (NDAR), and 3.1% used other datasets [163, 164]. A pie chart with an overview is provided in Supplementary Materials A5. Since ABIDE was used in most of the included studies, there likely was an overlap between the acquired samples. Overall, 16.2% of the participants included in the experiments were female. As not all studies reported the sex distribution of their sample, this statistic only includes the experiments that mentioned it.

The majority (74.8%) of included experiments were performed on rsfMRI data. Furthermore, 13.8% used sMRI and 3.1% used dMRI. Any combination of modalities fell under the category multimodal, which accounted for 8.2% of the experiments. The number of experiments is plotted per modality in Supplementary Materials A6.

Approval for the studies and informed consent from the subjects are either directly reported in the reviewed papers or the reviewed papers provide a reference to an online dataset. As reviewers, we rely on the accuracy of these reports and we do not have the means to independently verify them.

### Study assessment

**Fig. 2:**
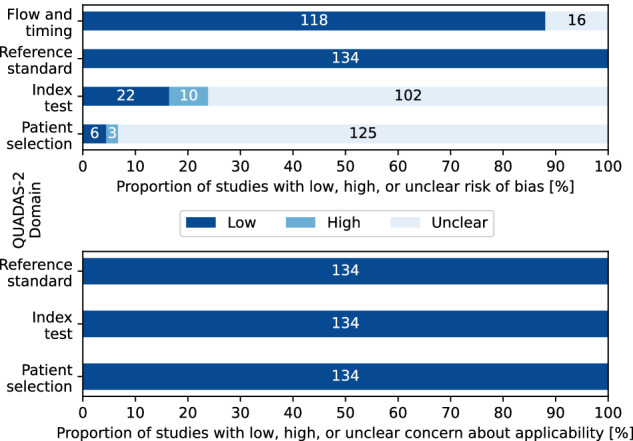
Graphical summary of QUADAS-2 assessments. The top figure shows the risk of bias summary assessments and the bottom figure shows the level of concern summary assessments.

The summary judgments on the risk of bias and the concern regarding applicability for each study are summarized per domain in Fig. 2. These summary judgments were based on answers to the questions of the QUADAS-2 tool, which can be found along with descriptions in Supplementary Materials B2. No concerns about applicability were found, likely as a result of the selection criteria.

The vast majority of studies scored an unclear risk of bias in the domains of the index test and patient selection. In the domain of the index test, we took a conservative approach to be risk-averse to potential overfitting. The validation method was deemed high risk for ten studies, resulting in a summary judgment of high risk. Furthermore, in most studies, the number of features was not specified. This often led to an unclear risk of bias.

In the domain of patient selection, many studies gave a reference to a publicly available dataset without specifying the information required to answer the questions. Since most studies selected participants from the dataset, only the reference to the whole dataset was not reproducible. In this study, for example, subjects were excluded because of ”incomplete brain coverage, high motion peaks, ghosting and other scanner artifacts” [80]. The selection criteria ”high motion peaks” and ”other scanner artifacts” were not reproducible because of subjectivity and incompleteness, respectively. This made it impossible to obtain demographic information like age, sex, or potential comorbidity, even though a reference to ABIDE was provided. Therefore, we only considered the information provided in the papers themselves, which often resulted in questions being answered unclear. In turn, the summary judgment often resulted in unclear.

### Meta-analysis

**Fig. 3:**
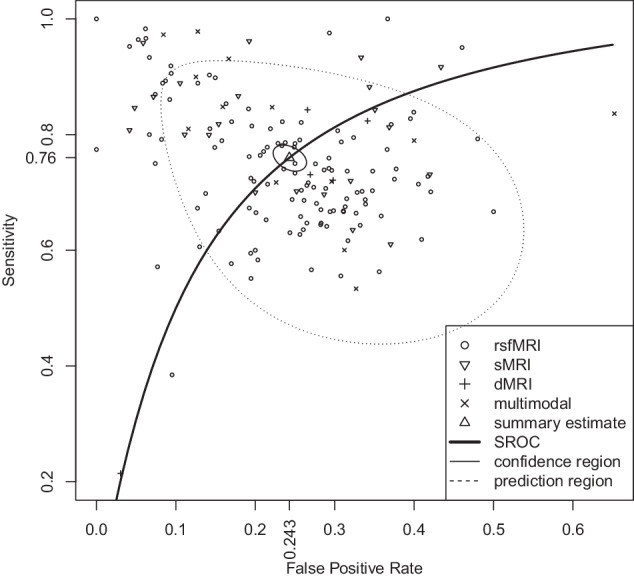
Summary receiver operating characteristics curve for all included experiments, sensitivity 76.0% (95% CI 74.1–77.8), specificity 75.7% (95% CI 74.0–77.4), AUC 0.823. Uncertainty limited the confidence in these results. Note that the axes do not span from 0 to 1 to aid readability. No data was left out of the figure. {rsf, s, d}MRI: {resting-state functional, structural, diffusion} magnetic resonance imaging, SROC summary receiver operating characteristics, AUC area under SROC curve.

The main outcome resulted from pooling over all (159) included experiments. In this quantitative analysis, experiments conducted using different MRI modalities, disjoint and overlapping patient samples, and samples obtained with different scanning parameters were included. This resulted in the following summary estimates for the performance metrics: 76.0% sensitivity (95% CI 74.1–77.8), 75.7% specificity (95% CI 74.0–77.4), and an AUC of 0.823. Uncertainty in study assessment limited the confidence in these results. The SROC curve, along with its 95% confidence region and 95% prediction region, is shown in Fig. 3.

The summary estimates of the performance metrics and the DOR, along with the measure of statistical heterogeneity, are listed for each of the groups used for synthesis in Table 1. Per group, the included studies, the number of studies ($n$) and the number of studies that scored a high risk of bias in any of the QUADAS-2 domains (${n}_{{hrob}}$), and the share of experiments conducted on a multi-site sample are also listed. Note that the number of studies included in the single-site and multisite subgroups does not add up to the total number of studies because two studies did not specify whether multiple or a single site were used [36, 55]. As shown in Fig. 2, only high risks of bias occurred in the domains of the index test and patient selection. A high risk of bias in either of these domains might result in slightly inflated performance metrics, as they might generalize poorly to new patients as a result of selection or being overfitted. A sensitivity analysis was conducted to assess this impact.

**Table 1:** Synthesis outcomes for all experiments combined, different modalities separately, and grouped per whether data was obtained from single or multiple imaging sites.

Group	Sensitivity (CI)	Specificity (CI)	AUC	DOR (CI)	*I*^2^	*n*	*n*_hrob_	share multi-site	Included studies
All	0.760 (0.741–0.778)	0.757 (0.740–0.774)	0.823	9.76 (8.62–11.01)	43.0%	134	13	65.4%	[27–160]
rsfMRI	0.748 (0.727–0.768)	0.754 (0.735–0.771)	0.815	9.01 (7.88–10.29)	45.2%	95	6	77.6%	[28–35, 38–40, 44–47, 49, 52, 54, 56, 57, 62, 64–72, 74, 76–86, 92, 93, 95–99, 101, 102, 106–111, 113, 118–123, 125–142, 144, 146, 147, 150, 152–160]
sMRI	0.799 (0.754–0.838)	0.777 (0.713–0.830)	0.854	13.89 (9.08–21.26)	28.6%	21	3	37.9%	[36, 41, 42, 50, 51, 58–60, 63, 75, 87–89, 91, 103, 104, 115, 117, 124, 143, 145]
dMRI	0.689 (0.549–0.857)	0.758 (0.628–0.854)	0.790	7.24 (3.99–12.10)	0.0%	5	1	0.0%	[55, 105, 114, 148, 151]
multimodal	0.827 (0.742–0.888)	0.777 (0.692–0.844)	0.869	15.77 (8.49–29.33)	48.5%	13	2	76.9%	[27, 37, 43, 48, 53, 61, 73, 90, 94, 100, 112, 116, 149],
single-site	0.791 (0.759–0.819)	0.800 (0.770–0.828)	0.863	16.32 (12.49–21.32)	17.3%	42	6	0.0%	[29, 30, 35, 45, 48, 52, 53, 57, 60–63, 67, 69, 74, 87, 89, 91, 103–106, 109, 114, 117, 121, 124, 126, 131, 134, 140, 143–145, 148, 150, 151, 153, 156, 157, 159, 160]
multi-site	0.747 (0.724–0.769)	0.742 (0.721–0.762)	0.807	8.36 (7.27–9.62)	48.1%	90	6	100.0%	[27, 28, 31–34, 37–44, 46, 47, 49–51, 54, 56, 58, 59, 64–66, 68, 70–86, 88, 90, 92–102, 107, 108, 110–113, 115, 116, 118–120, 122, 123, 125, 127–130, 132, 133, 135–139, 141, 142, 146, 147, 149, 152, 154, 155, 158]

*CI* confidence interval (95%), *{rsf, s, d} MRI* {resting-state functional, structural, diffusion} magnetic resonance imaging, *AUC* area under the summary receiver operator characteristics curve.

*DOR* diagnostic odds ratio, I2 refers to Higgins’ statistic, *n* is the number of included studies, *n*_hrob_ is the number of included studies which scored a high risk of bias.

Note that uncertainty limited the confidence in the results.

We repeated the primary analyses after excluding the studies that scored a high risk of bias in any of the QUADAS-2 domains. The results are listed in Supplementary Materials A7. We observed no significant changes after excluding high-risk studies, as the confidence intervals of the sensitivity, specificity, and DOR significantly overlapped with those listed for the respective groups in Table 1.

The vast majority of studies scored unclear in the domains of the index test and patient selection. If we excluded studies that scored unclear in any of the domains, only two studies remained [73, 117]. We considered these too few for a separate analysis. Due to the extent of uncertainty, we have limited confidence in the results of the syntheses.

Objective measures of heterogeneity (${I}^{2}$) are listed in Table 1. The objective measures suggested moderate heterogeneity in the syntheses of all experiments and the subgroups of rsfMRI, multimodal, and multi-site. The syntheses of the subgroups of sMRI, dMRI, and single-site suggested no important level of heterogeneity [24]. To supplement the objective measures of heterogeneity, we visually inspected the prediction region shown in Fig. 3 and the forest plot of the natural logarithm of the diagnostic odds ratios shown in Supplementary Materials C1. In Fig. 3, 31 (19.5% of) experiments were visually scattered outside of the prediction ellipse, also indicating heterogeneity. The non-overlapping confidence intervals shown in Supplementary Materials C1 further confirmed moderate heterogeneity in the syntheses of all experiments and the subgroups of rsfMRI and multimodal. A larger overlap between the confidence intervals of the subgroup sMRI indicated that less inter-study variation was captured in this synthesis. The subgroup dMRI showed near full overlap in confidence intervals, which indicated no relevant heterogeneity.

When comparing single-site and multi-site experiments, we observed an overlap between the confidence intervals of single-site studies that was larger than the overlap between the confidence intervals of multi-site studies. The larger overlap for single-site experiments was partly aided by larger confidence intervals as a result of smaller sample sizes. The absence of this overlap in multi-site experiments, nevertheless, could not be attributed to sampling and hence was statistical heterogeneity by definition.

Deek’s test indicated a statistically significant negative correlation between the diagnostic odds ratio and sample size in all groups used for synthesis apart from the groups dMRI and single-site (Supplementary Materials A8). After correcting these results for multiple comparisons using Bonferroni correction, only the subgroup of multimodal no longer showed a statistically significant correlation (*P* = 0.034). The negative correlation between the diagnostic odds ratio and sample size indicated the presence of small-study effects.

The results of the post hoc test on whether the Fisher transformation of functional connectivity yields better diagnostic performance than the Pearson-correlation values are listed in Supplementary Materials A9. Based on the studies included in this review, no significantly higher diagnostic odds ratio was observed for studies that use the Fisher transformation (*P* = 0.593).

## Discussion

We aimed to describe the state of the art in diagnosing ASD with MRI and found the overall summary estimates of 76.0% sensitivity (95% CI 74.1–77.8), 75.7% specificity (95% CI 74.0–77.4), and an AUC of 0.823, however, there is limited certainty in these results as the vast majority of studies scored unclear in at least one of the assessment domains. Furthermore, we aimed to compare imaging modalities and analyze statistical heterogeneity.

The modalities included in this review are sMRI, dMRI, rsfMRI, and multimodal, i.e., any combination of the modalities. It is worth mentioning that magnetic resonance spectroscopy (MRS) was originally not considered as we focused on imaging. Still, studies have shown neurometabolic differences between individuals with ASD and healthy controls with MRS [165]. In a post hoc search tailored to MRS with the same selection criteria (the terms magnetic resonance imaging and MRI were respectively replaced with magnetic resonance spectroscopy and MRS in the search term), we explored whether studies used these neurometabolic differences for diagnosis using MRS. This search resulted in 27 studies of which none were eligible. The most common reason for exclusion was evaluation of group differences rather than individual-level classification. While these studies were not eligible per our selection criteria, the neurometabolic differences identified with MRS potentially are a valuable supplement to the aberrations found with MRI.

As listed in Table 1, the degree of heterogeneity captured in the subgroups varied greatly. As objective measures of heterogeneity are debated, we supplemented with visual inspection of the univariate random-effect forest plot (Supplementary Materials C1) and the prediction region of the SROC obtained with the bivariate random-effects model [24, 25]. The visual inspection allowed more certainty in the objective measures, as we observed the percentage described by the ${I}^{2}$-statistic to be proportional to the visual discrepancies between the confidence intervals in the forest plot. While we acknowledged that the obtained ${I}^{2}$-statistics likely do not exactly describe the degree of heterogeneity captured in the syntheses, we deemed it sufficient to draw conclusions based on the visual supplement.

With the observed differences in the degrees of heterogeneity in mind, we did not consider it appropriate to statistically test whether one modality was more favorable than another. We could, for example, compare rsfMRI and sMRI as they were the two most represented modalities in the meta-analysis. One can observe a better outcome for the synthesis of sMRI with a sensitivity of 79.9% (95% CI 75.4–83.8) and specificity of 77.7% (95% CI 71.3–83.0) compared to rsfMRI’s sensitivity of 74.8% (72.7–76.8) and specificity of 75.4% (95% CI 73.5– 77.1). This difference might be statistically significant, but conclusions based on this would not take the captured extent of heterogeneity into account, making it an ill comparison. Out of the experiments included in the synthesis for sMRI, 37.9% were multi-site experiments, whereas the share of multi-site experiments was 77.6% for the synthesis of rsfMRI experiments. There is no guarantee the diagnostic performance found for the synthesis of sMRI experiments will hold up when tested to the extent to which the rsfMRI experiments were tested, which included larger, more heterogeneous samples. Moreover, most studies scored unclear in at least one of the QUADAS-2 assessment domains, which indicates limited confidence in generalization ability.

As the degree of captured heterogeneity varies over the included studies, it is not straightforward to assess what features are most valuable for ASD diagnosis. Santana et al. found higher sensitivity and specificity for studies that used the Fisher transform of functional connectivity compared to studies that did not use the transform in their review of rsfMRI [15]. Therefore, we tested if Fisher transformed functional connectivity also led to better diagnostic performance than the studies that used functional connectivity without Fisher transform in this review. We did not find significantly higher diagnostic performance when studies used the Fisher transform (*P* = 0.593, Supplementary Materials A9). We cannot conclude that the Fisher transform is favorable when using functional connectivity based on the studies we included, but this may be due to the observed heterogeneity.

To analyze part of the observed heterogeneity, we used whether an experiment was conducted on a sample gathered from a single site or multiple sites as a proxy for cross-site heterogeneity. This can be considered a separate subgroup analysis, which was one of the suggested approaches [166]. Unseen data obtained in a different way (e.g., different scanning parameters) were described to introduce variation, which forms an obstacle to the clinical application of machine-learning-based approaches [167, 168]. Listed in the “*I*^2^” and “share multi-site” columns of Table 1, the captured heterogeneity *I*^2^ can be observed to be correlated to the share of multisite experiments.

While this proxy seemed to explain at least a part of the heterogeneity, it came with limitations. The proxy itself is a dichotomization of whether one or multiple sites were included in the experiment’s sample. The degree of heterogeneity caused by different sites is likely proportional to the number of sites. The degrees of captured heterogeneity between multi-site samples varied, which was not accounted for by dichotomizing between experiments conducted on single-site and multi-site samples. This meant that although we expected heterogeneity in all multi-site experiments, the extent differed based on how many different sites were included. This was not accounted for in the dichotomization.

In addition, the proxy only accounted for potential variation between the sites where the samples used for experiments came from. The syntheses on single-site experiments and dMRI experiments, respectively showed an ${I}^{2}$-statistic of 17.3% and 0.0%, which both fell in the range considered not important [24]. Despite that these levels might not be considered important, the captured heterogeneity in the synthesis of single-site experiments showed heterogeneity that cannot be attributed to cross-site effects. This indicates that not all heterogeneity in the multi-site samples can be attributed to cross-site variation. Despite the limitations of the proxy, it explained the observable differences in measured heterogeneity between the syntheses on multi-site and single-site experiments.

We have used the term heterogeneity mostly in the statistical sense in this study, but it is universally accepted as a keyword to describe ASD itself [169]. ASD has been described to be heterogeneous in etiology, phenotypes, and manifestation [170]. Therefore, it is likely that any sample will capture some degree of heterogeneity inherent to ASD, even without the introduction of cross-site effects. This source of heterogeneity is likely captured to a larger extent if individuals with different places on the spectrum are included. If a larger sample is acquired, it is likely that more of the heterogeneity inherent to ASD is captured. The heterogeneity inherent to ASD is a possible explanation of the observed heterogeneity in single-site experiments. Also, the differences in modalities, methodology, and patient selection are possible explanations.

Sex differences in ASD were also observed to add to the inherent heterogeneity [170, 171]. Not all of the included experiments specified the sex distribution of their samples, but the aggregated distribution over experiments that did report the sex distribution is 16.2% female and 83.8% male. This likely is an effect of the imbalance in prevalence, as estimates range from a 2:1 male-to-female ratio to a 5:1 male-to-female ratio [172]. Out of the included experiments, 88.1% used ABIDE, which is mainly supplied by American and European sites. Therefore, a limitation in the evidence is the overlap in the samples, which limits certainty in the generalization ability toward broader gender inclusion and ethnic diversity.

Deek’s test was recommended for meta-analyses of diagnostic test accuracies [26, 173]. We observed a statistically significant negative correlation between diagnostic accuracy and sample size for the synthesis on all experiments and the syntheses of the subgroups rsfMRI, sMRI, and multi-site. No statistically significant correlations were found for the subgroups dMRI and single-site. After Bonferroni correction, the correlation found for the subgroup of multimodal studies was no longer significant. For the plot and *P*-values, please refer to Supplementary Materials A8. The statistically significant correlations showed the presence of small-study effects, as smaller studies tended to have higher diagnostic accuracies than larger studies.

The presence of small-study effects may indicate publication bias. However, the contributions of publication bias and other forms of heterogeneity cannot always be disentangled in meta-analyses [174]. As publication bias is a form of heterogeneity, small-study effects cannot be explained by only looking at the measures of heterogeneity, as cause and effect cannot be disentangled. Considering the proxy for cross-site heterogeneity, however, a clear distinction is observable between subgroups that included multi-site experiments (rsfMRI, sMRI, multimodal, and multi-site) and subgroups that did not (dMRI and single-site). As researchers are limited in their resources, larger samples are likely gathered from multi-site cohorts like ABIDE. As described before, multi-site samples come paired with a larger degree of captured heterogeneity. This is known to have an adverse effect on the performance of machine-learning-based algorithms, which in turn results in lower diagnostic accuracy [167, 168]. While we cannot completely exclude the possible presence of publication bias, we think the observed small-study effects are rather an artifact of cross-site heterogeneity.

We assessed studies using the QUADAS-2 tool of which the full results are listed in Supplementary Materials B2 and the summary judgments in Fig. 2 [17]. The QUADAS-2 tool was tailored to the review. The questions, together with amendments and rationale, are reported in Supplementary Materials A3. A limitation of this approach is that it may have been too risk-averse. Assessing studies’ methodology (the index test) based on two questions is ultimately a simplification of an approach that may be hard to represent in two questions. We considered that the number of features should be proportional to the sample size, as more features than samples are known to cause overfitting. This is known as the Hughes phenomenon or the curse of dimensionality [18, 19].

We considered training on a set and testing on a hold-out set from the same site to be a high risk of bias because it allows manipulation of the results. There is no transparency in the split and there is no information on whether the performance will hold up if different distributions of the set are used for training and testing. This limitation likely has a small effect on the evidence as only ten studies were flagged as high risk because of this, which after sensitivity analysis did not show significant effects in the synthesis outcomes of any of the analyses.

A high risk of bias in patient selection indicated that the reported performance might be too specific to the selected sample. If the index test was judged at a high risk of bias, it indicated that the methodology was prone to overfitting. Both the former and the latter domains indicated a risk in the generalization ability of the experiment’s methodology. As all but two studies scored unclear in at least one of these domains, the main limitation in the evidence is the uncertainty [73, 117].

This uncertainty was mainly caused by what the authors explicitly reported. In our review, most studies used ABIDE. Only a reference to ABIDE is insufficient to determine whether inappropriate exclusions were made, comorbidity was avoided, and whether the sample was continuous or random. Even if reproducible selection criteria are reported, public databases may be updated, which invalidates the reproducibility. We consulted two other systematic reviews that also used the QUADAS-2 tool [15, 175]. They also reported the vast majority of studies were at high or uncertain risk of bias in the domain of patient selection. Therefore, we urge authors to describe the samples they used for their experiments in the study. This description should include the distribution of patients and healthy controls and their sex, age, and other potentially applicable information like the subject’s intelligence quotient. Information on how the sample was recruited should also be provided: if it was continuous or random and how selections were made [17]. A reference to the dataset should not replace this information.

The main obstacles in the way of clinical application are uncertainty and heterogeneity. While the former may be solved by making authors aware of what should be reported, the latter might also benefit from clear communication about specifications. Efforts have been made to harmonize the data obtained from different sites, in an attempt to minimize the imaging-protocol-induced heterogeneity [176]. Another idea is to not place a diagnostic tool in a universal framework altogether but to avoid cross-site heterogeneity by developing locally applied diagnostic tools [175, 177]. With these ideas in mind, we recommend that authors start reporting the imaging parameters on which their system works and the sample used together with its demographic information. Then, these studies can be assessed in a review which may outline consistent findings. The combined results may allow the creation of different protocols that each encompass part of the heterogeneity rather than a universal protocol that attempts to solve the heterogeneity altogether.

If heterogeneity’s impact and uncertainty can be sufficiently reduced, the results look promising. Sensitivity and specificity of 80% are proposed as the minimum diagnostic performance required for clinical application [175, 178]. The main summary estimate of 76.0% sensitivity and 75.7% specificity is not far off the clinical minimum, especially considering the captured heterogeneity has an adverse effect on diagnostic performance. The results of the synthesis on single-site experiments may be more appropriate to compare to the clinical guidelines, as no small-study effects were found, and the overall degree captured heterogeneity was lower. These results are very near the level of clinical application: 79.1% sensitivity and 80.0% specificity.

While critical assessment of new tools is necessary, we should not forget that the current diagnostic practice is not perfect. A systematic review, in which the performance of observation-based diagnostic methods was compared to the assessment of a multi-disciplinary team (considered the gold standard), concluded that any tool that correctly classifies ASD with 80% accuracy or more could be considered as accurate as the gold standard [179]. This underscores the need for and importance of an objective diagnosis based on a measurement. Despite the limitations, the results obtained in this review approach the level of the gold standard.

Current efforts of creating an objective diagnostic tool based on a measurement are usually trained and validated with ground-truth diagnoses obtained with methods prone to subjectivity, e.g., following the DSM-5 guidelines. All studies in the review provided a reference to an online dataset (e.g., ABIDE), or information on the used reference standard and their selection criteria, hence all studies scored a low risk of bias in the domain of the reference standard. These studies are all built upon a selection process that is more rigorous in distinguishing between individuals with ASD and healthy controls than diagnostics that are likely routinely practiced. For example, some studies mentioned using the Autism Diagnostic Observation Schedule-Generic or the Autism Diagnostic Interview-Revised, which are rigorous methods but often too time-consuming to be routinely performed [180, 181].

Based on the additional attention provided to the selection of individuals for the samples used in the reviewed studies, we think the effect of subjectivity on the ground-truth labels used in these studies is minimized. We also see this as an opportunity for MRI as an objective tool to translate this rigorous selection effort to practice in a time-efficient way, as it is trained and validated on these carefully selected data. Nevertheless, traces of the subjective nature of the current ground-truth data cannot be excluded completely from methods trained or validated on them. Therefore, an additional method of validation may be explored in the future. An objective diagnosis progressing from one measurement (e.g., MRI) could be used to validate an objective diagnosis progressing from a different measurement (e.g., blood-based) and vice versa. In this way, MRI and potentially other candidate objective tests could complement each other, and consensus between them may further add confidence to the true diagnosis of the tested individual. An approach like this would be largely in line with the NIMH’s Research Domain Criteria initiative, which suggests a framework that accommodates different objective measurements to complement the current psychopathological approach [182].

The diagnosis of ASD using MRI shows promise as consistent findings indicate that biomarkers capable of differentiating individuals with ASD and healthy controls exist and MRI is able to capture them. The main obstacles in the way of clinical application are heterogeneity and uncertainty about generalization. The state of the art in diagnosing ASD using MRI is best described as potent and desired, but not ready for clinical application yet.

### Supplementary information

Supplementary Materials A

Supplementary Materials B

Supplementary Materials B1

Supplementary Materials B2

Supplementary Materials C

PRISMA 2020 Checklist

## Data Availability

Data collection forms and data extracted from included studies are publicly available in the registry and in supplementary materials. All data used for analyses came from the extracted data. The scripts central to the conclusions in this review are available in the registry: https://osf.io/z3aeu. Furthermore, the most represented datasets in the review are publicly available [163, 164].
