# Supplementary material for: The diagnosis of ASD with MRI: a systematic review and meta-analysis
Source: Transl Psychiatry. 2024 Aug 2;14:318. doi: 10.1038/s41398-024-03024-5 (PMC11297045; doi:10.1038/s41398-024-03024-5)
Supplement: Supplementary file 1 — Supplementary Materials A [file 41398_2024_3024_MOESM1_ESM.docx]

**Abbreviations:**

ASD: Autism Spectrum Disorder
{d, f, s}MRI: {diffusion, functional, structural} Magnetic Resonance Imaging
HC : healthy control
SVM: Support Vector Machine
{A}NN: {Artificial} Neural Network
DSM: Diagnostic and Statistical Manual of Mental Disorders
ICD: International Classification of Diseases
ADOS: Autism Diagnostic Observation Schedule
ADI(-R): Autism Diagnostic Interview (-Revised)
AI: artificial intelligence
IQ: intelligence quotient
QUADAS: Quality Assessment of Diagnostic Accuracy Studies

**A1: Search strategy:**

**Criteria**

Inclusion:

1. Studies in which the participants have a clinical diagnosis of ASD without record of other mental illness. Accepted clinical diagnoses are based on, but not limited to, the DSM criteria, ICD, ADOS, ADI.
2. Studies that mention where or how they got all of their data and provide information on the sample size and class distribution of ASD and controls used in the reported experiments.
3. Studies that use dMRI, resting-state fMRI, or sMRI, or a combination thereof.
4. Studies that quantify features from MRI (can be automatic with AI) to classify ASD and HC (phenotypic features are allowed to be included).
5. Studies that explicitly express results including at least classification accuracy, sensitivity, and specificity or if the information required to calculate the latter metrics is reported.
6. Published in a journal or proceedings papers between 2018-01-01 and 2022-12-31 (YY-MM-DD).
7. Participants do not have a known clinical diagnosis of any other neuropsychiatric disorder, i.e. no confirmed comorbid mental illness.
8. Peer reviewed

Exclusion:

1. Articles not written in English
2. No reported experiments are carried out on subjects with a clinical diagnosis of ASD
3. Meta-analyses, surveys, editorial comments or reviews
4. Papers which do not match the search term according to the used search engines.
5. Studies that do not classify between ASD and HC.
6. Studies that used data from MRI obtained while patients are not in resting state (e.g. only experiments involving task-based fMRI).
7. Studies that look at ASD severity instead of diagnosis between ASD and HC.
8. Studies that only look at group differences instead of individual-level classification.
9. Studies that are not exact duplicates but predecessors of later versions with a lot of overlap in the content (e.g. conference papers that precede a journal publication).
10. Studies that only obtain results based on techniques (e.g. genetic analysis) or modalities other than dMRI, rsfMRI, or sMRI.
11. Studies that do not specify the required classification metrics in text or a table but only in graphs/figures; the numbers are not explicitly mentioned.
12. Studies that include longitudinal data (without a separate sample that does not include longitudinal data).
13. Studies that train and test on data obtained from the same patients, or if it is unclear if this occurs because of e.g. data augmentation or longitudinal data. (The test set should entirely consist of patients that are unseen to the classifier.)
14. Studies that use the same terms for performance metrics, but with other (or with a mistake in the) definitions which makes the comparison between metrics across studies invalid.
15. Studies that do not specify how they train/validate/test their classifiers/algorithms.

**Search term:**

(“Autism" OR "Asperger" OR "autism spectrum disorder" OR "ASD") AND ("classification" OR "machine learning" OR "SVM" OR "NN" OR "prediction" OR "deep learning" OR "computer aided diagnosis") AND ("magnetic resonance imaging" OR "MRI")

(all fields) (excluding reviews if the option is available) (between 2018-01-01 and 2022-12-31)

**Search Engines:**

Web of Science and PubMed

**If papers report multiple results:**

In the case of a paper reporting results of multiple experiments, the decision is made to only include the experiments carried out on different sets of data. The motivation behind this is that many different classifiers may be explored on which results may be reported. If all of these results are included, the weight of the paper in the analysis increases disproportionately while it is all based on the same dataset. If multiple experiments are made on the same dataset, the results outlined by the authors are chosen (e.g. stated in the discussion, conclusion or abstract). Otherwise the best results are chosen per dataset.

**Specification on data that needs to be reported to not be excluded:**


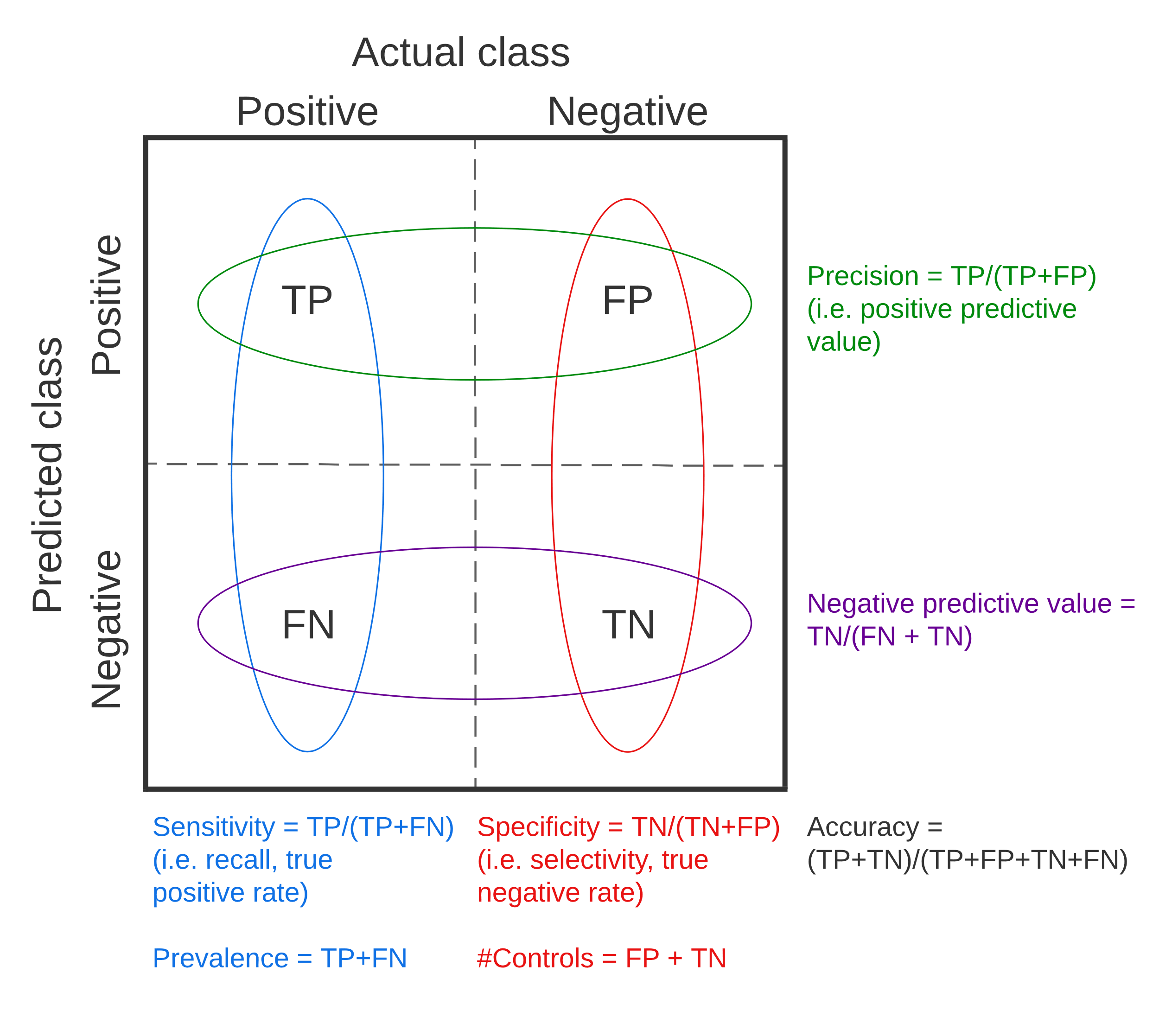


Figure 1: Confusion matrix of predicted and actual classes and the associated performance metrics (TP: true positives, FP: false positives, FN: false negatives, TN: true negatives)

The accuracy, sensitivity, and specificity are used in the statistical analysis. Therefore, these metrics need to be reported. If these metrics are not reported, but information is provided that allows these to be calculated, the study will not be excluded for this reason. Note that this information needs to be reported about the test set. Consult Figure 1 for an explanation of the metrics. In short, the number of true positives, true negatives, false positives, and false negatives should be derivable. Any of the points below are considered sufficient:

- If the number of true positives (TP), true negatives (TN), false positives (FP), and false negatives (FN) are reported.
- If the total number of individuals with ASD (NASD) and the number of healthy controls (NHC) are provided and at least the sensitivity (recall) and precision:

$$TP=NASD\cdot sensitivity$$

$$FN=NASD-TP$$

$$FP=TP\cdot\frac{1-precision}{precision}$$

$$TN=NHC-FP$$

- If the total number of individuals with ASD (NASD) and the number of healthy controls (NHC) are provided and at least the specificity and the negative predictive value (NPV):

$$TN=NHC\cdot specificity$$

$$FP=NHC-TN$$

$$FN= TN\cdot\frac{1-NPV}{NPV}$$

$$TP=NASD-FN$$

- If the number of individuals with ASD (NASD) and the number of healthy controls (NHC) are provided and at least the accuracy and sensitivity(recall):

$$TP=NASD\cdot Sensitivity$$

$$TN=\left( NASD+NHC \right)\cdot accuracy-TP$$

$$FP=NHC-TN$$

$$FN=NASD-TP$$

- If the number of individuals with ASD (NASD) and the number of healthy controls (NHC) are provided and at least the accuracy and specificity:

$$TN=NHC\cdot specificity$$

$$FP=NHC-TN$$

$$TP=\left( NASD+NHC \right)\cdot accuracy-TN$$

$$FN=NASD-TP$$

**A2: Measured variables (data extraction form)**

To report:

- Reference
- Publication Year
- Number of experiments in the study on different sets of data
- Per experiment the following will be reported:
  - Modalities (which types of MRI are used?)
  - Dataset
    - Name or in-house dataset (which dataset is used?)
    - Single site? (does the data come from one or more imaging sites?)
    - Which sites if multisite
    - Sample size (How many people in total?)
    - Number of individuals with ASD and healthy controls (Distribution of ASD and healthy controls)
    - Phenotypes
      - Age
        - Range ([youngest, oldest])
        - Mean
        - Standard deviation
      - Sex distribution (Number of males and females per group)
      - FIQ (Functional IQ)
  - Performance metrics:
    - Accuracy (how many predictions were made right?)
    - Sensitivity (how many out of the actual positive cases were predicted as such)
    - Specificity (how many of the actual negative cases were predicted as such)
  - Features
    - Atlas (Which atlas is used to divide the brain?)
    - Functional (if functional features are used they are described as below)
      - Static or dynamic if functional (how are the time series handled?)
      - Features (what is extracted from the time series?)
      - Processing (what method is used to extract features?)
        - Type of correlation (this is an example of a commonly used feature)

None (original values)

Normalized by Fisher transformation

Normalized by other methods

- - - Structural
      - Which morphological features?
      - Processing
    - Diffusion
      - Features
      - processing
    - Feature selection (Which algorithms are used to select features if any)
    - Phenotypes included? (Is meta data like age and sex included, if so which?)
    - AI extracted (Are the features extracted by AI (Artificial Intelligence)?)
    - Where possible, the number of features (how many features describe a subject?)
  - Classifier
    - Type (Which algorithm?)
    - Validation (How is training and testing managed?)
    - Ensemble? (Are multiple classifiers used together?)

In the case of a paper reporting results of multiple experiments, the decision is made to only include the experiments carried out on different sets of the data. The motivation behind this is that many different classifiers may be explored on which results are reported and if all of these results are included the weight of the paper in the analysis increases disproportionately. If multiple experiments are made on the same dataset, the results outlined by the authors are chosen (e.g. stated in the discussion, conclusion or abstract). Otherwise the best results are chosen per dataset.

**A3: QUADAS-2 (Study Risk of Bias and Quality Assessment)**:

(An overview of the changes is given below)

**Phase 1:**

Patients: individuals with a clinical diagnosis of autism spectrum disorder (ASD) (referred to as patients) without record of any other form of mental illness. The patients have underwent an MRI scan in resting state. Also healthy controls are included (no record of mental illness) who also underwent an MRI scan in resting state. The patients may have their MRI scan before or after their clinical diagnosis. The patients are included in this research through the papers that performed experiments on their MRI data.

The index test is the analysis of data obtained with an MRI scan.

The reference standard is a diagnosis based on clinical observation. This can be following the guidelines of e.g. the DSM-5, ICD-11, ADOS. The target condition is ASD.

**Phase 2:**

A flow diagram of the studies is provided in the paper.

**Phase 3:**

The questions in the following domains will be answered for each included study to assess the risk of bias and applicability.

**Domain 1: Patient selection**

- Risk of Bias:
  - Describe methods of patient selection
  - Questions (yes/no/unclear)
    - Was a consecutive or random sample of patients enrolled?
    - Did the study avoid inappropriate exclusions?
    - Did the study avoid potential comorbid cases?
    - Could the selection of patients have introduced bias? (low/high/unclear)
- Concerns regarding applicability
  - Describe sample characteristics and criteria
  - Level of concern about patients not matching the review question. (low/high/unclear)

**Domain 2: Index test(s)**

- Risk of Bias:
  - Describe the index test and how it was conducted and interpreted
  - Questions (yes/no/unclear)
    - Was more quality assurance than just a single same-site train/validation/test split used to avoid overfitting?
    - Was the number of features smaller than the number of participants?
    - Could the conduct or interpretation of the index test have introduced bias? (low/high/unclear)
- Concerns regarding applicability
  - Is there concern that the index test, its conduct, or interpretation differ from the review question? (low/high/unclear)

**Domain 3: Reference standard**

- Risk of Bias:
  - Describe the reference standard and how it was conducted and interpreted.
  - Questions (yes/no/unclear)
    - Is the reference standard likely to correctly classify the target condition?
    - Were the reference standard results interpreted without knowledge of the index test?
    - Could the reference standard, its conduct, or its interpretation have introduced bias? (low/high/unclear)
- Concerns regarding applicability
  - Is there concern that the target condition as defined by the reference standard does not match the review question? (low/high/unclear)

**Domain 4: Flow and timing**

Risk of Bias

- Describe the time interval, any interventions in between index test(s) and reference standard and excluded patients.
- Questions (yes/no/unclear)
  - Did all patients receive a reference standard?
  - Did patients receive the same reference standard?
  - Were all patients included in the analysis?
  - Could the patient flow have introduced bias? (low/high/unclear)

**Changes with respect to previous version and rationale**

As the previous version of the QUADAS2-tool had questions that proved to be redundant or contradictory in hindsight, we made the following changes.

Removed from Patient selection:

- Was a case-control design avoided?
  - As all included studies use ground-truth data to evaluate their models, this is never avoided. A case-control design is inherently present in supervised machine learning.
- Did the study match the patients and healthy controls by sex?
- Did the study match the patients and healthy controls by age?
  - The two questions above relate to matching the disease and control group. When evaluating diagnostic performance, it is desirable to not make any exclusions, which is likely necessary to match the groups. When trying to find a biomarker, it is desired to avoid any potential heterogeneity, such that any potential difference found to tell the groups apart can be confidently attributed to a clinically relevant feature (i.e. a biomarker). Therefore, matched groups are desired.
    Since a biomarker is not currently known, studies are explorative and most subliminally try to find a biomarker while also evaluating it as a diagnostic test. This is why the questions were initially included, but in hindsight we think it is better to focus on the main goal of the review which focuses on the assessment of a diagnostic test. Since these questions contradict the question about unnecessary exclusions, they are removed. This also simplifies the summarizing judgement over whole patient-selection domain.

Removed from Index test(s) :

- Were the index test results interpreted without knowledge of the results of the reference standard?
  - As diagnosing ASD using MRI is still in an explorative stage, no reference standard considers an MRI scan. This question is redundant in that sense and all studies received a yes here. We removed this question to not let a redundant question influence the summarizing judgment.

Removed from Flow and timing:

- Was there an appropriate interval between index test(s) and reference standard?
  - We see no reason to expect that any interval would not be appropriate, as the MRI scan does not influence the observation-based diagnosis and vice versa. No studies explained any details about this, which is why we deem this question redundant. We took it out so that it does not influence the summarizing judgment.

**Further rationale behind questions**

Rationale for the question on the index test ‘Was the number of features smaller than the number of participants?’: in short, the risk of overfitting. Machine learning models that train on features divide the feature space into regions that they are trained to attach a label (a class) to. It is a natural phenomenon of sampling to have blind spots in the feature space. A blind spot is the space between the features of seen observations (in this paper, individuals with ASD and controls). The machine learning algorithm usually learns to divide this blind spot into regions corresponding to desired classes based on what it is trained to learn. It is possible that the learned division is correct and no problems occur, but often, as a model is tested on more data, observations spill over and wrong classifications occur more frequently.

When fewer observations are present than the number of features, these blind spots increase as they are mapped to higher-dimensional space without sufficient observations to fill and reduce the blind spots. When a model is trained on more features than it has observations to learn from, these blind spots may result in overfitting. For reference, Berisha et al. (2021) wrote their comprehensive perspective on the matter [1].

It is hard to determine a threshold that accurately tells when the number of features is too high, as the possibility exists that these blind spots are filled in correctly. In absence of a way to determine this, we made the decision to draw the line at the number of observations (i.e. the sample size). While this may be conservative, which might result in being too risk averse, it does give some leeway as the whole dataset is taken rather than only the training set.

**A4: Included studies per year of publication and modality
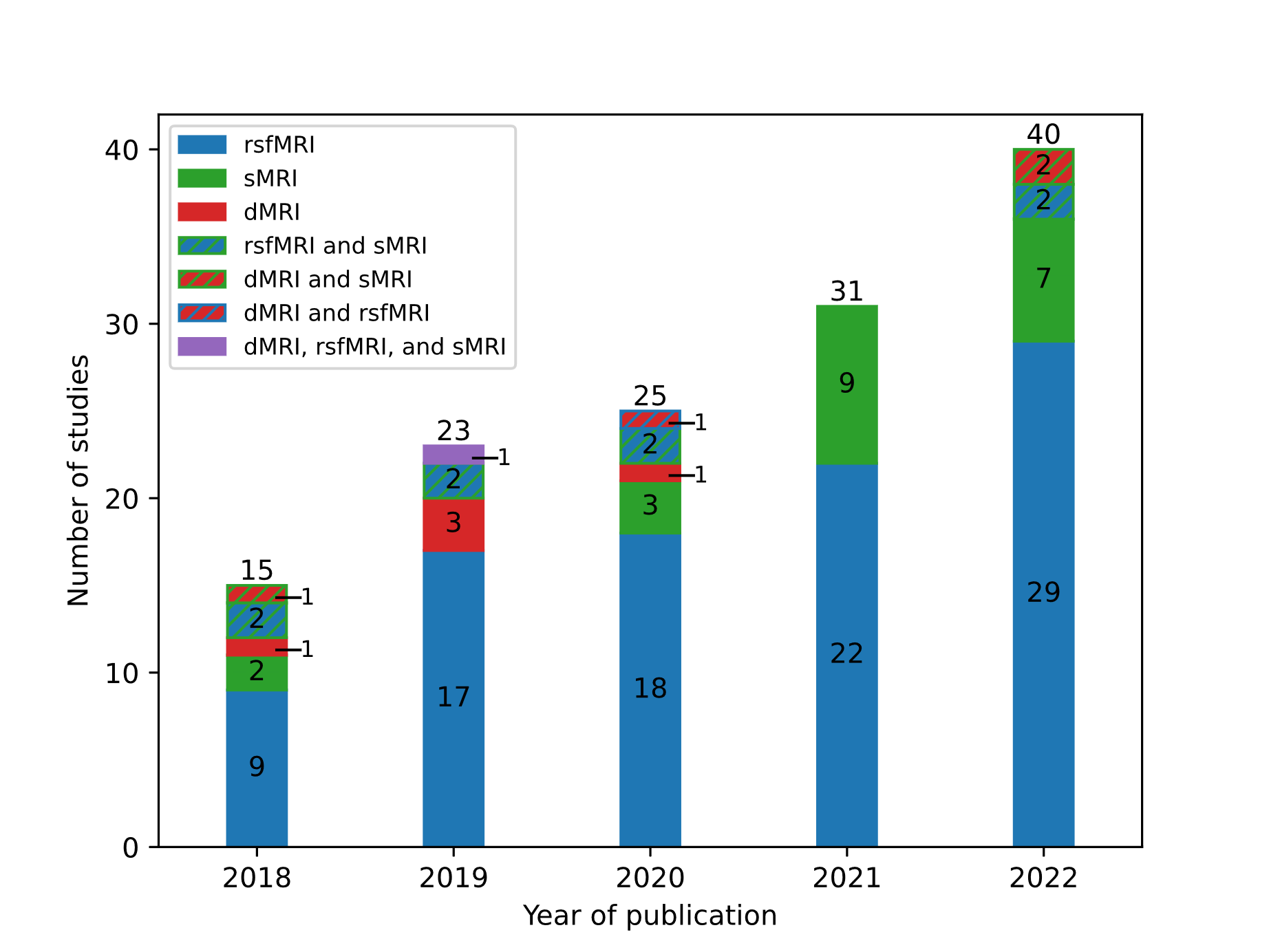
**

Figure 2: The number of included studies per year, where the studies’ modalities are color coded

**A5: Distribution of datasets used for experiments**

**
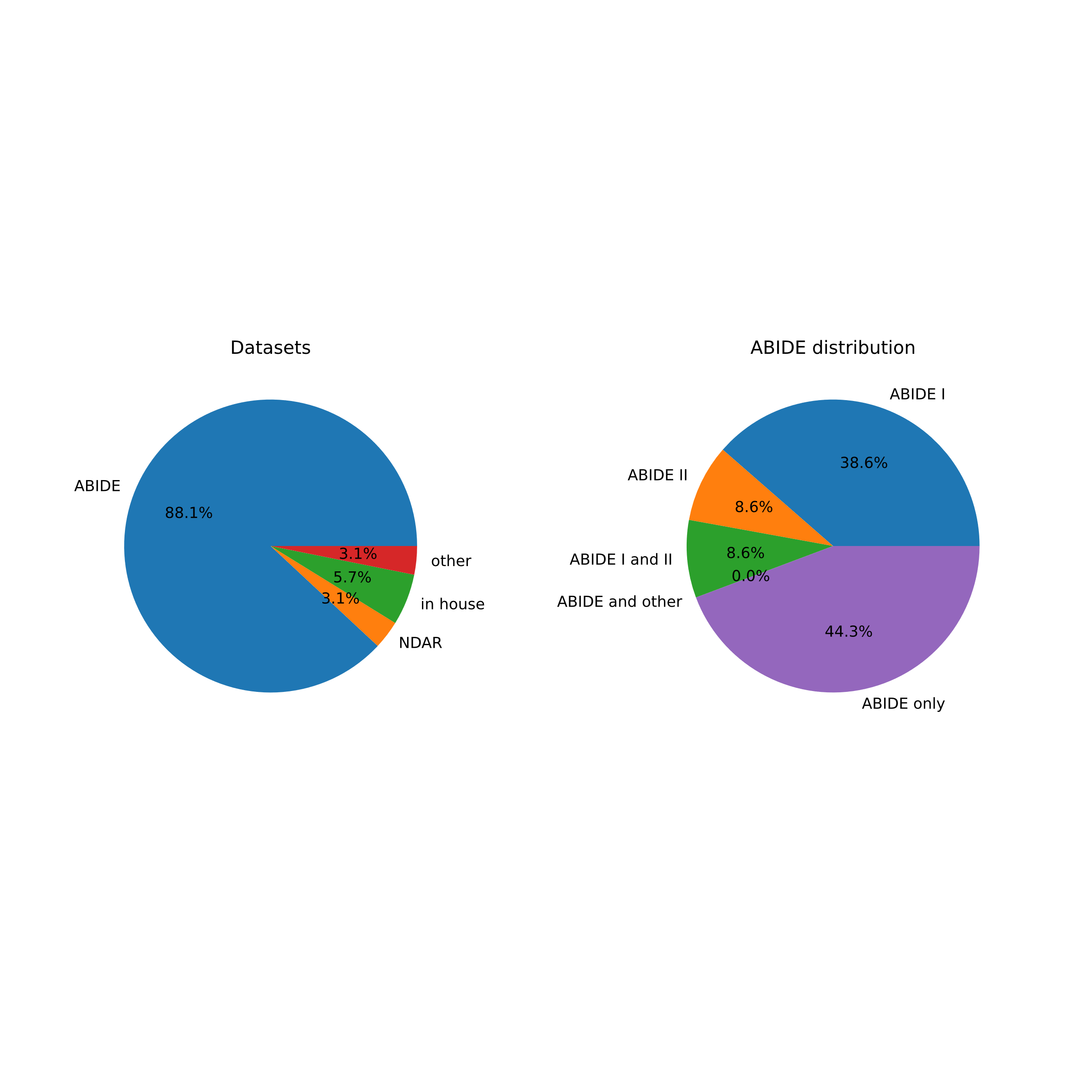
**

Figure 3: distribution of reported datasets (left) and the distribution over studies that reported ABIDE (right)

**A6: Modality distribution over included experiments**


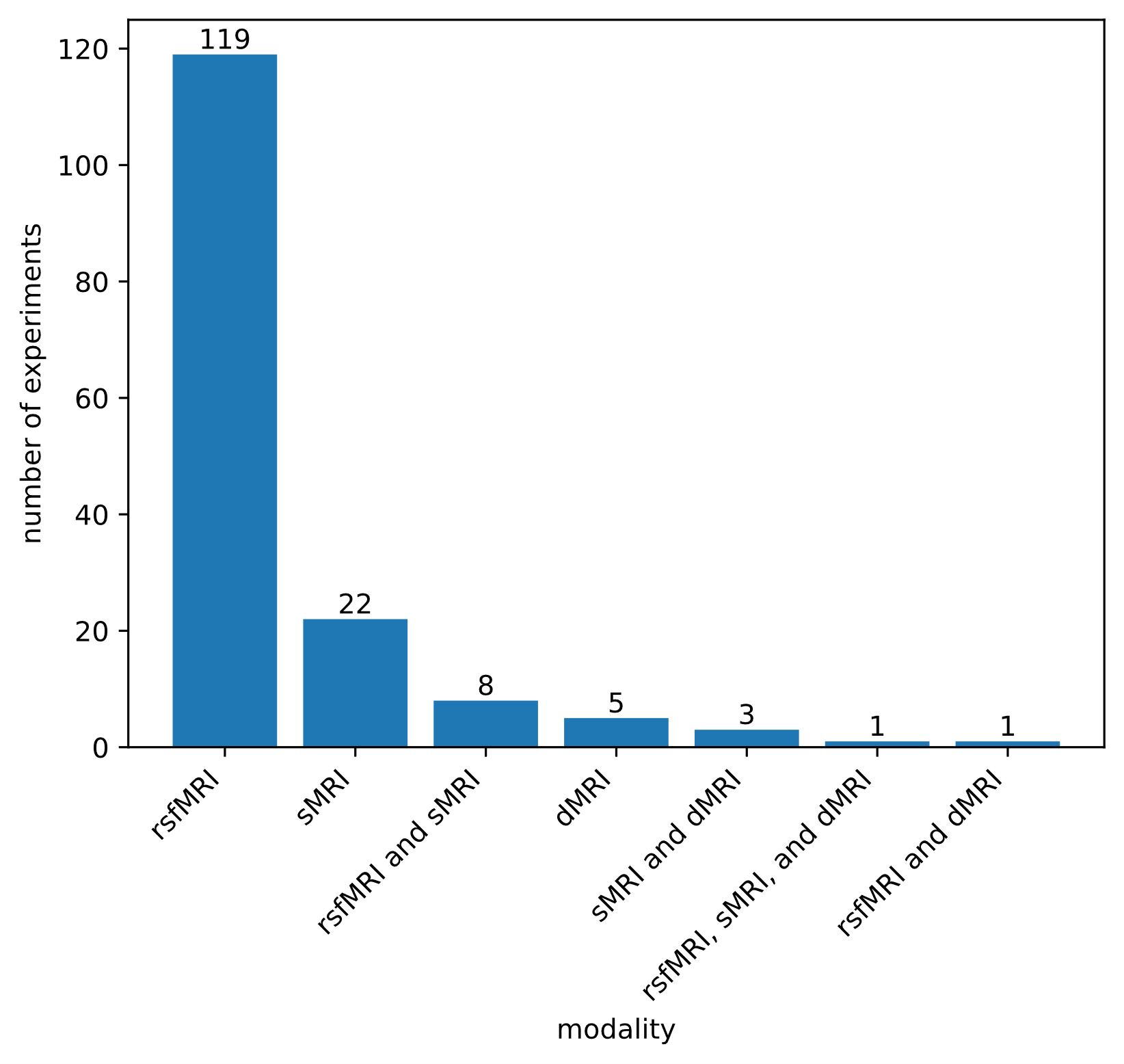


Figure 4: distribution of the modalities used in experiments (some studies reported more than one experiment)

**A7: sensitivity analysis**

| **Group** | **Sensitivity (CI)** | **Specificity (CI)** | **AUC** | **DOR (CI)** | $\boldsymbol{I}^{\boldsymbol{2}}$[%] |
| --- | --- | --- | --- | --- | --- |
| All | 0.762 (0.743-0.781) | 0.756 (0.738-0.774) | 0.824 | 9.84 (8.63-11.23) | 43.3 |
| rsfMRI | 0.749 (0.728-0.770) | 0.749 (0.730-0.767) | 0.812 | 8.86 (7.71-10.17) | 44.64 |
| sMRI | 0.808 (0.762-0.847) | 0.807 (0.737-0.861) | 0.869 | 18.96 (10.86-33.09) | 8.85 |
| dMRI | 0.646 (0.348-0.862) | 0.795 (0.613-0.905) | 0.800 | 8.06 (5.17-12.56) | 0 |
| multimodal | 0.842 (0.751-0.904) | 0.777 (0.676-0.854) | 0.878 | 17.64 (8.74-35.64) | 48.9 |
| Single-site | 0.791 (0.758-0.821) | 0.794 (0.761-0.824) | 0.860 | 15.84 (11.95-21.00) | 16.9 |
| Multi-site | 0.750 (0.727-0.773) | 0.743 (0.721-0.764) | 0.810 | 8.56 (7.38-9.93) | 48.8 |

The table above shows the result after repeating the primary analyses on the experiments remaining after exclusion of studies at high risk of bias.

**A8: Test for publication bias using Deek’s test**

**
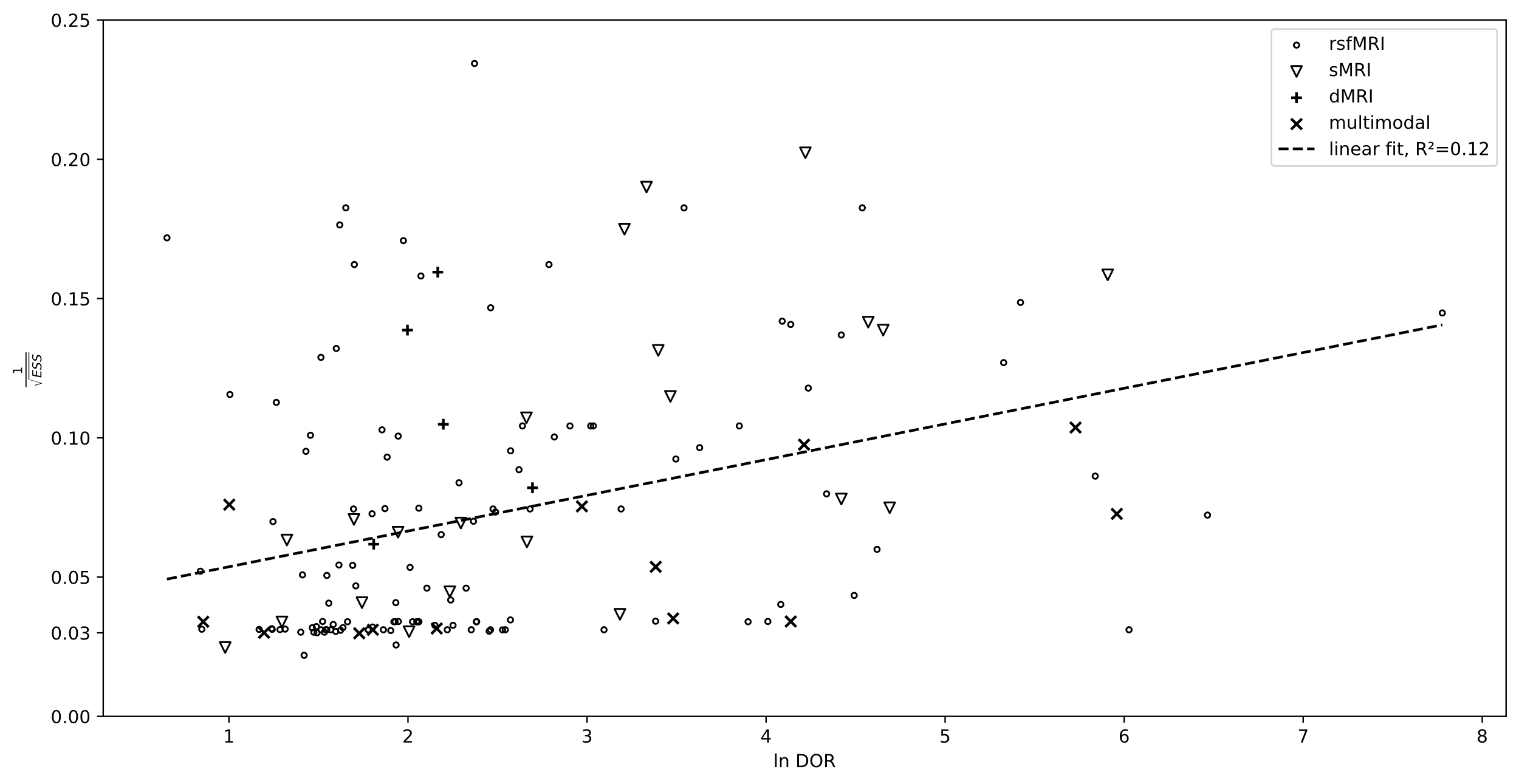
**

Figure 5: A quantity inversely proportional to sample size (one over the square root of the effective sample size) as a function of the natural logarithm of the diagnostic odds ratio. The experiments are scattered with a shape representing the used modality

One over the square root of the effective sample size (ESS), $ESS= \frac{4\cdot NASD\cdot NHC}{NASD+NHC}$, is plotted as a function of the natural logarithm of the diagnostic odds ratio (DOR) in the figure above. A linear fit is found by performing regression: $y=0.013x+0.041$, P=9.09e-06, standard error of 0.0028. The $R^{2}$ measure shows the proportion of the variance in the observations that can be explained by the fitted model.

The P-value shows a statistically significant correlation between $1/\surd ESS$ and ln DOR (i.e. a non-zero slope), which indicates a negative correlation between DOR and sample size. In other words: as sample size increases, diagnostic accuracy decreases, which is indeed the case as shown below.


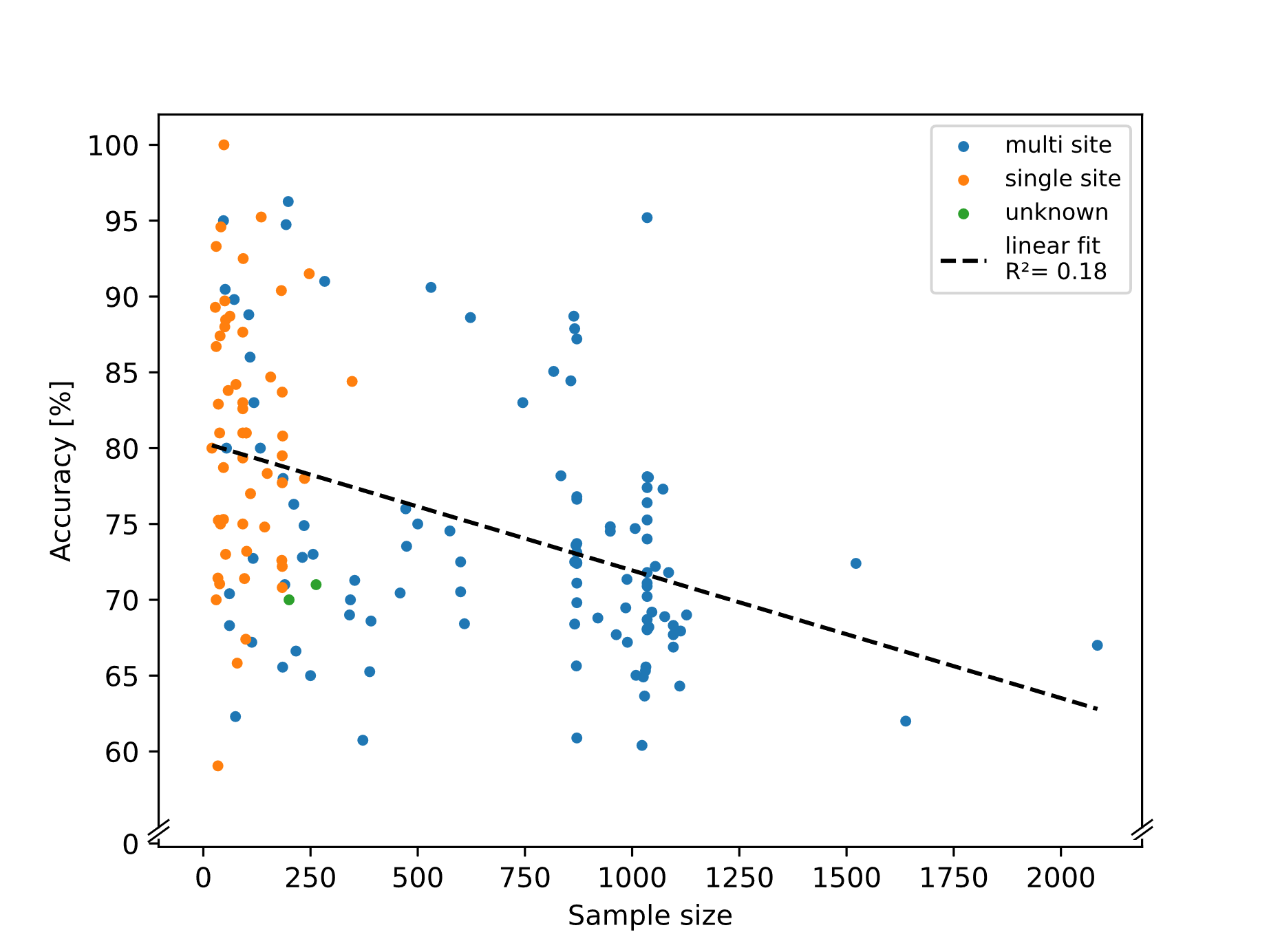


Figure 6: Diagnostic accuracy as a function of sample size

The accuracy as a function of sample size and a linear regression line $y=-0.008x+80.359$ P=3.81e-08.

Deek’s test for the subgroups:

| Group | Slope | P-value | Standard error |
| --- | --- | --- | --- |
| rsfMRI | 0.011 | **0.002** | 0.0034 |
| sMRI | 0.007 | **0.001** | 0.0069 |
| dMRI | -0.006 | 0.939 | 0.0700 |
| Multimodal | 0.009 | **0.034*** | 0.0039 |
| Single-site | 0.000 | 0.952 | 0.0043 |
| Multi-site | 0.009 | **<0.001** | 0.0023 |

Bold indicates statistical significance (P<0.05), * indicates significant before but not after Bonferroni correction

Note that if we correct Deek’s tests above (6 subgroups and the main test) for multiple comparisons using Bonferroni correction, the significance level becomes (P<0.0071). This would mean that the P-value for the main test and the bold P-values of the subgroup tests in the table above are still significant apart from the subgroup of multimodal studies (P=0.034).

**A9: Test for better performance of the Fisher transformation compared to raw Pearson’s correlation values**


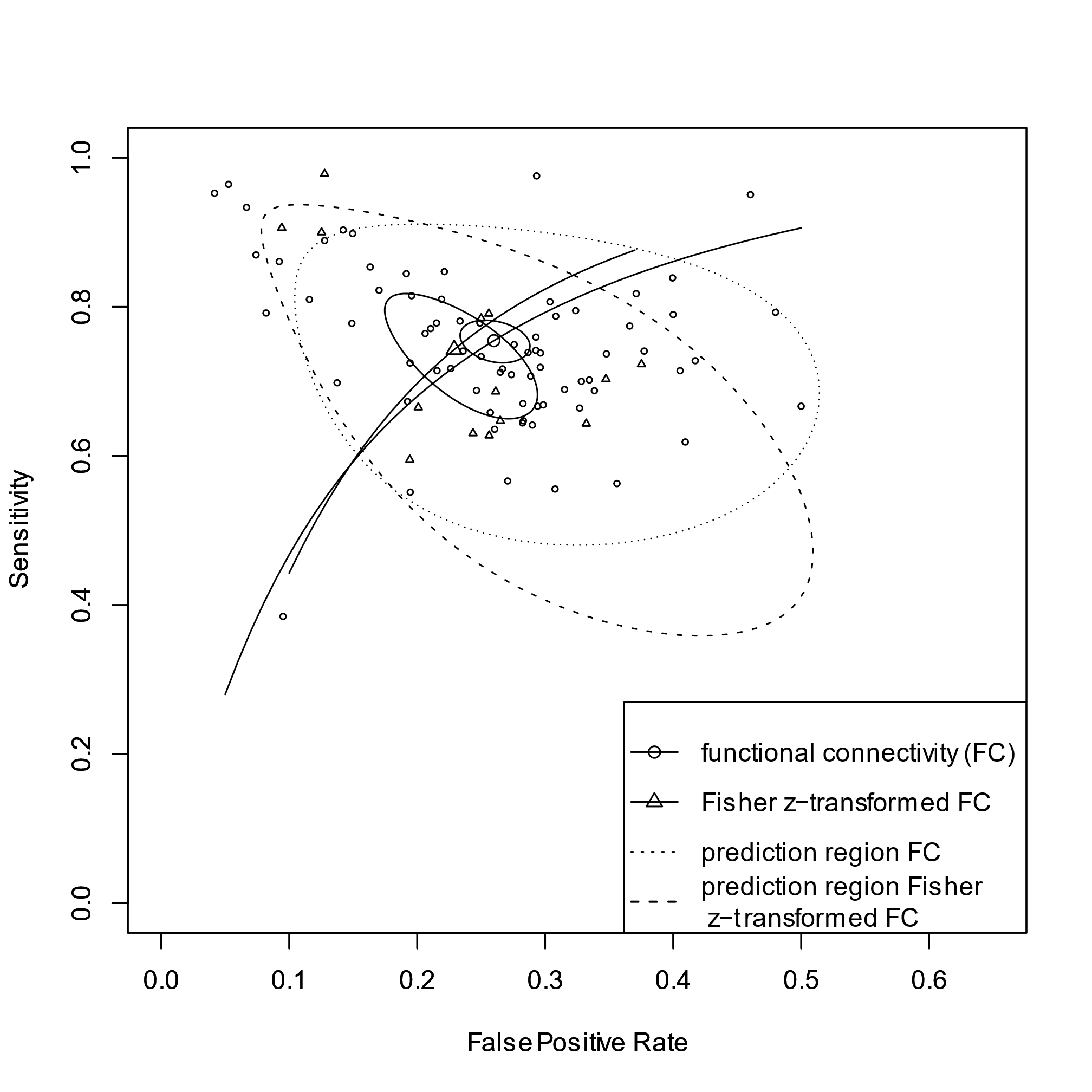


Figure 7: The summary receiver operator characteristics plots to compare the Fisher z-transform applied to functional connectivity to functional connectivity without the Fisher z-transform. The continuous-line ellipsoids surrounding the point estimates are the respective 95% confidence regions.

As can be seen in Figure 7, the point estimate for Fisher z-transformed FC (let us call this group Fisher) is slightly higher than FC without the transform (and let us call this group FC). Judging from the overlap in confidence regions, this difference is likely not statistically significant. To test this, we compare the diagnostic odds ratio (DOR) of both groups. The motivation behind using the DOR rather than sensitivity and specificity separately, is that we consider the sensitivity and specificity to be codependent as moving the decision threshold of a classifier will affect both. Therefore, we deem a univariate approach that takes both into account more suitable. The DOR can be expressed in terms of sensitivity and specificity through

$$DOR=\frac{sensitivity\times specificity}{\left( 1-sensitivity \right)\times(1-specificity)}.$$

We test for normality using the Shapiro-Wilk test, which showed the Fisher group (P=4.29e-06) and FC group (P=9.32e-17) to be not normally distributed. We tested for homogeneity of variances using Levene’s test (P=0.6934, F=0.1565), so we cannot reject homogeneity of variances. As the data are not normally distributed, we tested the group differences using the Wilcoxon rank-sum test (Mann-Whitney U test) with an alternative hypothesis that the Fisher group scores higher in terms of DOR. This Wilcoxon rank-sum test resulted in P=0.593 (W=498), so we cannot reject the null hypothesis. It should be noted that this also does not prove the opposite. It means that based on the data we collected from the studies, we do not see a significantly better performance when studies use the Fisher z-transform.

**References**

[1] Berisha, V. et al. Digital medicine and the curse of dimensionality. *NPJ digital medicine* **4**, 153 (2021).

[2] Sterne, J. A., Gavaghan, D. & Egger, M. Publication and related bias in meta-analysis: power of statistical tests and prevalence in the literature. *Journal of clinical epidemiology* **53**, 1119–1129 (2000).
