# Supplementary material for: The diagnosis of ASD with MRI: a systematic review and meta-analysis
Source: Transl Psychiatry. 2024 Aug 2;14:318. doi: 10.1038/s41398-024-03024-5 (PMC11297045; doi:10.1038/s41398-024-03024-5)
Supplement: Supplementary file 3 — Supplementary Materials B1 [file 41398_2024_3024_MOESM3_ESM.pdf]

The Diagnosis of ASD with MRI: a Systematic Review and Meta-analysis

Quality Assessment of Diagnostic Accuracy Studies 2 - QUADAS-2

QUADAS-2 reference: Whiting PF, Rutjes AW, Westwood ME, Mallett S, Deeks JJ, Reitsma JB, Leeflang MM, Sterne JA, Bossuyt PM; QUADAS-2 Group. QUADAS-2: a revised tool for the quality assessment of diagnostic accuracy studies. Ann Intern Med. 2011 Oct 18;155(8):529-36. doi: 10.7326/0003-4819-155-8-201110180-00009. PMID: 22007046.

CRA: concerns regarding applicability

	**Patient selection**							**Index test**					**Reference standard**					**Flow and timing**				
	Risk of Bias					CRA		Risk of Bias				CRA	Risk of Bias				CRA	Risk of Bias				
Reference	Description	Was a consecutive or random sample of patients enrolled?	Did the study avoid inappropriate exclusions?	Did the study avoid potential comorbid cases?	Could the selection of patients have introduced bias?	Describe the sample characteristics and criteria.	Level of concern about patients not matching the review question.	Description	Was more validation than just a same-site train/validation/test split used to avoid overfitting?	Was the number of features smaller than the number of participants?	Could the conduct or interpretation of the index test have introduced bias?	Level of concern about the index test, its conduct, or interpretation differing from the review question.	Description	Is the reference standard likely to correctly classify the target condition?	Were the reference standard results interpreted without knowledge of the index test?	Could the reference standard, its conduct, or its interpretation have introduced bias?	Is there concern that the target condition as defined by the reference standard does not match the review question?	Description	Did all patients receive a reference standard?	Did patients receive the same reference standard?	Were all patients included in the analysis	Could the patient flow have introduced bias?
**Aghdam, M. et al., 2018**	From ABIDE I&II, patients between 5 and 10 years of age from sites acquiring between 160 and 180 time points were	Unclear	No	Unclear	Unclear	Data was obtained from ABIDE I & II. Sites from ABIDE I: NYU, Stanford, SDSU. Sites from ABIDE II: EMC, SDSU, NYU1,	Low	The functional features are statically obtained from ROIs specified by the AAL atlas. The time series per ROI were	Yes	No	Unclear	Low	No information on the reference standard was provided in this paper explicitly, however a reference to ABIDE I	Yes	Yes	Low	Low	No information on the time interval or interventions has been provided, but a reference to ABIDE I & II is provided. All	Yes	Unclear	Yes	Low
**Aghdam, M. et al., 2019**	From ABIDE I&II, patients between 5 and 10 years of age were selected.	Unclear	Unclear	Unclear	Unclear	Data was obtained from ABIDE I & II. Sites from ABIDE I: Pitt, Olin, OHSU, SDSU, USM, KKI, NYU. Stanford. UCLA. Sites	Low	Experiment 1 : Features were extracted using AI. A CNN was used to extract features from time concatenated slices	Yes	Unclear	Unclear	Low	Experiment 1 : No information on the reference standard was provided in this paper explicitly, however a reference to ABIDE I	Yes	Yes	Low	Low	Experiment 1 : No information on the time interval or interventions has been provided. but a reference to	Yes	Unclear	Yes	Low
**Ahammed, S. et al., 2021**	From the selected sites all patients were selected	Unclear	Yes	Unclear	Unclear	Data was obtained from ABIDE I. The site caltech is selected. A total of 38 participants were included of which 19 with ASD	Low	Experiment 1 : The functional features are statically obtained from ROIs specified by no reported atlas. The SURF	No	No	High	Low	Experiment 1 : No information on the reference standard was provided in this paper explicitly, however a reference to ABIDE I	Yes	Yes	Low	Low	Experiment 1 : No information on the time interval or interventions has been provided. but a reference to	Yes	Unclear	Yes	Low
**Ahmed, R. et al., 2020**	Patients are taken from the site NYU of ABIDE (I or II is not specified), there is no information on selection	Unclear	Yes	Unclear	Unclear	Data was obtained from ABIDE. The site NYU is selected. A total of 184 participants were included of	Low	Features were extracted using AI. A Restricted Boltzmann Machine was used to extract features from preprocessed	Yes	Unclear	Unclear	Low	No information on the reference standard was provided in this paper explicitly, however a reference to ABIDE	Yes	Yes	Low	Low	No information on the time interval or interventions has been provided, but a reference to ABIDE is provided. All	Yes	Unclear	Yes	Low
**Al-Hiyali, M. et al., 2021**	As an experiment on multi-class classification was also performed, patients are selected according to the DSM-	Unclear	No	Unclear	Unclear	Data was obtained from ABIDE. The sites NYU, SBL, SDSU, Yale are selected. A total of 72 participants were	Low	The functional features are dynamically obtained from ROIs specified by the AAL atlas. The wavelet coherence of	Yes	No	Unclear	Low	No information on the reference standard was provided in this paper explicitly, however a reference to ABIDE	Yes	Yes	Low	Low	No information on the time interval or interventions has been provided, but a reference to ABIDE is provided. All	Yes	Yes	Yes	Low
**Al-Hiyali, M. et al., 2022**	The used dataset is ABIDE. No selection criteria within ABIDE.	Unclear	Yes	Unclear	Unclear	Data was obtained from ABIDE. The sites SDSU, Stanford, UM, KKI, KUL1, KUL2, SBL. TCD. caltech. CMU.	Low	The functional features are obtained with the AAL atlas. The principal wavelet component maps were	Yes	Unclear	Unclear	Low	No information on the reference standard was provided in this paper explicitly, however a reference to ABIDE	Yes	Yes	Low	Low	No information on the time interval or interventions has been provided, but a reference to ABIDE is provided. All	Yes	Unclear	Yes	Low
**Bhaumik, R. et al., 2018**	Patients were selected from ABIDE. The paper mentions demographics of the individuals satisfying inclusion criteria. but	Unclear	Unclear	Yes	Unclear	Data was obtained from ABIDE. The sites NYU, Stanford, Olin, KKI, SDSU, Pitt are selected. A total of 372	Low	The functional features are statically obtained from ROIs specified by the Brodmann atlas. The functional	Yes	Unclear	Unclear	Low	No information on the reference standard was provided in this paper explicitly, however a reference to ABIDE	Yes	Yes	Low	Low	No information on the time interval or interventions has been provided, but a reference to ABIDE is provided. All	Yes	Unclear	Yes	Low
**Bi, X. et al., 2020**	A subset of individuals with Asperger is selected from ABIDE. There are no further selection criteria described. but	Unclear	Unclear	Unclear	Unclear	Data was obtained from ABIDE. The site selected is not specified. A total of 135 participants were included of	Low	The functional features are statically obtained from ROIs specified by the AAL atlas. The graph-based measures	No	No	High	Low	Individuals with Asperger are diagnosed with ADOS-G. As AS on the spectrum of ASD, there is no concern regarding	Yes	Yes	Low	Low	No information on the time interval or interventions has been provided, but a reference to ABIDE is provided. All	Yes	Yes	Yes	Low
**Bilgen, I. et al., 2020**	Patients were taken from ABIDE without further specification.	Unclear	Unclear	Unclear	Unclear	Data was obtained from ABIDE. The site is not specified. A total of 200 participants were included of which 100	Low	The structural features are connectional network of cortical morphology obtained with the Desikan-Killiany atlas	No	Unclear	High	Low	No information on the reference standard was provided in this paper explicitly, however a reference to ABIDE	Yes	Yes	Low	Low	No information on the time interval or interventions has been provided, but a reference to ABIDE is provided. No	Yes	Unclear	Unclear	Unclear
**Brahim, A. and Farrugia, N., 2020**	Patients were selected based on the quality of their scans: 'The selection of the data is based on the results of quality	Unclear	No	Unclear	Unclear	Data was obtained from 17 sites of ABIDE. A total of 871 participants were included of which 403 with ASD and 468	Low	The functional features are statically obtained from ROIs specified by the Glasser atlas. The standard deviation of fMRI	Yes	Yes	Low	Low	No information on the reference standard was provided in this paper explicitly, however a reference to ABIDE	Yes	Yes	Low	Low	No information on the time interval or interventions has been provided, but a reference to ABIDE is provided. All	Yes	Unclear	Yes	Low
**Byeon, K. et al., 2020**	Patients without available IQ levels were excluded. Patients whose scans had severe distortions were excluded.	Unclear	No	Unclear	Unclear	Data was obtained from ABIDE I. The sites caltech, CMU, NYU, SDSU, Stanford, TCD, UM1, UM2, USM, Yale are selected. A	Low	Features were extracted using AI. A averaging of time-series per ROI was used to extract features from fMRI time-	Yes	No	Unclear	Low	The reference standard that is reported is the diagnosis as per the ADOS score. This is provided for most but not all of	Yes	Yes	Low	Low	No information on the time interval or interventions has been provided, but a reference to ABIDE I is provided. All	Yes	Unclear	Yes	Low
**Bernas, A. et al., 2018**	Two independent samples were used in this study. The in-house sample consisted of 15 adolescents with ASD and 18	Unclear	No	Yes	Unclear	An in-house dataset and data from ABIDE are used. The site Leuven is selected. A total of 54 participants were included	Low	The functional features are dynamically obtained from ROIs specified by the Smith et al. (2006) atlas. The average	Yes	Unclear	Unclear	Low	All individuals with ASD have a diagnosis based on the DSM-IV_TR criteria.	Yes	Yes	Low	Low	No information on the time interval or interventions has been provided, but a reference to ABIDE is provided.	Yes	Yes	Yes	Low
**Cao, M. et al., 2021**	Participants were taken from ABIDE I, specifically 871 subjects from 17 sites. 'The data were screened based on	Unclear	No	No	High	Data was obtained from ABIDE I. The sites caltech, CMU, KKI, KUL, maxmul, NYU, OHSU, Olin, Pitt, SBL, SDSU, Stanford, TCD,	Low	The functional features are statically obtained from ROIs specified by the Harvard-Oxford atlas. The functional	Yes	No	Unclear	Low	No information on the reference standard was provided in this paper explicitly, however a reference to ABIDE I	Yes	Yes	Low	Low	No information on the time interval or interventions has been provided, but a reference to ABIDE I is provided. All	Yes	Unclear	Yes	Low
**Cao, P. et al., 2022**	It is specified that ABIDE comprises 1112 subjects of which 402 individuals with ASD and 464 healthy controls are	Unclear	No	Unclear	Unclear	Data was obtained from 17 sites of ABIDE. A total of 866 participants were included of which 402 with ASD and 464	Low	The functional features are dynamically obtained from ROIs specified by the AAL atlas. The functional connectivity	Yes	Unclear	Unclear	Low	No information on the reference standard was provided in this paper explicitly, however a reference to ABIDE	Yes	Yes	Low	Low	No information on the time interval or interventions has been provided, but a reference to ABIDE is provided. Patients	Yes	Unclear	Yes	Low
**Chen, T. et al., 2020**	Out of the 17 sites of ABIDE II, 4 are selected: ETH, NYU, OHSU, and SU. All participants were selected from the sites.	Unclear	No	Unclear	Unclear	Data was obtained from ABIDE II. The sites ETH, NYU, OHSU, Stanford are selected.A total of 250 participants were included	Low	The structural features are 3D HOG obtained with no reported atlas through image gradients per 3D cell specified by two	Yes	Unclear	Unclear	Low	No information on the reference standard was provided in this paper explicitly, however a reference to ABIDE	Yes	Yes	Low	Low	No information on the time interval or interventions has been provided, but a reference to ABIDE II is provided. All	Yes	Unclear	Yes	Low
**Chen, X. et al., 2022**	The dataset ABIDE is referenced, from which 500 individuals were selected: 250 with ASD and 250 healthy	Unclear	No	Unclear	Unclear	Data was obtained from ABIDE. The selected site(s) is/are not specified. A total of 500 participants were included	Low	The structural features are extracted from 3D scans obtained from the preprocessed sMRI-scan data.	Yes	Unclear	Unclear	Low	No information on the reference standard was provided in this paper explicitly, however a reference to ABIDE	Yes	Yes	Low	Low	No information on the time interval or interventions has been provided, but a reference to ABIDE is provided. Some	Yes	Unclear	Unclear	Unclear
**Chen, Y. et al., 2022**	Participants were taken from ABIDE I. Out of 1112 available subjects, 1007 subjects with both sMRI scan data and	Unclear	Unclear	Unclear	Unclear	Data was obtained from ABIDE I. The sites caltech, CMU, KKI, KUL, maxmul, NYU, OHSU, Olin, Pitt. SBL. SDSU. Stanford. TCD.	Low	The functional features are statically obtained from ROIs specified by the AAL atlas. The functional connectivity and	Yes	No	Unclear	Low	No information on the reference standard was provided in this paper explicitly, however a reference to ABIDE I	Yes	Yes	Low	Low	No information on the time interval or interventions has been provided, but a reference to ABIDE I is provided. All	Yes	Unclear	Yes	Low
**Chen, Zhihui et al., 2019**	From ABIDE I 440 individuals with ASD and 480 healthy controls are selected. There is no specification of the	Unclear	No	Unclear	Unclear	Data was obtained from ABIDE I. The site is not specified. A total of 920 participants were included of which 440 with ASD	Low	Features were extracted using AI. A sliding window, Pearson's correlation coefficient and Fisher's z-transform were used	Yes	Unclear	Unclear	Low	No information on the reference standard was provided in this paper explicitly, however a reference to ABIDE I	Yes	Yes	Low	Low	No information on the time interval or interventions has been provided, but a reference to ABIDE is provided. It is	Yes	Unclear	Unclear	Unclear
**Chu, Y. et al., 2022**	Patients were taken from the NYU site in the ABIDE database. No information on selection criteria is provided.	Unclear	Yes	Unclear	Unclear	Data was obtained from ABIDE. The site NYU is selected. A total of 184 participants were included of	Low	Features were extracted using AI. Pearson's correlation coefficient between mean time series of pairs ROIs was used to	Yes	Unclear	Unclear	Low	No information on the reference standard was provided in this paper explicitly, however a reference to ABIDE	Yes	Yes	Low	Low	No information on the time interval or interventions has been provided, but a reference to ABIDE is provided. All	Yes	Unclear	Yes	Low
**Dammu, P. and Bapi, R., 2019**	Participants were taken from ABIDE. Scans that have a duration of less than five minutes are exlcuded. The sites	Unclear	No	Unclear	Unclear	Data was obtained from ABIDE. The sites CMU, KKI, maxmul, NYU, Olin, SDSU, SBL, Stanford. UCLA. KUL. UM. USM.	Low	The functional features are dynamically obtained from ROIs specified by the AAL atlas. The average duration of	Yes	Yes	Low	Low	No information on the reference standard was provided in this paper explicitly, however a reference to ABIDE	Yes	Yes	Low	Low	No information on the time interval or interventions has been provided, but a reference to ABIDE is provided. All	Yes	Unclear	Yes	Low
**Dekhil, O. et al., 2018**	Participants were taken from NDAR. Participants were selected based on the availability of data from other	Unclear	No	Unclear	Unclear	Data was obtained from NDAR. The sites UCLA and WU are selected. A total of 283 participants were included of	Low	A probablistic individual component analysis was used to extract ROIs for which power spectral densitities were	Yes	Unclear	Unclear	Low	The diagnosis of ASD was based on the standard of clinical observation. ADOS and ADI-R are reported for 96 and	Yes	Yes	Low	Low	No information on the time interval or interventions has been provided, but a reference to NDAR is provided. All	Yes	No	Yes	Low
**Deng, X. et al., 2022**	From 16 and 17 sites of ABIDE I and II, separately. Selection: according to the length of their scan data.	Unclear	No	Unclear	Unclear	Data was obtained from ABIDE II. The sites BNI, EMC, ETH, GU, IU, KKI, KUL, NYU, OHSU, Olin, SDSU, Stanford, TCD, UCD,	Low	Experiment 1 : Features were extracted using AI. A time averaging was used to extract features from time series per	Yes	Unclear	Unclear	Low	Experiment 1 : No information on the reference standard was provided in this paper explicitly, however a reference to ABIDE I	Yes	Yes	Low	Low	Experiment 1 : No information on the time interval or interventions has been provided, but a reference to	Yes	Unclear	Yes	Low
**Denier, N. et al., 2021**	Patients were selected based on their age (> 20 years old). Only males were included	Unclear	No	Unclear	Unclear	Data was obtained from ABIDE I. The sites are unknown. A total of 256 participants were included of which 120 with ASD	Low	The structural features are head circumference, intracranial volume, total brain volume, GM volume, WM	No	Yes	High	Low	No information on the reference standard was provided in this paper explicitly, however a reference to ABIDE I	Yes	Yes	Low	Low	No information on the time interval or interventions has been provided, but a reference to ABIDE I is provided. All	Yes	Unclear	Yes	Low
**Dekhil, O. et al., 2019**	Patients were selected based on the availability of high-resolution T1-weighted MRI data. 7 subjects with corrupted	Unclear	No	Unclear	Unclear	Data was obtained from NDAR. The site George Washington University is selected. A total of 185 participants were	Low	The functional features are statically obtained with the Desikan-Killiany atlas. The functional connectivity	Yes	Yes	Low	Low	The reference standard is the ADOS report.	Yes	Yes	Low	Low	No record of any exclusions after initial selection. It is stated that some patients had ADOS reports as reference	Yes	Unclear	Yes	Low
**Duan, Y. et al., 2022**	Participants were selected from three sites of ABIDE II. Children between 7 and 15 years of age were selected.	Unclear	No	Unclear	Unclear	Data was obtained from ABIDE II. Sites from ABIDE II: GU, OHSU, UCLA. A total of 231 participants were included of	Low	The structural features are volumes of gray matter clusters obtained through a sliding spherical window and matched	Yes	Unclear	Unclear	Low	No information on the reference standard was provided in this paper explicitly, however a reference to ABIDE	Yes	Yes	Low	Low	No information on the time interval or interventions has been provided, but a reference to ABIDE II is provided. All	Yes	Unclear	Yes	Low
**Dvornek, C. et al., 2019**	Participants were selected from 4 sites of ABIDE I. No further selection criteria are described.	Unclear	Unclear	Unclear	Unclear	Data was obtained from ABIDE I. The sites NYU, UM, USM, and UCLA are selected. A total of 527 participants were included	Low	The index test was performed on 4 different sites. For each site the same approach was taken. Features were extracted	Yes	Unclear	Unclear	Low	No information on the reference standard was provided for each of the experiments in this paper	Yes	Yes	Low	Low	No information on the time interval or interventions has been provided for any of the participants, but a reference to	Yes	Unclear	Unclear	Unclear
**Eill, A. et al., 2019**	Participants were between 7 and 18 years of age. ASD participants had diagnoses verified with ADOS and ADI-R.	Unclear	No	Yes	Unclear	An in-house dataset from SDSU is used. A total of 93 participants were included of which 46 with ASD and 47	Low	The functional features are statically obtained with the Power atlas. Functional connectivity features were	Yes	Yes	Low	Low	ASD participants had diagnoses verified with ADOS and ADI-R	Yes	Yes	Low	Low	No time interval or any interventions between the index test and reference standard are described.	Yes	Yes	Yes	Low
**El-Gazzar, A. et al., 2019**	Participants were taken from ABIDE I&II. Participants were excluded if their scan duration was less than 100 time points.	Unclear	No	Unclear	Unclear	Data was obtained from ABIDE I and II. All sites are selected. A total of 2085 participants were included of which 993 with ASD	Low	Features were extracted using AI. Time averaging per ROI was used to extract the time series used as input to AI. The number	Yes	Unclear	Unclear	Low	No information on the reference standard was provided in this paper explicitly, however a reference to ABIDE I	Yes	Yes	Low	Low	No information on the time interval or interventions has been provided, but a reference to ABIDE I and II is provided.	Yes	Unclear	Unclear	Unclear
**ElNakieb, Y. et al., 2019**	Participants are selected from NDAR. No further information on selection criteria is provided.	Unclear	Unclear	Unclear	Unclear	Data was obtained from NDAR. No information is provided on selected sites. A total of 263 participants were included of	Low	The diffusion features are the correlations between ROI's fractional anisotropy, mean diffusivity, axial diffusivity,	Yes	Yes	Low	Low	No information on the reference standard was provided in this paper explicitly, however a reference to NDAR	Yes	Yes	Low	Low	No information on the time interval or interventions has been provided, but a reference to NDAR is provided. There is	Yes	Unclear	Yes	Low
**Epalle, T. et al., 2021**	No patient selection criteria were applied. All subjects from the Preprocessed Connectomes Project (part of	Unclear	Yes	Unclear	Unclear	Data was obtained from ABIDE I. The sites caltech, CMU, KKI, KUL, maxmul, NYU, OHSU, Olin, Pitt. SBL. SDSU. Stanford. TCD.	Low	The functional features are statically obtained with 3 atlases: AAL90, CC200, and DOS160.The functional	Yes	No	Unclear	Low	No information on the reference standard was provided in this paper explicitly, however a reference to ABIDE I	Yes	Yes	Low	Low	No information on the time interval or interventions has been provided, but a reference to ABIDE I is provided. All	Yes	Unclear	Yes	Low
**Epalle, T. et al., 2019**	There is no information provided on patient selection criteria. The only information given is a reference to ABIDE.	Unclear	Unclear	Unclear	Unclear	Data was obtained from ABIDE. Two separate experiments are conducted. Experiment 1: the site Caltech	Low	The functional features are statically obtained with the AAL atlas. The regional connectivity features per ROI were obtained	Yes	Unclear	Unclear	Low	No information on the reference standard was provided in this paper explicitly, however a reference to ABIDE	Yes	Yes	Low	Low	No information on the time interval or interventions has been provided, but a reference to ABIDE is provided. All	Yes	Unclear	Yes	Low
**Fu, Y. et al., 2021**	Only male participants were selected between the ages of 6 and 34 because of the lack of female participants in the	Unclear	No	Unclear	Unclear	Data was obtained from ABIDE. The sites caltech, CMU, KKI, KUL1, KUL2, maxmul, NYU, OHSU. Olin. Pitt. SBL. SDSU.	Low	The structural features are radial distance and multivariate tensor-based morphometry per patch of the hippocampus	Yes	No	Unclear	Low	No information on the reference standard was provided in this paper explicitly, however a reference to ABIDE	Yes	Yes	Low	Low	No information on the time interval or interventions has been provided, but a reference to ABIDE is provided. All	Yes	Unclear	Yes	Low
**Gao, J. et al., 2021**	The participants were taken from ABIDE. No selection criteria are mentioned.	Unclear	Unclear	Unclear	Unclear	Data was obtained from ABIDE I. The sites caltech, CMU, KKI, KUL, maxmul, NYU, OHSU, Olin, Pitt. SBL. SDSU. Stanford. TCD.	Low	The structural features are structural connectivity matrices are obtained with the SRI24 atlas. where edges are	Yes	No	Unclear	Low	No information on the reference standard was provided in this paper explicitly, however a reference to ABIDE I	Yes	Yes	Low	Low	No information on the time interval or interventions has been provided, but a reference to ABIDE I is provided. All	Yes	Unclear	Yes	Low
**Gao, K. et al., 2021**	Participants are selected from two sites from NDAR. All participants in the dataset were about 24 months of age.	Unclear	Unclear	Unclear	Unclear	Data was obtained from NDAR. Two sites are selected for two separate experiments. These sites are IBIS and ACE. From	Low	The structural features are obtained with AI from segmentation and parcellation maps obtained with the infant	Yes	Unclear	Unclear	Low	No information on the reference standard was provided in this paper explicitly, however a reference to NDAR	Yes	Yes	Low	Low	No information on the time interval or interventions has been provided, but a reference to NDAR is provided. There is	Yes	Unclear	Yes	Low
**Guo, X. et al., 2022**	'The exclusion criteria were as follows: (1) MRI interpreted as abnormal (see attachment for details); (2) the quality of MR	Yes	Unclear	Yes	Low	Data was obtained from the Affiliated Hospital of Jining Medical University. A total of 347 participants were included	Low	Features for the structural and diffusion MRI modalities are both extracted from preprocessed versions of the	No	Unclear	High	Low	'The diagnosis of an ASD was based on DSM-IV criteria from a clinical interview and the completion of standardized	Yes	Yes	Low	Low	Participants were selected between January 2016 and December 2019. These participants were used in the	Yes	Yes	Yes	Low
**Haghighat, H. et al., 2022**	Participants were selected from the site NYU in ABIDE. The second sample of NYU in ABIDE II was excluded because	Unclear	No	Unclear	Unclear	Data was obtained from the site NYU for both ABIDE I and II (excluding sample 2 of ABIDE II). Experiment 1: a total of 30	Low	Experiment 1 : The functional features are dynamically obtained with no reported atlas. The causality between	Yes	Yes	Low	Low	No information on the reference standard was provided in this paper explicitly, however a reference to ABIDE I	Yes	Yes	Low	Low	No information on the time interval or interventions has been provided, but a reference to ABIDE I and II is provided.	Yes	Unclear	No	Unclear
**Han, Y. et al., 2021**	Children between the ages of 7 and 14 were included.	Unclear	Unclear	Yes	Unclear	The in-house dataset was collected at the University of Vermont. A total of 28 participants were included of	Low	The structural features are brain volume, brain surface area, cortical thickness, and cortical curvature obtained	Yes	Yes	Low	Low	The reported reference standard is behavioral assessments: ADOS-2, SCQ, CASL, UNIT-2, ToM Task	Yes	Yes	Low	Low	No information on the time interval or interventions has been provided. All patients that met the inclusion critera were	Yes	Yes	Yes	Low
**Hao, X. et al., 2022**	No inclusion or exclusion criteria are provided. It is only stated that 505 patients with ASD and 530 healthy controls	Unclear	Unclear	Unclear	Unclear	Data was obtained from 17 sites of ABIDE I. A total of 1035 participants were included of which 505 with ASD and 530	Low	Features were extracted using AI. Pearson's correlation between the time series of ROIs was used to form low-and-	Yes	Unclear	Unclear	Low	No information on the reference standard was provided in this paper explicitly, however a reference to ABIDE I	Yes	Yes	Low	Low	No information on the time interval or interventions has been provided, but a reference to ABIDE I is provided. There is	Yes	Unclear	Yes	Low
**Hu, J. et al., 2021**	Data were selected from the ABIDE datasets, specifically the Preprocessed Connectomes Project. No further inclusion	Unclear	Unclear	Unclear	Unclear	Data was obtained from ABIDE I. A total of 1035 participants were included of which 505 with ASD and 530 healthy	Low	Features were extracted using AI. Pearson's correlation coefficient between pairs of mean time series of ROIs was	Yes	No	Unclear	Low	No information on the reference standard was provided in this paper explicitly, however a reference to ABIDE I	Yes	Yes	Low	Low	No information on the time interval or interventions has been provided, but a reference to ABIDE I is provided. There is	Yes	Unclear	Yes	Low
**Hu, J. et al., 2020**	Data were selected from the ABIDE datasets, specifically the Preprocessed Connectomes Project. No further inclusion	Unclear	Unclear	Unclear	Unclear	Data was obtained from ABIDE I. There are 17 sites included. A total of 871 participants were included of which 403 with ASD	Low	The functional features are statically obtained with the AAL atlas. The functional connectivity features were	Yes	No	Unclear	Low	No information on the reference standard was provided in this paper explicitly, however a reference to ABIDE I	Yes	Yes	Low	Low	No information on the time interval or interventions has been provided, but a reference to ABIDE I is provided. There is	Yes	Unclear	Yes	Low

**Hu, Y. et al., 2022**	Participants were taken from ABIDE I and ABIDE II. There is no further information on selection criteria.	Unclear	Unclear	Unclear	Unclear	Data was obtained from ABIDE I and II. Multiple sites are selected. A total of 1035 participants were included	Low	Features were extracted using AI. A random selection per ROI was used to extract crops of timeseries which were used as	Yes	No	Unclear	Low	No information on the reference standard was provided in this paper explicitly, however a reference to ABIDE I	Yes	Yes	Low	Low	No information on the time interval or interventions has been provided, but a reference to ABIDE I and II is provided.	Yes	Unclear	Yes	Low
**Hu, Z. et al., 2022**	Patients were selected from three sites of ABIDE: UM2, OLIN, Leuven2. No information on selection criteria is	Unclear	Unclear	Unclear	Unclear	Data was obtained from ABIDE. The sites UM2, Olin and Leuven2 are selected. Included are respectively 34, 34, and 35	Low	The functional features are statically obtained with the AAL atlas. The functional connectivity features were	Yes	Unclear	Unclear	Low	No information on the reference standard was provided in this paper explicitly, however a reference to ABIDE	Yes	Yes	Low	Low	No information on the time interval or interventions has been provided, but a reference to ABIDE is provided. There is	Yes	Unclear	Yes	Low
**Huang, H. et al., 2018**	Participants are selected from a single site from ABIDE: NYU, to avoid the effects of multi-site data. No further selection	Unclear	No	Unclear	Unclear	Data was obtained from ABIDE. The site NYU is selected. A total of 92 participants were included of	Low	The functional features are statically obtained from ROIs specified by the AAL atlas. The multi-view group-sparse	Yes	Yes	Low	Low	No information on the reference standard was provided in this paper explicitly, however a reference to ABIDE	Yes	Yes	Low	Low	No information on the time interval or interventions has been provided, but a reference to ABIDE is provided. All	Yes	Unclear	Yes	Low
**Huang, Z. et al., 2020**	Patients are selected from the Preprocessed Connectomes Project of ABIDE I. There are no further selection criteria	Unclear	Unclear	Unclear	Unclear	Data was obtained from 17 sites of ABIDE I. A total of 1035 participants were included of which 505 with ASD and 530	Low	The functional features are statically obtained from ROIs specified by the CC200 atlas. The functional connectivity	Yes	No	Unclear	Low	No information on the reference standard was provided in this paper explicitly, however a reference to ABIDE I	Yes	Yes	Low	Low	No information on the time interval or interventions has been provided, but a reference to ABIDE I is provided. There is	Yes	Unclear	Yes	Low
**Huang, Z. et al., 2021**	Patients are selected from the Preprocessed Connectomes Project of ABIDE I. There are no further selection criteria	Unclear	Unclear	Unclear	Unclear	Data was obtained from ABIDE I. The sites KKI, KUL, SBL, TCD, NYU, OHSU, Olin, Pitt, UCLA, maxmul, CMU, USM, caltech,	Low	The functional features are statically obtained from ROIs specified by the CC200 atlas. The functional connectivity	Yes	No	Unclear	Low	No information on the reference standard was provided in this paper explicitly, however a reference to ABIDE I	Yes	Yes	Low	Low	No information on the time interval or interventions has been provided, but a reference to ABIDE I is provided. There is	Yes	Unclear	Yes	Low
**Ingalhalikar, M. et al., 2021**	Participants were selected from ABIDE I based on the following criteria. Excluded were individuals with: a	Unclear	No	Unclear	Unclear	Data was obtained from ABIDE I. The sites caltech, CMU, KKI, KUL1, KUL2, maxmul, NYU, Olin, Pitt, SBL, SDSU, TCD,	Low	The functional features are statically obtained from ROIs specified by the CC200 atlas. The functional connectivity	Yes	No	Unclear	Low	The reference standard are the criteria for ASD as per the DSM-IV.	Yes	Yes	Low	Low	No information on the time interval or interventions has been provided, but a reference to ABIDE I is provided. All	Yes	Yes	Yes	Low
**Irimia, A. et al., 2018**	Participants were selected at 4 separate sites according to the following selection criteria as mentioned in the paper.	Unclear	Yes	Yes	Low	Data was obtained from the sites Yale University, Harvard Medical School, University of Washington, University of	Low	The structural features are GM thickness, volume, and cortical area obtained with morphological computations	Yes	Yes	Low	Low	The reference standard is clinical observation based on the ADI, ADOS, and DSM-5 criteria.	Yes	Yes	Low	Low	There is no indication of a time interval and no record of interventions in between the reference standard and index	Yes	Yes	Yes	Low
**Jafadideh, A. and Asl, B., 2022**	Participants were selected from two sites of ABIDE II: SDSU and NYU1. The following selection criteria were used.	Unclear	No	Unclear	Unclear	Data was obtained from ABIDE II. The sites SDSU and NYU1 are selected for separate experiments. From SDSU, a	Low	Experiment 1: The functional features are statically obtained from ROIs specified by the Schaefer atlas. The graph	Yes	Unclear	Unclear	Low	No information on the reference standard was provided in this paper explicitly, however a reference to ABIDE	Yes	Yes	Low	Low	No information on the time interval or interventions has been provided, but a reference to ABIDE II is provided. All	Yes	Unclear	Yes	Low
**Jain, V. et al., 2022**	Participants were selected from ABIDE I and II. Patient were selected to minimize the age difference.	Unclear	No	No	High	Data was obtained from ABIDE I and II. No information on the selected site is provided. A total of 600 participants were	Low	The structural features are geometrical and Zernike moments features of the brain stem obtained through	Yes	Yes	Low	Low	The reference standard is classification based on the DSM-IV standards.	Yes	Yes	Low	Low	No information on the time interval or interventions has been provided, but a reference to ABIDE I and II is provided. All	Yes	Yes	Yes	Low
**Jha, R. et al., 2022**	Patients are selected from the Preprocessed Connectomes Project of ABIDE I. There are no further selection criteria	Unclear	Yes	Unclear	Unclear	Data was obtained from ABIDE. The sites KKI, KUL, SBL, TCD, NYU, OHSU, Olin, Pitt, UCLA, maxmul, CMU, USM,	Low	Features were extracted using AI. Averaging per ROI was used to extract mean time series per ROI which was used as input to	Yes	Unclear	Unclear	Low	No information on the reference standard was provided in this paper explicitly, however a reference to ABIDE	Yes	Yes	Low	Low	No information on the time interval or interventions has been provided, but a reference to ABIDE I is provided. There is	Yes	Unclear	Yes	Low
**Ji, J. and Yao, Y., 2021**	Participants were taken from the ABIDE dataset, specifically the Preprocessed Connectomes Project. 16	Unclear	Yes	Unclear	Unclear	Data was obtained from ABIDE I. The sites caltech, CMU, KKI, KUL, maxmul, NYU, OHSU, Olin, Pitt, SBL, SDSU, Stanford, TCD,	Low	Features were extracted using AI. Processing based on Pearson's correlation and the group-level coefficient and	Yes	Unclear	Unclear	Low	No information on the reference standard was provided in this paper explicitly, however a reference to ABIDE I	Yes	Yes	Low	Low	No information on the time interval or interventions has been provided, but a reference to ABIDE I is provided. All	Yes	Unclear	Yes	Low
**Ji, J. and Zhang, Y., 2022**	Participants were selected from ABIDE I and II. The following is a citation of selection criteria: 'It is worth	Unclear	No	Unclear	Unclear	Data was obtained from ABIDE I and II. From ABIDE I, 17 sites are selected. From ABIDE I, a specified total of 1096	Low	Features were extracted using AI. Pearson's correlation coefficients between pairs of ROIs was used to extract	Yes	Unclear	Unclear	Low	No information on the reference standard was provided in this paper explicitly, however a reference to ABIDE I	Yes	Yes	Low	Low	No information on the time interval or interventions has been provided, but a reference to ABIDE I and II is provided.	Yes	Unclear	Unclear	Unclear
**Ji, J. et al., 2020**	Data is taken from ABIDE I and II, but only the preprocessed section of the Preprocessed Connectomes Project. Subjects	Unclear	No	Unclear	Unclear	Data was obtained from 17 sites of ABIDE I and 16 sites of ABIDE II. ABIDE I: a total of 1096 participants were	Low	Pearson's correlation between pairs of ROIs was used to extract functional connectivity features. From the functional	Yes	Unclear	Unclear	Low	No information on the reference standard was provided in this paper explicitly, however a reference to ABIDE I	Yes	Yes	Low	Low	No information on the time interval or interventions has been provided, but a reference to ABIDE I and II is provided. All	Yes	Unclear	Yes	Low
**Jiang, W. et al., 2022**	Participants were selected from ABIDE I based on the following selection criteria. Subjects were excluded based	Unclear	No	Unclear	Unclear	Data was obtained from ABIDE I. The sites Caltech, CMU, KKI, Leuven, MaxMun, NYU, OHSU, Olin, Pitt, SBL, SDSU, Stanford,	Low	Features were extracted using AI. After preprocessing, 4D fMRI data was used as input for feature extraction and	No	Unclear	High	Low	No information on the reference standard was provided in this paper explicitly, however a reference to ABIDE I	Yes	Yes	Low	Low	No information on the time interval or interventions has been provided, but a reference to ABIDE I is provided. All	Yes	Unclear	Yes	Low
**Jung, M. et al., 2019**	Participants were taken from ABIDE according to the following selection criteria: 'The inclusion criteria were as	Unclear	No	Yes	Unclear	Data was obtained from ABIDE. No specific sites from ABIDE are mentioned but implicitly it is mentioned that	Low	The functional features are dynamically obtained from ROIs specified by the Desikan–Killiany atlas. The	Yes	Unclear	Unclear	Low	The reference standard is an ASD diagnosis based on DSM-IV-TR and assessed with ADOS.	Yes	Yes	Low	Low	No information on the time interval or interventions has been provided, but a reference to ABIDE is provided. All	Yes	Yes	Yes	Low
**Jung, W. et al., 2021**	Participants were selected from ABIDE. No inclusion or exclusion criteria are provided.	Unclear	Yes	Unclear	Unclear	Data was obtained from ABIDE. The sites UM, NYU, maxmul, OHSU, SBL, Olin, SDSU, caltech, TCD, Yale, Pitt,	Low	Features were extracted using AI from the upper diagonal part of functional connectivity matrices obtained by Fisher z-	Yes	No	Unclear	Low	No information on the reference standard was provided in this paper explicitly, however a reference to ABIDE	Yes	Yes	Low	Low	No information on the time interval or interventions has been provided, but a reference to ABIDE is provided. All	Yes	Unclear	Yes	Low
**Kang, E. et al., 2022**	Participants were selected from ABIDE I. Participants were excluded because of missing files and those with incomplete	Unclear	No	Unclear	Unclear	Data was obtained from ABIDE I from multiple sites of ABIDE I. A total of 985 participants were included of which 479	Low	Features were extracted using AI. Pearson's correlation coefficient between pairs of (sliding) windowed time series	Yes	Unclear	Unclear	Low	No information on the reference standard was provided in this paper explicitly, however a reference to ABIDE I	Yes	Yes	Low	Low	No information on the time interval or interventions has been provided, but a reference to ABIDE I is provided. All	Yes	Unclear	Yes	Low
**Karampasi, A. et al., 2021**	Participants were selected from ABIDE I according to the following selection criteria. Participants were included if	Unclear	No	Unclear	Unclear	Data was obtained from 17 sites of ABIDE I. A total of 871 participants were included of which 399 with ASD and 472	Low	The functional features are both statically and dynamically obtained from ROIs specified by the CC200 atlas. The	Yes	Yes	Low	Low	No information on the reference standard was provided in this paper explicitly, however a reference to ABIDE I	Yes	Yes	Low	Low	No information on the time interval or interventions has been provided, but a reference to ABIDE I is provided. All	Yes	Unclear	Yes	Low
**Kazeminejad, A. and Sotero, R., 2020**	Participants were taken from ABIDE I, specifically the sample preprocessed with the C-PAC pipeline. No further criteria are	Unclear	Unclear	Unclear	Unclear	Data was obtained from ABIDE I. The sites caltech, CMU, KKI, KUL, maxmul, NYU, OHSU, Olin, Pitt, SBL, SDSU, Stanford, TCD,	Low	The functional features are statically obtained from ROIs specified by the CC200 atlas. The graph features obtained	Yes	No	Unclear	Low	No information on the reference standard was provided in this paper explicitly, however a reference to ABIDE I	Yes	Yes	Low	Low	No information on the time interval or interventions has been provided, but a reference to ABIDE I is provided. Since	Yes	Unclear	Unclear	Unclear
**Kazeminejad, A. and Sotero, R., 2019**	Participants are selected from ABIDE I, specifically the Preprocessed Connectome Project. 'Any subject that had a	Unclear	No	Unclear	Unclear	Data was obtained from ABIDE I. Multiple sites are selected. A total of 109, 341, 186, 133, 45 participants were spread over	Low	The functional features are statically obtained from ROIs specified by the AAL atlas. The topological properties of brain	Yes	Unclear	Unclear	Low	No information on the reference standard was provided in this paper explicitly, however a reference to ABIDE I	Yes	Yes	Low	Low	No information on the time interval or interventions has been provided, but a reference to ABIDE I is provided.	Yes	Unclear	Unclear	Unclear
**Ke, F. and Yang, R., 2020**	Participants are selected from the site NYU of ABIDE. Selections are made based on image quality and registration	Unclear	No	Unclear	Unclear	Data was obtained from ABIDE. The site NYU is selected. A total of 39 participants were included of	Low	The structural features are extracted from whole images. No preprocessing or other processing is specified. The	Yes	Unclear	Unclear	Low	No information on the reference standard was provided in this paper explicitly, however a reference to ABIDE	Yes	Yes	Low	Low	No information on the time interval or interventions has been provided, but a reference to ABIDE is provided. All	Yes	Unclear	Yes	Low
**Ke, F. et al., 2021**	Some participants were excluded for insufficient image quality. Females were excluded because of sex imbalance in	Unclear	No	No	High	Data was obtained from ABIDE. The site is not specified, but judging by the size multiple sites were collected. A total of	Low	The structural features are cortical and subcortical tissue volume, cortical thickness and standard deviation, surface	Yes	Unclear	Unclear	Low	No information on the reference standard was provided in this paper explicitly, however a reference to ABIDE	Yes	Yes	Low	Low	No information on the time interval or interventions has been provided, but a reference to ABIDE is provided. All	Yes	Unclear	Yes	Low
**Khadem-Reza, Z. and Zare, H., 2022**	Participants are selected from the site NYU sample 1 from ABIDE II. Participants are selected between 5 and 10	Unclear	No	Unclear	Unclear	Data was obtained from ABIDE II. The site NYU is selected. A total of 52 participants were included of which 26 with ASD	Low	The structural features are volume and surface features of WM, GM, and CSF. Volumetric measures of WM and GM are	Yes	No	Unclear	Low	No information on the reference standard was provided in this paper explicitly, however a reference to ABIDE	Yes	Yes	Low	Low	No information on the time interval or interventions has been provided, but a reference to ABIDE II is provided. All	Yes	Unclear	Yes	Low
**Kim, J. et al., 2022**	Participants were enrolled between October 2015 and September 2019 at two sites in Seoul. Participants were	Yes	Yes	Yes	Low	An in-house database was created at the sites SNUH and HYUMC. A total of 106 participants were included of	Low	The structural features are cortical thickness, GM volume, surface area, mean curvature, subcortical volume based on	Yes	No	Unclear	Low	The reference standard are the criteria for ASD specified by the DSM-IV and ADOS	Yes	Yes	Low	Low	Participants were enrolled (scanned) between October 2015 and September 2019. Analyses of the scan data are	Yes	Yes	No	Low
**Kong, Y. et al., 2018**	Participants are selected from ABIDE I site NYU. No further selection criteria are reported.	Unclear	Yes	Unclear	Unclear	Data was obtained from ABIDE I. The site NYU is selected. A total of 182 participants were included of which 78 with ASD	Low	The structural features are structural connectivity matrices obtained through correlation based on the difference in GM	Yes	No	Unclear	Low	No information on the reference standard was provided in this paper explicitly, however a reference to ABIDE I	Yes	Yes	Low	Low	No information on the time interval or interventions has been provided, but a reference to ABIDE I is provided. All	Yes	Unclear	Yes	Low
**Lee, J. et al., 2021**	Participants were taken from multiple sites of ABIDE I. No selection criteria are described.	Unclear	Unclear	Unclear	Unclear	Data was obtained from ABIDE I. The sites caltech, KKI, KUL, maxmul, NYU, OHSU, Olin, Pitt, SBL, SDSU, Stanford, TCD,	Low	The functional features are statically obtained from ROIs specified by the AAL atlas. The functional connectivity	Yes	Unclear	Unclear	Low	No information on the reference standard was provided in this paper explicitly, however a reference to ABIDE I	Yes	Yes	Low	Low	No information on the time interval or interventions has been provided, but a reference to ABIDE I is provided. All	Yes	Unclear	Yes	Low
**Li, H. et al., 2018**	Participants were selected from 4 sites from ABIDE that had a sample size of at least 60. Healthy subjects were	Unclear	Unclear	Unclear	Unclear	Data was obtained from ABIDE. The sites UM, UCLA, USM, and KUL are selected for performance evaluation	Low	The functional features are statically obtained from ROIs specified by the AAL atlas. The functional connectivity	Yes	Unclear	Unclear	Low	No information on the reference standard was provided in this paper explicitly, however a reference to ABIDE	Yes	Yes	Low	Low	No information on the time interval or interventions has been provided, but a reference to ABIDE is provided. There is	Yes	Unclear	Yes	Low
**Li, J. et al., 2021**	Participants were selected from a preprocessed version of ABIDE. This includes multiple sites. No selection criteria are	Unclear	Yes	Unclear	Unclear	Data was obtained from ABIDE. The sites NYU, UM1, USM, UCLA1, Pitt, maxmul, Yale, KKI, TCD, Stanford,	Low	The functional features are statically obtained from ROIs specified by the massive online dictionary learning atlas. The	Yes	Unclear	Unclear	Low	No information on the reference standard was provided in this paper explicitly, however a reference to ABIDE	Yes	Yes	Low	Low	No information on the time interval or interventions has been provided, but a reference to ABIDE is provided. All	Yes	Unclear	Yes	Low
**Li, Q. et al., 2019**	Participants are selected from ABIDE according to the following citation from the paper: 'Subjects were excluded	Unclear	No	Yes	Unclear	Data was obtained from ABIDE. The sites caltech, CMU, KKI, KUL, maxmul, NYU, OHSU, Olin, Pitt, SBL, SDSU, Stanford,	Low	The functional features are statically obtained from ROIs specified by the Talairach Daemon atlas. The homotopic	Yes	Yes	Low	Low	No information on the reference standard was provided in this paper explicitly, however a reference to ABIDE	Yes	Yes	Low	Low	No information on the time interval or interventions has been provided, but a reference to ABIDE is provided. 29	Yes	Unclear	No	Unclear
**Liang, Y. et al., 2022**	Participants are selected from ABIDE. No specific selection criteria are reported but the following is stated: 'After data	Unclear	Unclear	Unclear	Unclear	Data was obtained from 17 sites of ABIDE. A total of 989 participants were included of which 480 with ASD and 509	Low	Fisher's z transformed Peason's correlation coefficients between pairs of ROIs were used to compute functional	Yes	Unclear	Unclear	Low	No information on the reference standard was provided in this paper explicitly, however a reference to ABIDE	Yes	Yes	Low	Low	No information on the time interval or interventions has been provided, but a reference to ABIDE is provided. All	Yes	Unclear	Yes	Low
**Liang, Y. et al., 2021**	Participants were selected from ABIDE, specifically the Preprocessed Connectomes Project. Participants with	Unclear	No	Unclear	Unclear	Data was obtained from ABIDE. The sites caltech, CMU, KKI, KUL, maxmul, NYU, OHSU, Olin, Pitt, SBL, SDSU, Stanford,	Low	Pearson's correlation coefficient / Kendall Rank correlation between the mean time series of pairs of ROIs was	Yes	Unclear	Unclear	Low	No information on the reference standard was provided in this paper explicitly, however a reference to ABIDE	Yes	Yes	Low	Low	No information on the time interval or interventions has been provided, but a reference to ABIDE is provided. All	Yes	Unclear	Yes	Low
**Liu, J. et al., 2020**	Participants are selected from ABIDE I. No specific selection criteria are reported but the sample is chosen to match	Unclear	Unclear	Unclear	Unclear	Data was obtained from ABIDE I. A total of 871 participants were included of which 403 with ASD and 468 healthy	Low	The functional features are dynamically obtained from ROIs specified by the AAL atlas. The functional connections	Yes	Unclear	Unclear	Low	No information on the reference standard was provided in this paper explicitly, however a reference to ABIDE I	Yes	Yes	Low	Low	No information on the time interval or interventions has been provided, but a reference to ABIDE I is provided. All	Yes	Unclear	Yes	Low
**Liu, R. et al., 2022**	Participants are selected from 17 sites of ABIDE I. There are no selection criteria described.	Unclear	Yes	Unclear	Unclear	Data was obtained from 17 sites of ABIDE I. A total of 1035 participants were included of which 505 with ASD and 530	Low	The functional features are statically obtained from ROIs specified by the CC200 atlas. The functional connectivity	Yes	Unclear	Unclear	Low	No information on the reference standard was provided in this paper explicitly, however a reference to ABIDE I	Yes	Yes	Low	Low	No information on the time interval or interventions has been provided, but a reference to ABIDE I is provided. All	Yes	Unclear	Yes	Low
**Liu, Y. et al., 2020**	Participants are selected from ABIDE, specifically the Preprocessed Connectomes Project. The paper mentions	Unclear	Unclear	Unclear	Unclear	Data was obtained from ABIDE. The sites caltech, CMU, KKI, KUL, maxmul, NYU, OHSU, Olin, Pitt, SBL, SDSU, Stanford,	Low	The functional features are statically obtained from ROIs specified by the CC200 atlas. The functional connectivity	Yes	No	Unclear	Low	No information on the reference standard was provided in this paper explicitly, however a reference to ABIDE	Yes	Yes	Low	Low	No information on the time interval or interventions has been provided, but a reference to ABIDE is provided. All	Yes	Unclear	Yes	Low
**Lu, Z. et al., 2021**	Participants are selected from 3 sites of ABIDE. No selection criteria are described.	Unclear	Yes	Unclear	Unclear	Data was obtained from ABIDE. The sites NYU, USM, UM1 are selected. A total of 391 participants were included	Low	Features were extracted using AI. A Pearson's correlation coefficient between the mean time series of pairs of ROIs was	Yes	Unclear	Unclear	Low	No information on the reference standard was provided in this paper explicitly, however a reference to ABIDE	Yes	Yes	Low	Low	No information on the time interval or interventions has been provided, but a reference to ABIDE is provided. All	Yes	Unclear	Yes	Low
**Mastrovito, D. et al., 2018**	Participants are selected from one site of ABIDE I and one site of ABIDE II. Participants younger than 18 are excluded	Unclear	No	Unclear	Unclear	Data was obtained from ABIDE I and II. Sites from ABIDE I: USM. Sites from ABIDE II: BNI. A total of 118 participants	Low	The functional features are statically obtained from ROIs specified by the Power atlas. The effective connectivity were	Yes	No	Unclear	Low	No information on the reference standard was provided in this paper explicitly, however a reference to ABIDE I	Yes	Yes	Low	Low	No information on the time interval or interventions has been provided, but a reference to ABIDE I and II is provided. All	Yes	Unclear	Yes	Low
**Mishra, M. and Pati, U., 2021**	Participants are selected from ABIDE I. There are no selection criteria reported.	Unclear	Unclear	Unclear	Unclear	Data was obtained from ABIDE I. The site None is selected. A total of 50 participants were included of which 26 with ASD	Low	The structural features are surface features of right hemisphere obtained with the Desikan-Killiany atlas through	Yes	Unclear	Unclear	Low	No information on the reference standard was provided in this paper explicitly, however a reference to ABIDE I	Yes	Yes	Low	Low	No information on the time interval or interventions has been provided, but a reference to ABIDE I is provided. All	Yes	Unclear	Yes	Low
**Mishra, M. and Pati, U., 2022**	It is only specified that they extracted a sample of ABIDE I from COINS without mention of any selection criteria.	Unclear	Unclear	Unclear	Unclear	Data was obtained from ABIDE I, without specifying a site. A total of 100 participants were included of which 68 with ASD	Low	The structural features are volumetric and morphometric features obtained with the Desikan-Killiany atlas through	Yes	Unclear	Unclear	Low	No information on the reference standard was provided in this paper explicitly, however a reference to ABIDE I	Yes	Yes	Low	Low	No information on the time interval or interventions has been provided, but a reference to ABIDE I is provided. There is	Yes	Unclear	Yes	Low
**Payabvash, S. et al., 2019**	The following are citations on patient selection: 'The participants in this study were recruited and prospectively	Yes	No	Yes	Low	Data was obtained from the Sensory Neurodevelopment and Autism Program neuroimaing collection. A total	Low	The diffusion features are Edge density obtained with the Desikan-Killiany atlas through probabilistic tractography	Yes	Unclear	Unclear	Low	The reference standard diagnosis is based on ADI-R, ADOS-G, SCQ, and DSM-IV criteria. It is also specified that	Yes	Yes	Low	Low	No information on the time interval or interventions has been provided. All patients that met the inclusion critera were	Yes	Yes	Yes	Low
**Peng, Liling and Liu, X. et al., 2022**	Participants are selected from ABIDE, site NYU. No selection criteria are reported	Unclear	Unclear	Unclear	Unclear	Data was obtained from ABIDE. The site NYU is selected. A total of 92 participants were included of	Low	The functional features are statically obtained from ROIs specified by the AAL atlas. The graph measures: connection	Yes	Unclear	Unclear	Low	No information on the reference standard was provided in this paper explicitly, however a reference to ABIDE	Yes	Yes	Low	Low	No information on the time interval or interventions has been provided, but a reference to ABIDE is provided. All	Yes	Unclear	Yes	Low
**Peng, Liang et al., 2022**	Participants are selected from ABIDE I and II but no selection criteria are provided.	Unclear	Unclear	Unclear	Unclear	Data was obtained from ABIDE I and II without specifying the sites. A total of 1029 participants were included of	Low	Pearson's correlation coefficients between windowed mean time series of pairs of ROIs were used to	Yes	No	Unclear	Low	No information on the reference standard was provided in this paper explicitly, however a reference to ABIDE I	Yes	Yes	Low	Low	No information on the time interval or interventions has been provided, but a reference to ABIDE I and II is provided. All	Yes	Unclear	Yes	Low
**Prasad, P. et al., 2022**	Participants are selected from ABIDE I. It is mentioned that 86 ASD scans were discarded because of missing time series.	Unclear	Unclear	Unclear	Unclear	Data was obtained from ABIDE I. The sites caltech, CMU, KKI, KUL, maxmul, NYU, OHSU, Olin, Pitt, SBL, SDSU, Stanford, TCD,	Low	Pearson's correlation coefficients between mean time series of pairs of ROIs were used to obtain functional	Yes	Unclear	Unclear	Low	No information on the reference standard was provided in this paper explicitly, however a reference to ABIDE I	Yes	Yes	Low	Low	No information on the time interval or interventions has been provided, but a reference to ABIDE I is provided.	Yes	Unclear	Unclear	Unclear
**Qiao, J. et al., 2022**	Participants are selected from ABIDE. Subjects with head movements larger than 2 mm and rotation angles larger than	Unclear	No	Unclear	Unclear	Data was obtained from ABIDE, without specifying which site. A total of 157 participants were included of	Low	The functional features are dynamically obtained from RSNs specified by no reported atlas. The functional	Yes	Unclear	Unclear	Low	No information on the reference standard was provided in this paper explicitly, however a reference to ABIDE	Yes	Yes	Low	Low	No information on the time interval or interventions has been provided, but a reference to ABIDE is provided. All	Yes	Unclear	Yes	Low

**Qin, C. et al., 2022**	Participants are selected from ABIDE I without specifying the sites. The following selection criteria are mentioned in the	Unclear	No	Unclear	Unclear	Data was obtained from ABIDE I, without specifying which site(s). A total of 472 participants were included of	Low	Algorithms are applied to mean time series per ROI was used to extract features. A combination of features was	No	Unclear	High	Low	No information on the reference standard was provided in this paper explicitly, however a reference to ABIDE I	Yes	Yes	Low	Low	No information on the time interval or interventions has been provided, but a reference to ABIDE I is provided. All	Yes	Unclear	Yes	Low
**Rakhimberdina, Z. et al., 2020**	It is specified that the selected participants are a subset of ABIDE. The selection criteria are not specified.	Unclear	Unclear	Unclear	Unclear	Data was obtained from 17 sites of ABIDE. A total of 871 participants were included of which 403 with ASD and 468	Low	The functional features are statically obtained from ROIs specified by no reported atlas. The graph signal processing	Yes	Unclear	Unclear	Low	No information on the reference standard was provided in this paper explicitly, however a reference to ABIDE	Yes	Yes	Low	Low	No information on the time interval or interventions has been provided, but a reference to ABIDE is provided. There is	Yes	Unclear	Yes	Low
**Rakic, M. et al., 2020**	Participants were selected from several sites of ABIDE I, specifically the Preprocessed Connectomes Project.	Unclear	No	Unclear	Unclear	Data was obtained from ABIDE I. The sites caltech, CMU, KKI, KUL, maxmul, NYU, OHSU, Olin, Pitt, SBL, SDSU, Stanford, TCD,	Low	Pearson's correlation coefficients between pairs of mean time series of ROIs were used to form static functional	Yes	No	Unclear	Low	No information on the reference standard was provided in this paper explicitly, however a reference to ABIDE I	Yes	Yes	Low	Low	No information on the time interval or interventions has been provided, but a reference to ABIDE I is provided. All	Yes	Unclear	Yes	Low
**Ronicko, J. et al., 2020**	Participants were selected from ABIDE I and II, without specifying which sites. The reported inclusion criteria are:	Unclear	No	Unclear	Unclear	Data was obtained from ABIDE I and II, without specifying which sites. A total of 600 participants were included of	Low	The functional features are statically obtained from ROIs specified by three atlases: Gordon's, Harvard-Oxford, and	Yes	Unclear	Unclear	Low	No information on the reference standard was provided in this paper explicitly, however a reference to ABIDE I	Yes	Yes	Low	Low	No information on the time interval or interventions has been provided, but a reference to ABIDE I and II is provided. All	Yes	Unclear	Yes	Low
**Saad, M. and Islam, S., 2019**	Participants are selected from the USC Multimodal Connectivity Database, which comes from the site UCLA. No	Unclear	Unclear	Unclear	Unclear	Data was obtained from USC Multimodal Connectivity Database, from which the site UCLA is selected. A total of 92	Low	The diffusion features are graph measures (e.g. assortativity and modularity) of connectivity matrices obtained	No	Yes	High	Low	No information on the reference standard was provided in this paper explicitly, however a reference to USC	Yes	Yes	Low	Low	No information on the time interval or interventions has been provided, but a reference to USC Multimodal	Yes	Unclear	Yes	Low
**Saponaro, S. et al., 2022**	Participants are selected from multiple sites of ABIDE I and II. The following selection criteria are reported. Participants (65)	Unclear	No	Unclear	Unclear	Data was obtained from 37 sites from ABIDE I and II. A total of 1638 participants were included of which 793 with ASD	Low	The structural features are thickness, volumes, and the mean and standard deviation of the thickness obtained with	Yes	Yes	Low	Low	No information on the reference standard was provided in this paper explicitly, however a reference to ABIDE I	Yes	Yes	Low	Low	No information on the time interval or interventions has been provided, but a reference to ABIDE I and II is provided. All	Yes	Unclear	Yes	Low
**Sen, B. et al., 2018**	Participants are selected from several sites of ABIDE. No selection criteria are reported.	Unclear	Yes	Unclear	Unclear	Data was obtained from ABIDE. The sites CMU, caltech, KKI, maxmul, NYU, Olin, OHSU, SDSU, NeuroIMAGE, Stanford,	Low	PCA and ICA were used to extract spatiotemporal features from preprocessed fMRI data. The structural	No	Unclear	High	Low	No information on the reference standard was provided in this paper explicitly, however a reference to ABIDE	Yes	Yes	Low	Low	No information on the time interval or interventions has been provided, but a reference to ABIDE is provided. It is	Yes	Unclear	Unclear	Unclear
**Shen, M. et al., 2018**	In this study a consecutive sample is described: 'Between July 20, 2007, and Dec 13, 2012, 159 children with autism	Yes	Unclear	Yes	Low	An in-house dataset was sampled at the site University of California. A total of 236 participants were included of	Low	The structural features are extra-axial CSF volume and total cerebral volume obtained with no reported atlas through	Yes	Yes	Low	Low	The reference standard is a diagnosis confirmed by ADOS and ADI-R. Furthermore, assessments based on the	Yes	Yes	Low	Low	There is no information provided on the time interval or any interventions between index test and reference	Yes	Yes	Yes	Low
**Sherkatghanad, Z. et al., 2020**	Participants are selected from multiple sites of ABIDE, specifically the Preprocessed Connectomes Project. The	Unclear	Unclear	Unclear	Unclear	Data was obtained from ABIDE I. The sites caltech, CMU, KKI, KUL, maxmul, NYU, OHSU, Olin, Pitt, SBL, SDSU, Stanford, UCLA,	Low	The functional features are statically obtained from ROIs specified by the CC400 atlas. The functional connectivity	Yes	Unclear	Unclear	Low	The reference standard is described for most of the included sites: 'The autism diagnostic observation tool and	Yes	Yes	Low	Low	No information on the time interval or interventions has been provided, but a reference to ABIDE I is provided. All	Yes	Unclear	Yes	Low
**Shi, C. et al., 2022**	Participants are selected from multiple sites of ABIDE, specifically the Preprocessed Connectomes Project. Sites	Unclear	No	Unclear	Unclear	Data was obtained from ABIDE. The sites NYU, UCLA, Pitt, Yale, USM, UM, TCD, KUL are selected. A total of 623	Low	The functional features are statically obtained from ROIs specified by the AAL atlas. The functional connectivity	Yes	No	Unclear	Low	No information on the reference standard was provided in this paper explicitly, however a reference to ABIDE	Yes	Yes	Low	Low	No information on the time interval or interventions has been provided, but a reference to ABIDE is provided. All	Yes	Unclear	Yes	Low
**Shi, C. et al., 2021**	Participants were selected from three sites of ABIDE. The following reasoning can be interpreted as selection	Unclear	No	Unclear	Unclear	Data was obtained from ABIDE. The sites NYU, USM, UM are selected. A total of 353 participants were included of	Low	The functional features are statically obtained from ROIs specified by the AAL atlas. The functional connectivity	Yes	No	Unclear	Low	No information on the reference standard was provided in this paper explicitly, however a reference to ABIDE	Yes	Yes	Low	Low	No information on the time interval or interventions has been provided, but a reference to ABIDE is provided. There is	Yes	Unclear	Yes	Low
**Sidhu, G., 2019**	Participants are selected from two sites of ABIDE. No selection criteria are provided.	Unclear	Unclear	Unclear	Unclear	Data was obtained from ABIDE. The sites Stanford and UM2 are selected and used separately. Stanford: a total of	Low	The functional features are obtained in a statical way. The processed time series were obtained through locally linear	No	Unclear	High	Low	No information on the reference standard was provided in this paper explicitly, however a reference to ABIDE	Yes	Yes	Low	Low	No information on the time interval or interventions has been provided, but a reference to ABIDE is provided. There is	Yes	Unclear	Yes	Low
**Song, Y. et al., 2019**	Participants are selected from five sites of ABIDE. No selection criteria are reported.	Unclear	Unclear	Unclear	Unclear	Data was obtained from ABIDE. The sites Stanford, KUL1, KUL2, Olin, Pitt, caltech are selected. A total of 235	Low	The functional features are statically obtained from ROIs specified by the AAL atlas. The metrics: modularity, global	Yes	Yes	Low	Low	No information on the reference standard was provided in this paper explicitly, however a reference to ABIDE	Yes	Yes	Low	Low	No information on the time interval or interventions has been provided, but a reference to ABIDE is provided. There is	Yes	Unclear	Yes	Low
**Spera, G. et al., 2019**	Participants were selected from four sites of ABIDE as a result of the following selection critera. Children in	Unclear	No	Unclear	Unclear	Data was obtained from ABIDE I. The sites KKI, NYU, UCLA, UM are selected. A total of 190 participants were included of	Low	The functional features are statically obtained from ROIs specified by the Harvard-Oxford atlas. The functional	Yes	No	Unclear	Low	The reference standard is not explicitely stated for this study, but it is reported that both ADOS Gotham total and ADOS	Yes	Yes	Low	Low	No information on the time interval or interventions has been provided, but a reference to ABIDE is provided. There is	Yes	Unclear	Yes	Low
**Squarcina, L. et al., 2021**	Participants were selected from an in-house dataset. There appear to be some selections based on the age of	Unclear	Unclear	Yes	Unclear	Data was obtained from in-house. The site La Nostra Famiglia, Scientific Institute IRCCS, Pasiandi Prato, Udine,	Low	The structural features are average cortical thickness normalized to cortical volume obtained with the Desikan-	Yes	Yes	Low	Low	The following is a citation of the information given in the paper on the reference standard: 'The ASD diagnosis	Yes	Yes	Low	Low	No information on the time interval is provided. The following is a citation of information on the	Yes	Yes	Yes	Low
**Subah, F. et al., 2021**	Participants were selected from multiple sites of ABIDE, specifically the preprocessed connectomes project.	Unclear	Unclear	Unclear	Unclear	Data was obtained from ABIDE. The sites caltech, CMU, KKI, KUL, maxmul, NYU, OHSU, Olin, Pitt, SBL, SDSU, Stanford,	Low	The functional features are statically obtained from ROIs specified by the BASC atlas. The functional connectivity	Yes	No	Unclear	Low	No information on the reference standard was provided in this paper explicitly, however a reference to ABIDE	Yes	Yes	Low	Low	No information on the time interval or interventions has been provided, but a reference to ABIDE is provided. All	Yes	Unclear	Yes	Low
**Sun, J. et al., 2021**	Participants are selected from four sites of ABIDE, but the machine learning experiment is only carried out on one site.	Unclear	No	Unclear	Unclear	Data was obtained from ABIDE. The site UCLA is selected. A total of 62 participants were included of	Low	The functional features are statically obtained from ROIs extracted using ICA. The mean z-values of ROIs based on RSNs	Yes	Unclear	Unclear	Low	No information on the reference standard was provided in this paper explicitly, however a reference to ABIDE	Yes	Yes	Low	Low	No information on the time interval or interventions has been provided, but a reference to ABIDE is provided. From the	Yes	Unclear	No	Unclear
**Supekar, K. et al., 2022**	Participants were selected from 14 sites of ABIDE for training and validation. Participants were included if	Unclear	No	Unclear	Unclear	Data was obtained from Stanford, GENDAAR, and 14 sites of ABIDE. From ABIDE, total of 834 participants were	Low	Features were extracted using AI from the time-averaged series per ROI. The phenotypic features gender, site, and age	Yes	Unclear	Unclear	Low	The reference standard reported for the datasets ABIDE, Stanford, and GENDAAR are ADOS and ADI-R. Though, it	Yes	Yes	Low	Low	No information on the time interval or interventions has been provided, but a reference to ABIDE, Stanford, and	Yes	Unclear	Yes	Low
**Tang, M. et al., 2020**	Participants are selected from multiple sites of ABIDE I. Participants (77) were removed because of low quality or	Unclear	No	Unclear	Unclear	Data was obtained from ABIDE I. The site 17 is selected. A total of 1035 participants were included of which 505 with ASD	Low	The correlation between the mean time series of pairs of ROIs, and between fMRI data and ROI time series were	Yes	Unclear	Unclear	Low	No information on the reference standard was provided in this paper explicitly, however a reference to ABIDE I	Yes	Yes	Low	Low	No information on the time interval or interventions has been provided, but a reference to ABIDE I is provided. All	Yes	Unclear	Yes	Low
**Wang, C. et al., 2019**	Participants were selected from 10 sites of ABIDE I, where the sample of the site NYU was taken from both ABIDE I and II.	Unclear	Unclear	Unclear	Unclear	Data was obtained from ABIDE I and II. Sites from ABIDE I: caltech, KUL, NYU, OHSU, Olin, Pitt, SBL, SDSU, USM, Yale,	Low	The functional features are statically obtained from ROIs specified by an atlas from Clements et al. (2018) The	Yes	Yes	Low	Low	No information on the reference standard was provided in this paper explicitly, however a reference to ABIDE I	Yes	Yes	Low	Low	No information on the time interval or interventions has been provided, but a reference to ABIDE I and II is provided. All	Yes	Unclear	Yes	Low
**Wang, C., 2021**	Participants are selected from multiple sites of ABIDE I and the included site NYU is also used from ABIDE II. Selection	Unclear	Yes	Unclear	Unclear	Data was obtained from ABIDE. Sites from ABIDE I: caltech, KUL, NYU, OHSU, Olin, Pitt, SBL, SDSU, USM, Yale, KKI,	Low	Pearson's correlation coefficient between mean time series of pairs of ROIs were computed to extract functional	Yes	Unclear	Unclear	Low	No information on the reference standard was provided in this paper explicitly, however a reference to ABIDE	Yes	Yes	Low	Low	No information on the time interval or interventions has been provided, but a reference to ABIDE is provided. All	Yes	Unclear	Yes	Low
**Wang, H. et al., 2022**	Participants were selected from the site NYU of ABIDE. There are no selection criteria provided for selection from this	Unclear	Yes	Unclear	Unclear	Data was obtained from ABIDE. The site NYU is selected. A total of 184 participants were included of	Low	The functional features are dynamically obtained from ROIs specified by the AAL atlas. The functional connectivity	Yes	Unclear	Unclear	Low	No information on the reference standard was provided in this paper explicitly, however a reference to ABIDE	Yes	Yes	Low	Low	No information on the time interval or interventions has been provided, but a reference to ABIDE is provided. There is	Yes	Unclear	Yes	Low
**Wang, J. et al. (2), 2019**	Participants are selected from two sites of ABIDE. No selection criteria are provided other than the statement that	Unclear	Yes	Unclear	Unclear	Data was obtained from the sites NYU and UM1 from ABIDE. NYU: A total of 182 participants were included of	Low	The functional features are statically obtained from ROIs specified by the AAL atlas. The functional connectivity and	Yes	No	Unclear	Low	No information on the reference standard was provided in this paper explicitly, however a reference to ABIDE	Yes	Yes	Low	Low	No information on the time interval or interventions has been provided, but a reference to ABIDE is provided. There is	Yes	Unclear	Yes	Low
**Wang, J. et al., 2019**	Participants are selected from three sites of ABIDE, specifically from the Preprocessed Connectomes	Unclear	Yes	Unclear	Unclear	Data was obtained from ABIDE. The sites NYU, USM, UM1 are selected. A total of 388 participants were included	Low	The functional features are statically obtained from ROIs specified by the AAL atlas. The functional connectivity and	Yes	Yes	Low	Low	No information on the reference standard was provided in this paper explicitly, however a reference to ABIDE	Yes	Yes	Low	Low	No information on the time interval or interventions has been provided, but a reference to ABIDE is provided. There is	Yes	Unclear	Yes	Low
**Wang, N. et al., 2022**	Participants were selected from 21 sites of ABIDE I and II. No selection criteria are reported.	Unclear	Unclear	Unclear	Unclear	Data was obtained from 21 sites of ABIDE I and II. A total of 609 participants were included of which 280 with ASD	Low	The functional features are statically obtained from ROIs specified by the BASC atlas. The functional connectivity	Yes	Yes	Low	Low	No information on the reference standard was provided in this paper explicitly, however a reference to ABIDE I	Yes	Yes	Low	Low	No information on the time interval or interventions has been provided, but a reference to ABIDE I and II is provided.	Yes	Unclear	Yes	Low
**Wang, Y. et al., 2020**	Participants were selected from multiple sites of ABIDE, specifically the Preprocessed Connectomes Project.	Unclear	No	Unclear	Unclear	Data was obtained from 17 sites of ABIDE. A total of 949 participants were included of which 419 with ASD and 530	Low	Pearson's correlation coefficients between the mean time series of pairs of ROIs were used to extract functional	Yes	Unclear	Unclear	Low	No information on the reference standard was provided in this paper explicitly, however a reference to ABIDE	Yes	Yes	Low	Low	No information on the time interval or interventions has been provided, but a reference to ABIDE is provided. There is	Yes	Unclear	Yes	Low
**Wong, E. et al., 2018**	Participants were selected from multiple sites of ABIDE, specifically from the Preprocessed Connectome	Unclear	Unclear	Unclear	Unclear	Data was obtained from 20 sites of ABIDE I. A total of 871 participants were included of which 403 with ASD and 468	Low	The functional features are statically obtained from ROIs specified by the Harvard-Oxford atlas. The functional	Yes	No	Unclear	Low	No information on the reference standard was provided in this paper explicitly, however a reference to ABIDE I	Yes	Yes	Low	Low	No information on the time interval or interventions has been provided, but a reference to ABIDE I is provided. There is	Yes	Unclear	Yes	Low
**Xiao, Z. et al., 2019**	Participants were selected from three sites of ABIDE II. No selection criteria are provided.	Unclear	Yes	Unclear	Unclear	Data was obtained from ABIDE II. The sites NYU, GU, KKI are selected. A total of 198 participants were included of	Low	ICA and min-max normalization were used to extract resting-state networks. Features were extracted from the resting-	Yes	Unclear	Unclear	Low	The reported reference standard is: 'All participants were diagnosed according to the score obtained in both the	Yes	Yes	Low	Low	No information on the time interval or interventions has been provided, but a reference to ABIDE II is provided. There is	Yes	Yes	Yes	Low
**Xing, X. et al., 2018**	Participants were selected from 17 sites of ABIDE, specifically from the Preprocessed Connectomes	Unclear	No	Unclear	Unclear	Data was obtained from 17 sites of ABIDE I. A total of 1096 participants were included of which 527 with ASD and 569	Low	Pearson's correlation coefficients between the mean time series of pairs of ROIs were used to extract functional	Yes	Unclear	Unclear	Low	No information on the reference standard was provided in this paper explicitly, however a reference to ABIDE I	Yes	Yes	Low	Low	No information on the time interval or interventions has been provided, but a reference to ABIDE I is provided. There is	Yes	Unclear	Yes	Low
**Xu, G. et al., 2022**	Participants were selected from multiple sites of ABIDE, specifically the Preprocessed Connectomes Project. The	Unclear	No	Unclear	Unclear	Data was obtained from 17 sites of ABIDE. A total of 963 participants were included of which 468 with ASD and 495	Low	Fisher's z-transformed Pearson's correlation coefficients between the windowed mean time series of	Yes	No	Unclear	Low	No information on the reference standard was provided in this paper explicitly, however a reference to ABIDE	Yes	Yes	Low	Low	No information on the time interval or interventions has been provided, but a reference to ABIDE is provided. All	Yes	Unclear	Yes	Low
**Yamin, M. et al., 2020**	Participants are selected from the UCLA Multimodal Connectivity Database. No selection criteria are reported.	Unclear	Unclear	Unclear	Unclear	Data was obtained from UCLA Multimodal Connectivity Database. A total of 79 participants were included of	Low	The functional features are statically obtained from ROIs specified by the Power atlas. The the distance to clusters of	Yes	Yes	Low	Low	No information on the reference standard was provided in this paper explicitly, however a reference to UCLA	Yes	Yes	Low	Low	No information on the time interval or interventions has been provided, but a reference to UCLA Multimodal	Yes	Unclear	Yes	Low
**Yang, C. et al., 2021**	Participants are selected from multiple sites of ABIDE I, specifically from the Preprocessed Connectomes	Unclear	Unclear	Unclear	Unclear	Data was obtained from multiple sites of ABIDE I. A total of 871 participants were included of which 403 with ASD	Low	Pearson's correlation coefficients between the mean time series of pairs of ROIs were used to form functional	Yes	Unclear	Unclear	Low	No information on the reference standard was provided in this paper explicitly, however a reference to ABIDE I	Yes	Yes	Low	Low	No information on the time interval or interventions has been provided, but a reference to ABIDE I is provided. All	Yes	Unclear	Yes	Low
**Yang, M. et al., 2021**	Participants were selected from the site NYU of ABIDE I. No selection criteria are reported.	Unclear	Yes	Unclear	Unclear	Data was obtained from ABIDE I. The site NYU is selected. A total of 184 participants were included of which 79 with ASD	Low	ICA and dual regression were used to extract subject-specific BFN spatial maps from preprocessed fMRI data.	Yes	Unclear	Unclear	Low	Though not explicitly stated, the ADOS scores are provided for the individuals with ASD, hinting that this is the	Yes	Yes	Low	Low	No information on the time interval or interventions has been provided, but a reference to ABIDE I is provided. There is	Yes	Yes	Yes	Low
**Yang, R. et al., 2021**	Participants are selected from the site NYU of ABIDE. No specific selection criteria are provided, but the following	Unclear	No	Unclear	Unclear	Data was obtained from ABIDE. The site NYU is selected. A total of 58 participants were included of	Low	The structural features are sMRI images obtained with no reported atlas through unspecified processing. The	Yes	Unclear	Unclear	Low	No information on the reference standard was provided in this paper explicitly, however a reference to ABIDE	Yes	Yes	Low	Low	No information on the time interval or interventions has been provided, but a reference to ABIDE is provided. All	Yes	Unclear	Yes	Low
**Yang, X. et al., 2020**	Participants were selected from multiple sites of ABIDE. Participants were selected if complete phenotypic	Unclear	No	Unclear	Unclear	Data was obtained from ABIDE. The sites caltech, CMU, KKI, KUL, maxmul, NYU, OHSU, Olin, Pitt, SBL, SDSU, Stanford,	Low	The functional features are statically obtained from ROIs specified by the CC400 atlas. The functional connectivity	Yes	No	Unclear	Low	No information on the reference standard was provided in this paper explicitly, however a reference to ABIDE	Yes	Yes	Low	Low	No information on the time interval or interventions has been provided, but a reference to ABIDE is provided. All	Yes	Unclear	Yes	Low
**Yi, T. et al., 2022**	A consecutive sample of participants between 2 and 5 years of age was collected between January 2019 and	Yes	No	Yes	Low	Data was obtained in-house. A total of 41 participants were included of which 24 with ASD and 17 healthy controls.	Low	An in-house dataset was used in this study. A total of 41 participants were included of which 24 with ASD and 17	Yes	Unclear	Unclear	Low	The following is reported on the reference standard: 'A total of 24 preschool children with ASD (18 male and 6 female.	Yes	Yes	Low	Low	No information on the time interval or interventions between reference standard and index test has been	Yes	Yes	Yes	Low
**Yin, W. et al., 2022**	Participants are selected from ABIDE I according to the sample obtained in other papers. No specific information	Unclear	Unclear	Unclear	Unclear	Data was obtained from multiple sites of ABIDE I. A total of 871 participants were included of which 403 with ASD	Low	Pearson's correlation coefficients between the mean time series of pairs of ROIs were used to extract functional	Yes	Yes	Low	Low	No information on the reference standard was provided in this paper explicitly, however a reference to ABIDE I	Yes	Yes	Low	Low	No information on the time interval or interventions has been provided, but a reference to ABIDE I is provided. All	Yes	Unclear	Yes	Low
**Yuan, D. et al., 2019**	Participants are selected from four sites of ABIDE. There are no reported selection criteria, but it is stated that all subjects	Unclear	Unclear	Unclear	Unclear	Data was obtained from four sites of ABIDE. The sites NYU, UCLA1, KKI, Yale are selected. A total of 216 participants	Low	The functional features are statically obtained from ROIs specified by no reported atlas. The funtional connections	Yes	Yes	Low	Low	No information on the reference standard was provided in this paper explicitly, however a reference to ABIDE	Yes	Yes	Low	Low	No information on the time interval or interventions has been provided, but a reference to ABIDE is provided. All	Yes	Unclear	Yes	Low
**Zhang, Fan et al., 2018**	A dataset enrolled at Center for Autism Research, Children's Hospital of Philadelphia was used in this study. Only male	Unclear	Unclear	Yes	Unclear	Data was obtained from the Center for Autism Research, Children's Hospital of Philadelphia. A total of 149	Low	The diffusion features are FA, MD, and the minimum, mean, and maximum of FA and MD for each fibre cluster obtained	Yes	No	Unclear	Low	The following statement is made with regard to the reference standard: 'The diagnoses were made with gold	Yes	Yes	Low	Low	No information on the time interval or interventions has been provided, but a reference to Center for Autism Research,	Yes	Yes	Yes	Low
**Zhang, Fangyu et al., 2022**	Participants were selected from multiple sites of ABIDE I. Specific selection criteria are not reported, but it is	Unclear	Unclear	Unclear	Unclear	Data was obtained from 17 sites from ABIDE I. A total of 1035 participants were included of which 505 with ASD	Low	Pearson's correlation coefficients between the mean time series of pairs of ROIs were used to extract functional	Yes	No	Unclear	Low	No information on the reference standard was provided in this paper explicitly, however a reference to ABIDE I	Yes	Yes	Low	Low	No information on the time interval or interventions has been provided, but a reference to ABIDE I is provided. All	Yes	Unclear	Yes	Low
**Zhang, J. et al., 2022**	Participants were selected from 17 sites of ABIDE I, specifically from the Preprocessed Connectomes	Unclear	Unclear	Unclear	Unclear	Data was obtained from ABIDE I. The sites caltech, CMU, KKI, KUL, maxmul, NYU, OHSU, Olin, Pitt, SBL, SDSU, Stanford, TCD,	Low	The functional features are statically obtained from ROIs specified by the CC200 atlas. The functional connectivity	Yes	No	Unclear	Low	No information on the reference standard was provided in this paper explicitly, however a reference to ABIDE I	Yes	Yes	Low	Low	No information on the time interval or interventions has been provided, but a reference to ABIDE I is provided. There is	Yes	Unclear	Yes	Low
**Zhang, Li et al., 2021**	Participants are selected from 5 sites of ABIDE, specifically from the Preprocessed Connectome Project. The	Unclear	No	Unclear	Unclear	Data was obtained from ABIDE. The sites NYU, UM, UCLA, USM, KUL are selected. A total of 474 participants	Low	The functional features are statically obtained from ROIs specified by the Harvard-Oxford atlas. The low-rank	Yes	Unclear	Unclear	Low	No information on the reference standard was provided in this paper explicitly, however a reference to ABIDE	Yes	Yes	Low	Low	No information on the time interval or interventions has been provided, but a reference to ABIDE is provided. All	Yes	Unclear	Yes	Low
**Zhang, Liangliang et al., 2020**	Participants are selected from the site USM of ABIDE I. The following selection criteria are reported: 'To avoid the effects	Unclear	No	Yes	Unclear	Data was obtained from ABIDE I. The site USM is selected. A total of 47 participants were included of which 21 with ASD	Low	The functional features are statically obtained from ROIs specified by the AAL atlas. The sample entropy was computed	Yes	Unclear	Unclear	Low	The reference standard used in this study is ADOS for a part of the sample. Furthermore, ABIDE I is referenced.	Yes	Yes	Low	Low	No information on the time interval or interventions has been provided, but a reference to ABIDE I is provided. All	Yes	Unclear	Yes	Low

**Zhang, M. et al., 2020**	Participants were selected from IMPAC, without specifying selection criteria and if selections were made.	Unclear	Unclear	Unclear	Unclear	Data was obtained from IMPAC, which comprises multiple sites. A total of 1127 participants were included of	Low	A SVD was used to extract singular values of each connectivity matrix per ROI. The structural features are	Yes	Unclear	Unclear	Low	No information on the reference standard was provided in this paper explicitly, however a reference to IMPAC	Yes	Yes	Low	Low	No information on the time interval or interventions has been provided, but a reference to IMPAC is provided. There	Yes	Unclear	Yes	Low
**Zhang, Y. et al., 2021**	Participants are selected from likely a single site from ABIDE, but it is not clearly specified. There are no selection criteria	Unclear	Unclear	Unclear	Unclear	Data was obtained from ABIDE, without specifying the site. A total of 50 participants were included of which 23 with ASD	Low	The functional features are statically obtained from ROIs specified by no reported atlas. The functional connectivity	Yes	Unclear	Unclear	Low	No information on the reference standard was provided in this paper explicitly, however a reference to ABIDE	Yes	Yes	Low	Low	No information on the time interval or interventions has been provided, but a reference to ABIDE is provided. There is	Yes	Unclear	Yes	Low
**Zhang, Z. et al., 2020**	Participants were selected from the site BNI of ABIDE II. The following is reported regarding selection criteria:	Unclear	No	Unclear	Unclear	Data was obtained from ABIDE II. The site BNI is selected. A total of 52 participants were included of which 26 with ASD	Low	The diffusion features are whole-brain fibre density, fibre bundle cross section, and a combination of the former and	Yes	Unclear	Unclear	Low	No information on the reference standard was provided in this paper explicitly, however a reference to ABIDE	Yes	Yes	Low	Low	No information on the time interval or interventions has been provided, but a reference to ABIDE II is provided. All	Yes	Unclear	Yes	Low
**Zhao, F. et al., 2018**	Participants were selected from the site NYU of ABIDE. The following is mentioned about the selection procedure:	Unclear	No	Unclear	Unclear	Data was obtained from ABIDE. The site NYU is selected. A total of 100 participants were included of	Low	The functional features are statically obtained from ROIs specified by the AAL atlas. The multi-level functional	Yes	Unclear	Unclear	Low	The reference standard used are the criteria provided in the DSM-IV-TR.	Yes	Yes	Low	Low	No information on the time interval or interventions has been provided, but a reference to ABIDE is provided. All	Yes	Yes	Yes	Low
**Zhao, F. et al., 2020**	Participants were selected from the site NYU of ABIDE. The following is reported regarding the selections made:	Unclear	No	Unclear	Unclear	Data was obtained from ABIDE. The site NYU is selected. A total of 92 participants were included of	Low	The functional features are dynamically obtained from ROIs specified by the AAL atlas. The (central moments of) multi-	Yes	Unclear	Unclear	Low	The reference standard used are the criteria provided in the DSM-IV-TR.	Yes	Yes	Low	Low	No information on the time interval or interventions has been provided, but a reference to ABIDE is provided. All	Yes	Yes	Yes	Low
**Zhao, F. et al., 2022**	Participants were selected from the site NYU of ABIDE. The following is reported regarding the selection criteria:	Unclear	No	Unclear	Unclear	Data was obtained from ABIDE. The site NYU is selected. A total of 92 participants were included of	Low	The functional features are dynamically obtained from ROIs specified by the AAL atlas. The high- and low order	Yes	Unclear	Unclear	Low	The ADOS and ADI-R scores are provided for the participants with ASD. It is specified that two individuals with ASD did	Yes	Yes	Low	Low	No information on the time interval or interventions has been provided, but a reference to ABIDE is provided. All	Yes	Yes	Yes	Low
**Zhao, M. et al., 2022**	Participants were selected from multiple sites of ABIDE. There are no selection criteria reported for the ABIDE	Unclear	Unclear	Unclear	Unclear	Data was obtained from ABIDE. A total of 1522 participants were included of which 743 with ASD and 779	Low	Independent component analysis was used to extract independent components from 4D fMRI data. Pearson's	Yes	Unclear	Unclear	Low	No information on the reference standard was provided in this paper explicitly, however a reference to ABIDE	Yes	Yes	Low	Low	No information on the time interval or interventions has been provided, but a reference to ABIDE is provided. There is	Yes	Unclear	Yes	Low
**Zu, C. et al., 2019**	From ABIDE site NYU, 45 individuals with ASD and 47 healthy controls are selected based on not specified criteria.	Unclear	Unclear	Unclear	Unclear	Data was obtained from ABIDE. The site NYU is selected. A total of 92 participants were included of	Low	The functional features are statically obtained from ROIs specified by the AAL atlas. The functional connectivity of	Yes	Unclear	Unclear	Low	No information on the reference standard was provided in this paper explicitly, however a reference to ABIDE	Yes	Yes	Low	Low	No information on the time interval or interventions has been provided, but a reference to ABIDE is provided. It is	Yes	Unclear	Unclear	Unclear
