# Supplementary material for: The diagnosis of ASD with MRI: a systematic review and meta-analysis
Source: Transl Psychiatry. 2024 Aug 2;14:318. doi: 10.1038/s41398-024-03024-5 (PMC11297045; doi:10.1038/s41398-024-03024-5)

Variable	ln DOR (95% CI)
**rsfMRI**	
Aghdam, M. et al. 2019 - 1	1.88(1.08 to 2.69)
Aghdam, M. et al. 2019 - 2	1.69(1.22 to 2.17)
Aghdam, M. et al. 2019 - 3	1.71(1.31 to 2.11)
Ahammed, S. et al. 2021 - 1	2.57(1.62 to 3.52)
Ahammed, S. et al. 2021 - 2	2.79(1.23 to 4.35)
Ahmed, R. et al. 2020 - 1	3.19(2.42 to 3.96)
Al-Hiyali, M. et al. 2021 - 1	4.24(2.76 to 5.71)
Al-Hiyali, M. et al. 2022 - 1	6.03(5.44 to 6.62)
Bhaumik, R. et al. 2018 - 1	0.84(0.42 to 1.26)
Bi, X. et al. 2020 - 1	5.84(4.32 to 7.36)
Byeon, K. et al. 2020 - 1	2.24(1.84 to 2.63)
Bernas, A. et al. 2018 - 1	4.42(1.53 to 7.31)
Cao, M. et al. 2021 - 1	2.06(1.75 to 2.37)
Cao, P. et al. 2022 - 1	1.52(1.24 to 1.81)
Chen, Zhihui et al. 2019 - 1	1.58(1.30 to 1.86)
Chu, Y. et al. 2022 - 1	2.68(1.96 to 3.40)
Dammu, P. and Bapi, R. 2019 - 1	2.06(1.75 to 2.36)
Dekhil, O. et al. 2018 - 1	4.62(3.80 to 5.44)
Deng, X. et al. 2022 - 1	1.24(0.98 to 1.50)
Deng, X. et al. 2022 - 2	1.24(0.98 to 1.50)
Dvornek, C. et al. 2019 - 1	1.87(1.20 to 2.54)
Dvornek, C. et al. 2019 - 2	2.28(1.47 to 3.10)
Dvornek, C. et al. 2019 - 3	1.95(1.05 to 2.84)
Dvornek, C. et al. 2019 - 4	1.46(0.62 to 2.29)
El-Gazzar, A. et al. 2019 - 1	1.42(1.24 to 1.60)
Epalle, T. et al. 2021 - 1	2.53(2.23 to 2.82)
Epalle, T. et al. 2019 - 1	1.70(0.34 to 3.06)
Epalle, T. et al. 2019 - 2	2.07(0.70 to 3.45)
Haghighat, H. et al. 2022 - 1	3.54(1.48 to 5.60)
Haghighat, H. et al. 2022 - 2	4.54(2.16 to 6.91)
Hao, X. et al. 2022 - 1	1.86(1.59 to 2.13)
Hu, J. et al. 2021 - 1	1.55(1.28 to 1.81)
Hu, J. et al. 2020 - 1	1.66(1.37 to 1.95)
Hu, Y. et al. 2022 - 1	1.51(1.25 to 1.78)
Hu, Y. et al. 2022 - 2	1.49(1.24 to 1.74)
Hu, Z. et al. 2022 - 1	1.62(-0.08 to 3.32)
Hu, Z. et al. 2022 - 2	0.65(-0.69 to 2.00)
Hu, Z. et al. 2022 - 3	1.97(0.51 to 3.44)
Huang, H. et al. 2018 - 1	2.64(1.64 to 3.64)
Huang, Z. et al. 2020 - 1	1.57(1.31 to 1.83)
Huang, Z. et al. 2021 - 1	2.35(2.07 to 2.64)
Ingalhalikar, M. et al. 2021 - 1	1.80(1.52 to 2.09)
Jafadideh, A. and Asl, B. 2022 - 1	7.78(3.82 to 11.74)
Jafadideh, A. and Asl, B. 2022 - 2	4.14(2.42 to 5.86)
Jha, R. et al. 2022 - 1	2.46(2.17 to 2.75)
Ji, J. and Yao, Y. 2021 - 1	1.53(1.28 to 1.79)
Ji, J. and Zhang, Y. 2022 - 1	1.60(1.34 to 1.86)
Ji, J. and Zhang, Y. 2022 - 2	1.54(1.28 to 1.80)
Ji, J. et al. 2020 - 1	1.47(1.22 to 1.73)
Ji, J. et al. 2020 - 2	1.31(1.05 to 1.58)
Jiang, W. et al. 2022 - 1	1.93(1.63 to 2.23)
Jung, M. et al. 2019 - 1	2.37(1.72 to 3.02)
Jung, W. et al. 2021 - 1	1.62(1.36 to 1.89)
Kang, E. et al. 2022 - 1	1.64(1.37 to 1.91)
Karampasi, A. et al. 2021 - 1	2.38(2.07 to 2.70)
Kazeminejad, A. and Sotero, R. 2020 - 1	0.85(0.60 to 1.10)
Kazeminejad, A. and Sotero, R. 2019 - 1	3.63(2.52 to 4.74)
Kazeminejad, A. and Sotero, R. 2019 - 2	1.61(1.13 to 2.09)
Kazeminejad, A. and Sotero, R. 2019 - 3	2.49(1.80 to 3.18)
Kazeminejad, A. and Sotero, R. 2019 - 4	2.62(1.77 to 3.47)
Kazeminejad, A. and Sotero, R. 2019 - 5	5.42(3.08 to 7.76)
Lee, J. et al. 2021 - 1	1.29(1.03 to 1.54)
Li, H. et al. 2018 - 1	1.43(0.64 to 2.22)
Li, H. et al. 2018 - 2	1.01(0.07 to 1.94)
Li, H. et al. 2018 - 3	1.60(0.50 to 2.69)
Li, H. et al. 2018 - 4	1.51(0.45 to 2.58)
Li, J. et al. 2021 - 1	1.95(1.65 to 2.25)
Liang, Y. et al. 2022 - 1	1.47(1.20 to 1.73)
Liang, Y. et al. 2021 - 1	2.45(2.17 to 2.74)
Liu, J. et al. 2020 - 1	2.38(2.07 to 2.70)
Liu, Y. et al. 2020 - 1	1.90(1.63 to 2.17)
Lu, Z. et al. 2021 - 1	1.55(1.12 to 1.97)
Mastrovito, D. et al. 2018 - 1	3.50(2.38 to 4.61)
Peng, Liling and Liu, X. et al. 2022 - 1	3.04(1.98 to 4.09)
Peng, Liang et al. 2022 - 1	1.17(0.91 to 1.43)
Prasad, P. et al. 2022 - 1	2.15(1.85 to 2.45)
Qiao, J. et al. 2022 - 1	4.34(2.99 to 5.69)
Qin, C. et al. 2022 - 1	2.32(1.90 to 2.75)
Rakhimberdina, Z. et al. 2020 - 1	2.03(1.72 to 2.33)
Ronicko, J. et al. 2020 - 1	1.93(1.57 to 2.29)
Sherkatghanad, Z. et al. 2020 - 1	1.78(1.50 to 2.05)
Shi, C. et al. 2022 - 1	4.08(3.59 to 4.58)
Shi, C. et al. 2021 - 1	2.01(1.52 to 2.51)
Sidhu, G. 2019 - 1	1.65(0.10 to 3.20)
Sidhu, G. 2019 - 2	2.37(0.18 to 4.57)
Song, Y. et al. 2019 - 1	2.19(1.59 to 2.78)
Spera, G. et al. 2019 - 1	1.80(1.17 to 2.43)
Subah, F. et al. 2021 - 1	4.01(3.59 to 4.43)
Sun, J. et al. 2021 - 1	5.33(2.42 to 8.24)
Supekar, K. et al. 2022 - 1	2.57(2.24 to 2.90)
Tang, M. et al. 2020 - 1	3.09(2.66 to 3.53)
Wang, C. et al. 2019 - 1	4.49(3.91 to 5.07)
Wang, C. 2021 - 1	3.38(3.01 to 3.75)
Wang, H. et al. 2022 - 1	1.70(1.06 to 2.33)
Wang, J. et al. (2) 2019 - 1	2.06(1.38 to 2.74)
Wang, J. et al. (2) 2019 - 2	1.85(0.97 to 2.74)
Wang, J. et al. 2019 - 1	1.41(0.96 to 1.86)
Wang, N. et al. 2022 - 1	1.56(1.21 to 1.90)
Wang, Y. et al. 2020 - 1	2.25(1.95 to 2.56)
Wong, E. et al. 2018 - 1	2.05(1.73 to 2.37)
Xiao, Z. et al. 2019 - 1	6.47(4.94 to 7.99)
Xing, X. et al. 2018 - 1	1.40(1.15 to 1.65)
Xu, G. et al. 2022 - 1	1.49(1.22 to 1.76)
Yamin, M. et al. 2020 - 1	1.26(0.34 to 2.19)
Yang, C. et al. 2021 - 1	1.92(1.62 to 2.22)
Yang, M. et al. 2021 - 1	2.47(1.78 to 3.17)
Yang, X. et al. 2020 - 1	2.22(1.94 to 2.50)
Yin, W. et al. 2022 - 1	3.90(3.49 to 4.31)
Yuan, D. et al. 2019 - 1	1.25(0.67 to 1.82)
Zhang, Fangyu et al. 2022 - 1	2.54(2.25 to 2.84)
Zhang, J. et al. 2022 - 1	1.78(1.51 to 2.05)
Zhang, Li et al. 2021 - 1	2.11(1.69 to 2.52)
Zhang, Liangliang et al. 2020 - 1	2.46(1.12 to 3.81)
Zhang, Y. et al. 2021 - 1	4.09(2.37 to 5.81)
Zhao, F. et al. 2018 - 1	2.82(1.84 to 3.80)
Zhao, F. et al. 2020 - 1	3.02(1.97 to 4.07)
Zhao, F. et al. 2022 - 1	2.90(1.86 to 3.94)
Zhao, M. et al. 2022 - 1	1.93(1.71 to 2.16)
Zu, C. et al. 2019 - 1	3.85(2.64 to 5.07)
Summary estimate, Q=215.132, df=118, I²=45.15%	2.20(2.06 to 2.33)
**sMRI**	
Bilgen, I. et al. 2020 - 1	1.70(1.09 to 2.30)
Chen, T. et al. 2020 - 1	1.32(0.79 to 1.86)
Chen, X. et al. 2022 - 1	2.23(1.82 to 2.64)
Denier, N. et al. 2021 - 1	2.66(1.93 to 3.39)
Duan, Y. et al. 2022 - 1	1.94(1.36 to 2.53)
Fu, Y. et al. 2021 - 1	3.18(2.80 to 3.57)
Gao, J. et al. 2021 - 1	2.01(1.73 to 2.29)
Gao, K. et al. 2021 - 1	4.42(3.46 to 5.38)
Gao, K. et al. 2021 - 2	3.21(1.35 to 5.06)
Han, Y. et al. 2021 - 1	4.22(1.68 to 6.76)
Jain, V. et al. 2022 - 1	1.74(1.39 to 2.09)
Ke, F. and Yang, R. 2020 - 1	3.33(1.34 to 5.33)
Ke, F. et al. 2021 - 1	1.30(1.02 to 1.58)
Khadem-Reza, Z. and Zare, H. 2022 - 1	4.65(2.43 to 6.88)
Kong, Y. et al. 2018 - 1	4.69(3.60 to 5.78)
Mishra, M. and Pati, U. 2021 - 1	4.57(2.34 to 6.80)
Mishra, M. and Pati, U. 2022 - 1	2.66(1.62 to 3.70)
Saponaro, S. et al. 2022 - 1	0.98(0.78 to 1.18)
Shen, M. et al. 2018 - 1	2.30(1.66 to 2.93)
Squarcina, L. et al. 2021 - 1	3.47(2.17 to 4.76)
Yang, R. et al. 2021 - 1	3.40(1.97 to 4.83)
Yi, T. et al. 2022 - 1	5.91(3.06 to 8.75)
Summary estimate, Q=29.416, df=21, I²=28.611%	2.63(2.21 to 3.06)
**dMRI**	
ElNakieb, Y. et al. 2019 - 1	1.81(1.27 to 2.34)
Payabvash, S. et al. 2019 - 1	2.17(-0.20 to 4.53)
Saad, M. and Islam, S. 2019 - 1	2.20(1.23 to 3.16)
Zhang, Fan et al. 2018 - 1	2.70(1.88 to 3.51)
Zhang, Z. et al. 2020 - 1	2.00(0.77 to 3.22)
Summary estimate, Q=3.258, df=4, I²=0.0%	2.09(1.71 to 2.47)
**multimodal**	
Aghdam, M. et al. 2018 - 1	1.00(0.30 to 1.70)
Brahim, A. and Farrugia, N. 2020 - 1	0.86(0.58 to 1.13)
Chen, Y. et al. 2022 - 1	2.16(1.88 to 2.45)
Dekhil, O. et al. 2019 - 1	2.97(2.19 to 3.75)
Eill, A. et al. 2019 - 1	5.73(3.57 to 7.89)
Guo, X. et al. 2022 - 1	3.38(2.80 to 3.96)
Irimia, A. et al. 2018 - 1	5.96(4.57 to 7.34)
Kim, J. et al. 2022 - 1	4.21(2.94 to 5.48)
Li, Q. et al. 2019 - 1	4.14(3.71 to 4.56)
Liu, R. et al. 2022 - 1	1.80(1.53 to 2.07)
Rakic, M. et al. 2020 - 1	3.48(3.09 to 3.87)
Sen, B. et al. 2018 - 1	1.20(0.95 to 1.44)
Zhang, M. et al. 2020 - 1	1.73(1.46 to 1.99)
Summary estimate, Q=23.299, df=12, I²=48.497%	2.76(2.14 to 3.38)
**Overall summary**	
Summary estimate, Q=277.13, df=158, I²=42.987%	2.28(2.15 to 2.40)

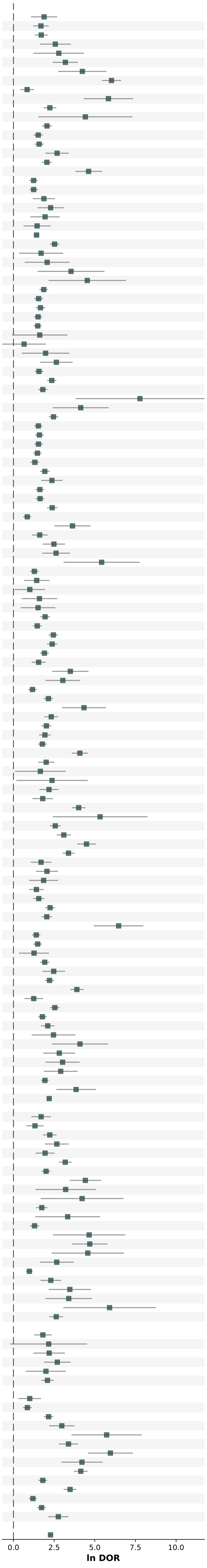

Variable	ln DOR (95% CI)
**Single-site**	
Ahammed, S. et al. 2021 - 1	2.57(1.62 to 3.52)
Ahammed, S. et al. 2021 - 2	2.79(1.23 to 4.35)
Ahmed, R. et al. 2020 - 1	3.19(2.42 to 3.96)
Bi, X. et al. 2020 - 1	5.84(4.32 to 7.36)
Chu, Y. et al. 2022 - 1	2.68(1.96 to 3.40)
Dvornek, C. et al. 2019 - 1	1.87(1.20 to 2.54)
Dvornek, C. et al. 2019 - 2	2.28(1.47 to 3.10)
Dvornek, C. et al. 2019 - 3	1.95(1.05 to 2.84)
Dvornek, C. et al. 2019 - 4	1.46(0.62 to 2.29)
Epalle, T. et al. 2019 - 1	1.70(0.34 to 3.06)
Epalle, T. et al. 2019 - 2	2.07(0.70 to 3.45)
Haghighat, H. et al. 2022 - 1	3.54(1.48 to 5.60)
Haghighat, H. et al. 2022 - 2	4.54(2.16 to 6.91)
Hu, Z. et al. 2022 - 1	1.62(-0.08 to 3.32)
Hu, Z. et al. 2022 - 2	0.65(-0.69 to 2.00)
Hu, Z. et al. 2022 - 3	1.97(0.51 to 3.44)
Huang, H. et al. 2018 - 1	2.64(1.64 to 3.64)
Jafadideh, A. and Asl, B. 2022 - 1	7.78(3.82 to 11.74)
Peng, Liling and Liu, X. et al. 2022 - 1	3.04(1.98 to 4.09)
Qiao, J. et al. 2022 - 1	4.34(2.99 to 5.69)
Sidhu, G. 2019 - 1	1.65(0.10 to 3.20)
Sidhu, G. 2019 - 2	2.37(0.18 to 4.57)
Sun, J. et al. 2021 - 1	5.33(2.42 to 8.24)
Wang, H. et al. 2022 - 1	1.70(1.06 to 2.33)
Wang, J. et al. (2) 2019 - 1	2.06(1.38 to 2.74)
Wang, J. et al. (2) 2019 - 2	1.85(0.97 to 2.74)
Yamin, M. et al. 2020 - 1	1.26(0.34 to 2.19)
Yang, M. et al. 2021 - 1	2.47(1.78 to 3.17)
Zhang, Liangliang et al. 2020 - 1	2.46(1.12 to 3.81)
Zhang, Y. et al. 2021 - 1	4.09(2.37 to 5.81)
Zhao, F. et al. 2018 - 1	2.82(1.84 to 3.80)
Zhao, F. et al. 2020 - 1	3.02(1.97 to 4.07)
Zhao, F. et al. 2022 - 1	2.90(1.86 to 3.94)
Zu, C. et al. 2019 - 1	3.85(2.64 to 5.07)
Gao, K. et al. 2021 - 1	4.42(3.46 to 5.38)
Gao, K. et al. 2021 - 2	3.21(1.35 to 5.06)
Han, Y. et al. 2021 - 1	4.22(1.68 to 6.76)
Ke, F. and Yang, R. 2020 - 1	3.33(1.34 to 5.33)
Khadem-Reza, Z. and Zare, H. 2022 - 1	4.65(2.43 to 6.88)
Kong, Y. et al. 2018 - 1	4.69(3.60 to 5.78)
Mishra, M. and Pati, U. 2021 - 1	4.57(2.34 to 6.80)
Mishra, M. and Pati, U. 2022 - 1	2.66(1.62 to 3.70)
Shen, M. et al. 2018 - 1	2.30(1.66 to 2.93)
Squarcina, L. et al. 2021 - 1	3.47(2.17 to 4.76)
Yang, R. et al. 2021 - 1	3.40(1.97 to 4.83)
Yi, T. et al. 2022 - 1	5.91(3.06 to 8.75)
Payabvash, S. et al. 2019 - 1	2.17(-0.20 to 4.53)
Saad, M. and Islam, S. 2019 - 1	2.20(1.23 to 3.16)
Zhang, Fan et al. 2018 - 1	2.70(1.88 to 3.51)
Zhang, Z. et al. 2020 - 1	2.00(0.77 to 3.22)
Dekhil, O. et al. 2019 - 1	2.97(2.19 to 3.75)
Eill, A. et al. 2019 - 1	5.73(3.57 to 7.89)
Guo, X. et al. 2022 - 1	3.38(2.80 to 3.96)
Summary estimate, Q=62.891, df=52, I^2=17.317%	2.79(2.52 to 3.06)
**Multi-site**	
Aghdam, M. et al. 2019 - 1	1.88(1.08 to 2.69)
Aghdam, M. et al. 2019 - 2	1.69(1.22 to 2.17)
Aghdam, M. et al. 2019 - 3	1.71(1.31 to 2.11)
Al-Hiyali, M. et al. 2021 - 1	4.24(2.76 to 5.71)
Al-Hiyali, M. et al. 2022 - 1	6.03(5.44 to 6.62)
Bhaumik, R. et al. 2018 - 1	0.84(0.42 to 1.26)
Byeon, K. et al. 2020 - 1	2.24(1.84 to 2.63)
Bernas, A. et al. 2018 - 1	4.42(1.53 to 7.31)
Cao, M. et al. 2021 - 1	2.06(1.75 to 2.37)
Cao, P. et al. 2022 - 1	1.52(1.24 to 1.81)
Chen, Zhihui et al. 2019 - 1	1.58(1.30 to 1.86)
Dammu, P. and Bapi, R. 2019 - 1	2.06(1.75 to 2.36)
Dekhil, O. et al. 2018 - 1	4.62(3.80 to 5.44)
Deng, X. et al. 2022 - 1	1.24(0.98 to 1.50)
Deng, X. et al. 2022 - 2	1.24(0.98 to 1.50)
El-Gazzar, A. et al. 2019 - 1	1.42(1.24 to 1.60)
Epalle, T. et al. 2021 - 1	2.53(2.23 to 2.82)
Hao, X. et al. 2022 - 1	1.86(1.59 to 2.13)
Hu, J. et al. 2021 - 1	1.55(1.28 to 1.81)
Hu, J. et al. 2020 - 1	1.66(1.37 to 1.95)
Hu, Y. et al. 2022 - 1	1.51(1.25 to 1.78)
Hu, Y. et al. 2022 - 2	1.49(1.24 to 1.74)
Huang, Z. et al. 2020 - 1	1.57(1.31 to 1.83)
Huang, Z. et al. 2021 - 1	2.35(2.07 to 2.64)
Ingalhalikar, M. et al. 2021 - 1	1.80(1.52 to 2.09)
Jafadideh, A. and Asl, B. 2022 - 2	4.14(2.42 to 5.86)
Jha, R. et al. 2022 - 1	2.46(2.17 to 2.75)
Ji, J. and Yao, Y. 2021 - 1	1.53(1.28 to 1.79)
Ji, J. and Zhang, Y. 2022 - 1	1.60(1.34 to 1.86)
Ji, J. and Zhang, Y. 2022 - 2	1.54(1.28 to 1.80)
Ji, J. et al. 2020 - 1	1.47(1.22 to 1.73)
Ji, J. et al. 2020 - 2	1.31(1.05 to 1.58)
Jiang, W. et al. 2022 - 1	1.93(1.63 to 2.23)
Jung, M. et al. 2019 - 1	2.37(1.72 to 3.02)
Jung, W. et al. 2021 - 1	1.62(1.36 to 1.89)
Kang, E. et al. 2022 - 1	1.64(1.37 to 1.91)
Karampasi, A. et al. 2021 - 1	2.38(2.07 to 2.70)
Kazeminejad, A. and Sotero, R. 2020 - 1	0.85(0.60 to 1.10)
Kazeminejad, A. and Sotero, R. 2019 - 1	3.63(2.52 to 4.74)
Kazeminejad, A. and Sotero, R. 2019 - 2	1.61(1.13 to 2.09)
Kazeminejad, A. and Sotero, R. 2019 - 3	2.49(1.80 to 3.18)
Kazeminejad, A. and Sotero, R. 2019 - 4	2.62(1.77 to 3.47)
Kazeminejad, A. and Sotero, R. 2019 - 5	5.42(3.08 to 7.76)
Lee, J. et al. 2021 - 1	1.29(1.03 to 1.54)
Li, H. et al. 2018 - 1	1.43(0.64 to 2.22)
Li, H. et al. 2018 - 2	1.01(0.07 to 1.94)
Li, H. et al. 2018 - 3	1.60(0.50 to 2.69)
Li, H. et al. 2018 - 4	1.51(0.45 to 2.58)
Li, J. et al. 2021 - 1	1.95(1.65 to 2.25)
Liang, Y. et al. 2022 - 1	1.47(1.20 to 1.73)
Liang, Y. et al. 2021 - 1	2.45(2.17 to 2.74)
Liu, J. et al. 2020 - 1	2.38(2.07 to 2.70)
Liu, Y. et al. 2020 - 1	1.90(1.63 to 2.17)
Lu, Z. et al. 2021 - 1	1.55(1.12 to 1.97)
Mastrovito, D. et al. 2018 - 1	3.50(2.38 to 4.61)
Peng, Liang et al. 2022 - 1	1.17(0.91 to 1.43)
Prasad, P. et al. 2022 - 1	2.15(1.85 to 2.45)
Qin, C. et al. 2022 - 1	2.32(1.90 to 2.75)
Rakhimberdina, Z. et al. 2020 - 1	2.03(1.72 to 2.33)
Ronicko, J. et al. 2020 - 1	1.93(1.57 to 2.29)
Sherkatghanad, Z. et al. 2020 - 1	1.78(1.50 to 2.05)
Shi, C. et al. 2022 - 1	4.08(3.59 to 4.58)
Shi, C. et al. 2021 - 1	2.01(1.52 to 2.51)
Song, Y. et al. 2019 - 1	2.19(1.59 to 2.78)
Spera, G. et al. 2019 - 1	1.80(1.17 to 2.43)
Subah, F. et al. 2021 - 1	4.01(3.59 to 4.43)
Supekar, K. et al. 2022 - 1	2.57(2.24 to 2.90)
Tang, M. et al. 2020 - 1	3.09(2.66 to 3.53)
Wang, C. et al. 2019 - 1	4.49(3.91 to 5.07)
Wang, C. 2021 - 1	3.38(3.01 to 3.75)
Wang, J. et al. 2019 - 1	1.41(0.96 to 1.86)
Wang, N. et al. 2022 - 1	1.56(1.21 to 1.90)
Wang, Y. et al. 2020 - 1	2.25(1.95 to 2.56)
Wong, E. et al. 2018 - 1	2.05(1.73 to 2.37)
Xiao, Z. et al. 2019 - 1	6.47(4.94 to 7.99)
Xing, X. et al. 2018 - 1	1.40(1.15 to 1.65)
Xu, G. et al. 2022 - 1	1.49(1.22 to 1.76)
Yang, C. et al. 2021 - 1	1.92(1.62 to 2.22)
Yang, X. et al. 2020 - 1	2.22(1.94 to 2.50)
Yin, W. et al. 2022 - 1	3.90(3.49 to 4.31)
Yuan, D. et al. 2019 - 1	1.25(0.67 to 1.82)
Zhang, Fangyu et al. 2022 - 1	2.54(2.25 to 2.84)
Zhang, J. et al. 2022 - 1	1.78(1.51 to 2.05)
Zhang, Li et al. 2021 - 1	2.11(1.69 to 2.52)
Zhao, M. et al. 2022 - 1	1.93(1.71 to 2.16)
Chen, T. et al. 2020 - 1	1.32(0.79 to 1.86)
Chen, X. et al. 2022 - 1	2.23(1.82 to 2.64)
Denier, N. et al. 2021 - 1	2.66(1.93 to 3.39)
Duan, Y. et al. 2022 - 1	1.94(1.36 to 2.53)
Fu, Y. et al. 2021 - 1	3.18(2.80 to 3.57)
Gao, J. et al. 2021 - 1	2.01(1.73 to 2.29)
Jain, V. et al. 2022 - 1	1.74(1.39 to 2.09)
Ke, F. et al. 2021 - 1	1.30(1.02 to 1.58)
Saponaro, S. et al. 2022 - 1	0.98(0.78 to 1.18)
Aghdam, M. et al. 2018 - 1	1.00(0.30 to 1.70)
Brahim, A. and Farrugia, N. 2020 - 1	0.86(0.58 to 1.13)
Chen, Y. et al. 2022 - 1	2.16(1.88 to 2.45)
Irimia, A. et al. 2018 - 1	5.96(4.57 to 7.34)
Kim, J. et al. 2022 - 1	4.21(2.94 to 5.48)
Li, Q. et al. 2019 - 1	4.14(3.71 to 4.56)
Liu, R. et al. 2022 - 1	1.80(1.53 to 2.07)
Rakic, M. et al. 2020 - 1	3.48(3.09 to 3.87)
Sen, B. et al. 2018 - 1	1.20(0.95 to 1.44)
Zhang, M. et al. 2020 - 1	1.73(1.46 to 1.99)
Summary estimate, Q=198.448, df=103, I^2=48.097%	2.12(1.98 to 2.26)
**Summary**	
Summary estimate, Q=277.13, df=158, I^2=42.987%	2.28(2.15 to 2.40)

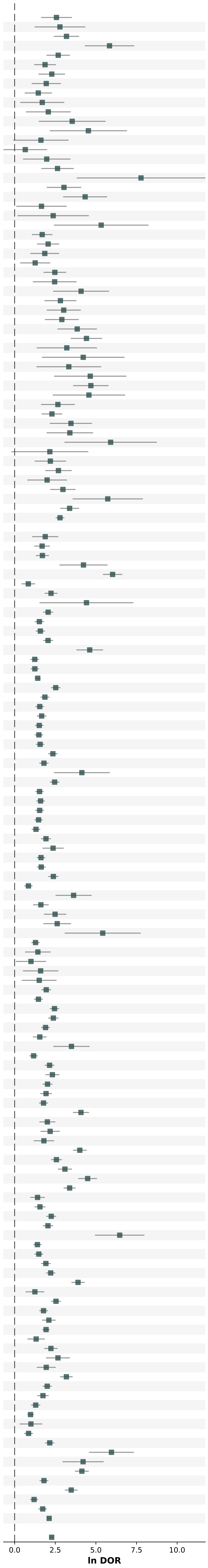

Supplement: Supplementary file 5 — Supplementary Materials C [file 41398_2024_3024_MOESM5_ESM.pdf]
