# Supplementary material for: The diagnosis of ASD with MRI: a systematic review and meta-analysis
Source: Transl Psychiatry. 2024 Aug 2;14:318. doi: 10.1038/s41398-024-03024-5 (PMC11297045; doi:10.1038/s41398-024-03024-5)
Supplement: Supplementary file 6 — PRISMA 2020 Checklist [file 41398_2024_3024_MOESM6_ESM.docx]

| **Section and Topic** | **Item #** | **Checklist item** | **Location where item is reported** |
| --- | --- | --- | --- |
| **TITLE** | | |  |
| Title | 1 | Identify the report as a systematic review. | Title |
| **ABSTRACT** | | |  |
| Abstract | 2 | See the PRISMA 2020 for Abstracts checklist. | Abstract, abstract checklist is given at the bottom of **this** document |
| **INTRODUCTION** | | |  |
| Rationale | 3 | Describe the rationale for the review in the context of existing knowledge. | fourth paragraph introduction |
| Objectives | 4 | Provide an explicit statement of the objective(s) or question(s) the review addresses. | Fifth paragraph introduction |
| **METHODS** | | |  |
| Eligibility criteria | 5 | Specify the inclusion and exclusion criteria for the review and how studies were grouped for the syntheses. | Section ‘Eligibility criteria’ |
| Information sources | 6 | Specify all databases, registers, websites, organisations, reference lists and other sources searched or consulted to identify studies. Specify the date when each source was last searched or consulted. | Section ‘Source and search’ |
| Search strategy | 7 | Present the full search strategies for all databases, registers and websites, including any filters and limits used. | Section ‘Source and Search’ |
| Selection process | 8 | Specify the methods used to decide whether a study met the inclusion criteria of the review, including how many reviewers screened each record and each report retrieved, whether they worked independently, and if applicable, details of automation tools used in the process. | Section ‘Selection and Collection’ |
| Data collection process | 9 | Specify the methods used to collect data from reports, including how many reviewers collected data from each report, whether they worked independently, any processes for obtaining or confirming data from study investigators, and if applicable, details of automation tools used in the process. | Section ‘Selection and Collection’ |
| Data items | 10a | List and define all outcomes for which data were sought. Specify whether all results that were compatible with each outcome domain in each study were sought (e.g. for all measures, time points, analyses), and if not, the methods used to decide which results to collect. | Section ‘Data Items’ |
|  | 10b | List and define all other variables for which data were sought (e.g. participant and intervention characteristics, funding sources). Describe any assumptions made about any missing or unclear information. | Section ‘Data Items’ |
| Study risk of bias assessment | 11 | Specify the methods used to assess risk of bias in the included studies, including details of the tool(s) used, how many reviewers assessed each study and whether they worked independently, and if applicable, details of automation tools used in the process. | Section ‘Study Assessment’ under Methods |
| Effect measures | 12 | Specify for each outcome the effect measure(s) (e.g. risk ratio, mean difference) used in the synthesis or presentation of results. | Section ‘Meta-analysis’ under Methods |
| Synthesis methods | 13a | Describe the processes used to decide which studies were eligible for each synthesis (e.g. tabulating the study intervention characteristics and comparing against the planned groups for each synthesis (item #5)). | Section ‘Meta-analysis’ under Methods |
|  | 13b | Describe any methods required to prepare the data for presentation or synthesis, such as handling of missing summary statistics, or data conversions. | Section ‘Meta-analysis’ under Methods |
|  | 13c | Describe any methods used to tabulate or visually display results of individual studies and syntheses. | Section ‘Meta-analysis’ under Methods |
|  | 13d | Describe any methods used to synthesize results and provide a rationale for the choice(s). If meta-analysis was performed, describe the model(s), method(s) to identify the presence and extent of statistical heterogeneity, and software package(s) used. | Section ‘Meta-analysis’ under Methods |
|  | 13e | Describe any methods used to explore possible causes of heterogeneity among study results (e.g. subgroup analysis, meta-regression). | Section ‘Meta-analysis’ under Methods |
|  | 13f | Describe any sensitivity analyses conducted to assess robustness of the synthesized results. | Section ‘Meta-analysis’ under Methods |
| Reporting bias assessment | 14 | Describe any methods used to assess risk of bias due to missing results in a synthesis (arising from reporting biases). | Section ‘Meta-analysis’ under Methods |
| Certainty assessment | 15 | Describe any methods used to assess certainty (or confidence) in the body of evidence for an outcome. | Section ‘Meta-analysis’ under Methods |
| **RESULTS** | | |  |
| Study selection | 16a | Describe the results of the search and selection process, from the number of records identified in the search to the number of studies included in the review, ideally using a flow diagram. | Section ‘Study Selection and Characteristics’ |
|  | 16b | Cite studies that might appear to meet the inclusion criteria, but which were excluded, and explain why they were excluded. | Section ‘Study Selection and Characteristics’ |
| Study characteristics | 17 | Cite each included study and present its characteristics. | Section ‘Study Selection and Characteristics’ |
| Risk of bias in studies | 18 | Present assessments of risk of bias for each included study. | Section ‘Study Assessment’ under results |
| Results of individual studies | 19 | For all outcomes, present, for each study: (a) summary statistics for each group (where appropriate) and (b) an effect estimate and its precision (e.g. confidence/credible interval), ideally using structured tables or plots. | Section ‘Meta-analysis’ under results |
| Results of syntheses | 20a | For each synthesis, briefly summarise the characteristics and risk of bias among contributing studies. | Section ‘Meta-analysis’ under results |
|  | 20b | Present results of all statistical syntheses conducted. If meta-analysis was done, present for each the summary estimate and its precision (e.g. confidence/credible interval) and measures of statistical heterogeneity. If comparing groups, describe the direction of the effect. | Section ‘Meta-analysis’ under results |
|  | 20c | Present results of all investigations of possible causes of heterogeneity among study results. | Section ‘Meta-analysis’ under results |
|  | 20d | Present results of all sensitivity analyses conducted to assess the robustness of the synthesized results. | Section ‘Meta-analysis’ under results |
| Reporting biases | 21 | Present assessments of risk of bias due to missing results (arising from reporting biases) for each synthesis assessed. | Section ‘Meta-analysis’ under results |
| Certainty of evidence | 22 | Present assessments of certainty (or confidence) in the body of evidence for each outcome assessed. | Section ‘Meta-analysis under results’ |
| **DISCUSSION** | | |  |
| Discussion | 23a | Provide a general interpretation of the results in the context of other evidence. | Section discussion |
|  | 23b | Discuss any limitations of the evidence included in the review. | Section discussion |
|  | 23c | Discuss any limitations of the review processes used. | Section discussion |
|  | 23d | Discuss implications of the results for practice, policy, and future research. | Section discussion |
| **OTHER INFORMATION** | | |  |
| Registration and protocol | 24a | Provide registration information for the review, including register name and registration number, or state that the review was not registered. | Abstract and section ‘Registration’ |
|  | 24b | Indicate where the review protocol can be accessed, or state that a protocol was not prepared. | Section ‘Registration’ |
|  | 24c | Describe and explain any amendments to information provided at registration or in the protocol. | Section ‘Registration’ and ‘Study Assessment’ under methods. |
| Support | 25 | Describe sources of financial or non-financial support for the review, and the role of the funders or sponsors in the review. | Section Funding |
| Competing interests | 26 | Declare any competing interests of review authors. | Section Conflict of Interests |
| Availability of data, code and other materials | 27 | Report which of the following are publicly available and where they can be found: template data collection forms; data extracted from included studies; data used for all analyses; analytic code; any other materials used in the review. | Section Data Availability |

*From:*  Page MJ, McKenzie JE, Bossuyt PM, Boutron I, Hoffmann TC, Mulrow CD, et al. The PRISMA 2020 statement: an updated guideline for reporting systematic reviews. BMJ 2021;372:n71. doi: 10.1136/bmj.n71

For more information, visit: <http://www.prisma-statement.org/>

PRISMA 2020 Abstract Checklist:

| 1. Identify the report as a systematic review | yes |
| --- | --- |
| 2. Provide an explicit statement of the main objective(s) or question(s) the review  addresses | yes |
| 3. Specify the inclusion and exclusion criteria for the review | yes |
| 4. Specify the information sources (such as databases, registers) used to identify  studies and the date when each was last searched | yes |
| 5. Specify the methods used to assess risk of bias in the included studies | yes |
| 6. Specify the methods used to present and synthesise results | yes |
| 7. Give the total number of included studies and participants and summarise  relevant characteristics of studies | yes |
| 8. Present results for main outcomes, preferably indicating the number of included  studies and participants for each. If meta-analysis was done, report the summary  estimate and confidence/credible interval. If comparing groups, indicate the  direction of the effect (that is, which group is favoured) | yes |
| 9. Provide a brief summary of the limitations of the evidence included in the review  (such as study risk of bias, inconsistency, and imprecision) | yes |
| 10. Provide a general interpretation of the results and important implications | yes |
| 11. Specify the primary source of funding for the review | Moved in manuscript editing |
| 12. Provide the register name and registration number | Moved in manuscript editing |
